# Educating early childhood care and education providers to improve knowledge and attitudes about reporting child maltreatment: A randomized controlled trial

**DOI:** 10.1371/journal.pone.0177777

**Published:** 2017-05-19

**Authors:** Ben Mathews, Chengwu Yang, Erik B. Lehman, Claudia Mincemoyer, Nicole Verdiglione, Benjamin H. Levi

**Affiliations:** 1 Australian Centre for Health Law Research, Queensland University of Technology, Brisbane, QLD, Australia; 2 College of Medicine, Department of Public Health Sciences, Division of Biostatistics and Bioinformatics, & Office for Scholarship in Learning and Education Research (OSLER), Penn State University, Hershey, PA, United States of America; 3 College of Medicine, Department of Public Health Sciences, Division of Biostatistics and Bioinformatics, Penn State University, Hershey, PA, United States of America; 4 Department of Agricultural Economics, Sociology and Education, Penn State University, University Park, PA, United States of America; 5 Pediatric Clinical Research Office, Penn State Hershey Children's Hospital, Hershey, PA, United States of America; 6 College of Medicine, Department of Pediatrics, Penn State University, Hershey, PA, United States of America; TNO, NETHERLANDS

## Abstract

Early childhood care and education providers (CCPs) work with over 7 million young children. These children are vulnerable to physical, sexual and emotional abuse, and neglect. However, CCPs make less than 1% of all reports of suspected child abuse and neglect that are made to child protective services. CCPs are therefore an untapped resource in the public health response to child maltreatment. However, their knowledge and attitudes about duties to report child maltreatment are poorly understood. Moreover, no rigorous research has tested whether their knowledge and attitudes about reporting child maltreatment can be improved. These gaps in knowledge are important because knowledge of the duty and positive attitudes towards it produce more effective reporting, and little evidence exists about how to enhance cognitive and affective attributes. Using the CONSORT approach, we report a single-blind test-retest randomized controlled trial evaluating *iLook Out for Child Abuse*, a customized online educational intervention for CCPs to increase knowledge and attitudes towards the reporting duty. 762 participants were randomized with results analyzed for 741 participants (372 in the intervention group; 369 in the control). Knowledge of the reporting duty increased in the intervention group from 13.54 to 16.19 out of 21 (2.65 increase, 95% CI: (2.37, 2.93); large effect size 0.95, p < 0.001); the control group remained stable, moving from 13.54 to 13.59 (0.05 increase, 95% CI: (-0.12, 0.22); negligible effect size 0.03, p = 0.684). Attitudes were enhanced on all 13 items for the intervention group, remaining stable in the control, with significant differences between groups on all items (p < 0.05). Gains were largely sustained at four month follow-up. Findings support education for CCPs and other professions. Future research should also explore effects of education on reporting behavior.

**Trial registration:** US National Institutes of Health NCT02225301

## Introduction

More than 680,000 children are involved in officially substantiated cases of abuse or neglect annually in the U.S. [1]. The youngest children are the most vulnerable, with 47% of all victims aged five and under, and nearly three quarters of fatalities involving children aged under three [1]. Evidence from population studies show the real incidence is substantially higher [2] [3]. This discrepancy arises for many reasons; most cases are not disclosed, not reported, or not investigated, or lack sufficient information to substantiate harm or to show the harm was caused by maltreatment [3].

A vast body of research has demonstrated the substantial physical, psychological, and behavioral harms of child maltreatment [4] [5] [6]. In the U.S., known fatalities exceed 1,640 per year, with 70% of these from neglect [1]. Sequelae are magnified with multiple types of trauma or poly-victimization within the same year [7]. Variance in sequelae is influenced by type and severity of maltreatment, and by individual and social factors, but the range of effects include physical injuries; failure to thrive; neurobiological impairment and genetic trauma; impaired social, emotional and behavioural development; reduced reading ability and perceptual reasoning; depression; anxiety; post-traumatic stress disorder; low self-image; alcohol and drug use; aggression; delinquency; long-term deficits in educational achievement; re-victimization; and adverse effects on employment and economic status [4] [5] [8] [9]. Socio-economic costs are estimated at $US124 billion per annum [10].

To identify cases of child maltreatment and enable early intervention to assist children and their families, many governments have passed mandatory reporting laws requiring designated persons to report suspected cases. A systematic review of U.S. state legislation shows CCPs—early childhood educators or practitioners, daycare providers, childcare workers, early childhood educators—are mandated reporters in the large majority of states, either being expressly named as mandated reporters, or because they are in a state where every person is a mandated reporter [11]. Taken together, all mandated reports of suspected maltreatment result in three quarters of substantiated cases of physical abuse and sexual abuse, and two-thirds of cases of substantiated psychological abuse and neglect [12]. Yet, while CCPs care for over 7 million children in the USA [13], they make less than 1.0% of all reports [1], and their reports identify only 0.5% of all substantiated cases [12].

The benefits of early identification are clear. Prompt identification enables interruption of maltreatment and avoidance of further harm. The sooner maltreatment is identified, the sooner it can be stopped, treatment provided, and rehabilitation and services offered to the child and her or his family. Even when reports do not lead to substantiation, they often result in referral to welfare agencies and service provision [1]. Especially for physical abuse, emotional abuse, and neglect, unsubstantiated reports do not differ markedly from substantiated reports in behavioral and developmental outcomes and service need [14]. Not all abuse can be substantiated, and conditions prompting warranted reports may involve precisely the kinds of at-risk situations in which social services can benefit children even when the threshold for abuse has not been met. Many reports even if unsubstantiated provide excellent opportunities for early intervention [14].

If mandated reporters are to be an effective part of a child protection strategy, they must receive appropriate education in three domains. First, expert education about the indicators of different forms of maltreatment is required [15] [16], as signs of maltreatment are not easily identifiable, and can mimic other medical conditions and normal childhood adversities. Second, education in the nature of the legislative reporting duties is required. The laws contain ambiguous concepts, so the precise nature of what has to be reported, and how and why, need to be clearly conveyed [11]. Third, education to foster attitudes conducive to appropriate reporting must also be implemented, since attitudes influence behaviour [17].

Though few systematic approaches to mandated reporting exist, it is known that professional education about child maltreatment should aim to develop knowledge, skills and attitudes leading to improved reporting. Better education about reporting is associated with self-reported preparedness to report [18], and awareness of reporting duties [19]. Lack of education is associated with low awareness of the reporting duty, low knowledge of indicators of abuse and self-reported lack of confidence identifying abuse [19] [20]. Effective reporting is thought to be influenced by knowledge of the duty, ability to recognise abuse, and positive attitudes about the duty [18] [19] [21] [22] [23].

### Rationale

The presence and quality of mandated reporter education is inconsistent across professional groups and jurisdictions. Numerous studies have found that members of various professions feel they have not had sufficient training to discharge their duty [16] [19] [20] [22] [24]. Research has identified low levels of knowledge in some professions about the reporting duty and attitudes precluding sound reporting [25] [26].

To date, educational interventions differ widely in delivery modality (paper materials only, in-person, or online), target audience (single or multiple professional group; pre-service or in-service), duration, approach (didactic or interactive; single discipline or multidisciplinary), content (one or multiple forms of maltreatment); and outcome measures [27]. Most educational programs focus only on cognitive skills, such as knowledge of indicators of child abuse, or of the content of the reporting duty, with less attention paid to the affective dimension of attitudinal development [27] [28]. Many have focused only on sexual abuse [29] [30].

Web-based training of professionals about child maltreatment has been adopted since the mid-2000s [31]. Delivery online offers multiple advantages: it is practicable; is cost-effective; enables access to large numbers of participants over broad geographic areas; enables flexible access in time and place; allows use of multi-media and multiple learning and delivery modes; and ensures program fidelity. Online education has been conducted and evaluated for knowledge gains with education and counselling students [31], nurses [32], and mixed audiences [33].

Educational programs on child abuse are rarely rigorously tested for effects, whether delivered online or traditionally. A systematic review of studies from 1994–2005 found only 15 programs met inclusion criteria against outcome measures of one or more of learning achievement, attitudinal change and clinical behaviour [34]. Only three used a control group, and these relied only on self-reported change in knowledge and confidence rather than objective measures [34]. Controlled trials outside 1994–2005 are rare [28] [32] [33]. Most programs have been directed towards professions such as medicine, nursing, dentistry, and school-based education [34]. Educational efforts for CCPs are rare, with isolated exceptions [33] [35]. Therefore, there is a pressing need to conduct true experimental studies of CCPs to identify baseline levels of knowledge and attitudes, and to explore changes produced by educational interventions.

### Objectives

CCPs are mandated reporters of child abuse and neglect under Pennsylvania law, and are in a unique position to identify and respond to these children. The evidence base is underdeveloped regarding CCPs’ knowledge of the law and attitudes towards the reporting duty, and there is also a lack of evidence about how to improve their knowledge and attitudes. This study was primarily designed to explore:

1. the baseline knowledge of CCPs regarding the legal reporting duty;
2. the baseline attitudes of CCPs towards the legal reporting duty;
3. whether knowledge of the duty is increased by a customized online educational program, and whether this is maintained over time;
4. whether attitudes towards the duty are enhanced by a customized online educational program, and whether this is maintained over time.

The overall aim of the experiment was to explore whether a multidisciplinary online educational program could improve these professionals’ knowledge of and attitudes towards reporting child maltreatment. This aim was achieved and the results demonstrated significant improvements in both domains.

## Method

### Design

In a sample of 762 childcare providers (CCPs), we randomized participants in a single-blind controlled trial. Using a test-retest design, we measured baseline knowledge of the legal reporting duty and attitudes towards reporting, and compared increase in knowledge and change to attitudes for those assigned to the intervention group (n = 388 completed the *iLookOut*) with the control group (n = 374). The study was approved by the Institutional Review Board of Penn State University on May 5, 2014 (#0376) and registered at the US National Institutes of Health #NCT02225301 https://clinicaltrials.gov/ct2/show/NCT02225301?term=NCT02225301&rank=1) (S1 File). The study was informed by the CONSORT approach [36] [37] and reports results accordingly (S1 Fig). The authors confirm that all ongoing and related trials for this intervention are registered. The short delay in registration of the trial was due to initial indications that since no clinical patients or health outcomes were being evaluated, registration may not be required; IRB advice was then provided to register the trial in any event and this advice was followed.

### Participants

Participants were CCPs from the state of Pennsylvania, which is the sixth most populous U.S. state (12.8 million), predominantly Caucasian (82%) [38], has a sizeable rural population (27%) and has a high median per capita income ($US46,000). Eligibility criteria included being at least 18 years of age, English-speaking, and working as paid or volunteer staff at a licensed childcare facility (commercial, non-commercial, home-based, or other) taking care of children under 5 years of age. Recruitment letters were sent to directors of all licensed childcare facilities in Pennsylvania via the mailing lists of Penn State Better Kid Care, which provides online professional development to 1,900 childcare facilities, the Pennsylvania Child Care Association, and Pennsylvania’s Office of Child Development and Early Learning. Childcare facilities were chosen by sampling respondents to obtain a representative distribution of participants, based on region of the state, rurality, and type of facility. Directors of childcare facilities were then provided with web links enabling their staff to access *iLookOut*. Participants provided informed written consent at the outset of *iLookOut*, and indicated willingness to be recontacted several months later for follow-up. No financial compensation was offered, but completion of *iLookOut* conferred two hours of professional development credit.

CCP directors were contacted about the study on May 22, 2014 and the date range for participant recruitment was June 23, 2014–July 18, 2014. Participants therefore accessed and completed *iLookOut* during a single four week period in June–July 2014. The *iLookOut* intervention was available 24 hours a day and could be paused and resumed as desired, across multiple sessions, in individuals’ residences or workplaces. Program software was designed to capture data about the participant’s responses. All data were de-identified and collected by the host website. The date range for the four month follow-up was December 8, 2014–February 20, 2015.

### Study intervention

The *iLookOut for Child Abuse*—*Online Learning Module for Early Childcare Providers* intervention was created in 2013–2014 by a multi-disciplinary team at the Center for the Protection of Children at the Penn State Children’s Hospital, involving experts in child abuse, instructional design, pediatrics, early childhood education, online learning, mandated reporter training, law, ethics, and child advocacy. Two co-investigators provided psychometric and statistical expertise. *iLookOut* was programmed using responsive web design to allow for mobile access, as well as features to accommodate individuals with sensory disabilities. *iLookOut* is hosted on a secure server by the Center for the Application of Information Technologies, which provides technological support for users, and stores all user data.

Content of *iLookOut* was informed by prior studies of professionals’ reporting [18] [20] [21] [22] [28] [31], and the professional expertise of the interdisciplinary team. The first four sections captured participant information about demographics; work information; knowledge about reporting child abuse and neglect, and attitudes towards reporting child abuse and neglect.

The fifth section of *iLookOut* comprised the online education program. This was designed to address standard cognitive aspects of mandated reporter training (e.g., definitions of abuse, signs of abuse, legal requirements for reporting), and to have a strong additional focus on affective elements thought to contribute to deeper understanding as well as effective practice. To do this, *iLookOut* employed an interactive, video-based storyline with films shot in point-of-view (i.e., the camera functioning as the learner’s eyes), with the learner taking the role of a teacher of 4 year olds at a childcare center. As key events unfold through interactions involving children, parents, and co-workers (all played by actors), the learner had to decide how to best respond. After some episodes, learners were posed questions, and based on their response were provided with didactic material to educate them about aspects of child abuse. Other videos were followed by questions where the learner had to choose how to respond to events. At different junctures in the story, learners could access resource files (e.g., Facts about Abuse, Red Flags for Abuse), and more information in text or video about the children. As in real life, the more information sought, the better informed would be the participant’s choices. The interactive storyline was designed to increase CCPs’ awareness about child abuse, and to help CCPs feel both empowered and responsible to contact CPS when there was a reasonable suspicion of child abuse. By immersing CCPs into real-life scenarios and requiring them to practice using their knowledge and skills, *iLookOut* was designed to help learners operationalize new information, and develop knowledge and attitudinal dispositions to help protect real children from harm.

#### Knowledge scale and attitudes scale

The knowledge scale (S2 File) comprised 21 items about the content of CCPs’ legal duty to report child abuse and neglect. Each item had one correct answer, enabling a maximum score of 21. The scale was informed by scales used in prior research [18] [19] [21] [28] to measure knowledge of key features of the duty for all kinds of maltreatment: what types and extents of abuse and neglect must be reported; what state of mind activates the duty to report; how, when, and to whom a report must be made; and legal protections. The scale was adapted to reflect Pennsylvania child protection law (Pennsylvania Consolidated Statutes, Title 23 (Domestic relations), ss 6303(a), 6303B, 6304, 6311, 6311B, 6313) including amendments in 2013 (House Bills 430, 436, 726), through analysis by the first author. This was cross-checked by discussion with the study team, and expert review by a panel comprising study investigators, the Director of the Center on Children and the Law at the Penn State Dickinson School of Law, two Pennsylvania legal practitioners specializing in child abuse law, and the Director for the Pennsylvania Department of Health and Human Services’ Bureau of Policy and Program Development, who screens policies for compliance with child abuse legislation.

The knowledge scale was piloted in a multi-stage process, involving: (1) a focus group with experienced CCPs and CCP educators (n = 7) to ensure clarity, comprehensibility and relevance; (2) cognitive interviews with three academic experts in child welfare law to ensure content validity; and (3) field-testing with a convenience sample of 60 early childhood education undergraduate students at Penn State University, and then with 38 local CCPs to evaluate and improve content validity and reliability, including test-retest reliability. In field testing, knowledge about each item was calculated by identifying the correct answer. A total score was calculated by summing correct answers. In post-test, items were randomized to reduce the possibility of repetition of initial answers and distorted outcomes [31].

The attitudes scale (S2 File) comprised 13 items exploring participants’ attitudes towards the duty to report child abuse and neglect. To accommodate jurisdictional context, this was adapted with permission from a previously validated scale constructed for similar purposes [23]. Items related to three salient attitudinal factors: *commitment* to the role of the professional in reporting; *confidence* in CPS to respond effectively; and *concerns* about consequences of reporting [23]. Each item invited responses using a 7-point Likert scale with options ranging from ‘Strongly Disagree‘ to ‘Strongly Agree‘. The attitudes scale was piloted in a two-stage process. First, a focus group with 7 experienced CCPs evaluated clarity, relevance and validity. This revealed no construct changes were required. Second, for reliability, we conducted a test-retest measurement with a convenience sample of 60 early childhood education students at Penn State University, and with 38 local CCPs. In retest, items were randomized to reduce the possibility of repetition of initial answers.

The host website randomized participants using an automated scheme to minimize the imbalance between the control and intervention groups for demographic and workplace characteristics: age (<30 vs. ≥30); parental status; education (degree vs. no degree); years worked as a CCP (<5 vs. ≥5), location (urban/suburban vs. rural); and number of children per facility (≤ 25 vs. >25). Participants were blinded to treatment allocation. Participants in the control group completed two iterations of the knowledge and attitudes scales, and subsequently undertook the *iLookOut* educational program. Participants in the intervention group completed one iteration of the knowledge and attitudes scales, received the *iLookOut* educational program, and then completed the second iteration of the knowledge and attitudes scales.

We first determined baseline levels of knowledge and attitudes. The primary outcomes were to measure the effect of completing *iLookOut* on changes in (1) knowledge, and (2) attitudes, by comparing the control and intervention groups. For the cohort participating in follow-up after 4 months, we measured whether knowledge and attitude gains were sustained over time.

### Sample size and statistical analysis

It was anticipated that small gains in knowledge may be present even post-intervention, due to child abuse reporting duties having existed in Pennsylvania for many years, high profile cases in the area having drawn broad public attention to child maltreatment and its reporting [39], and general educational efforts in this sector about child abuse. Accordingly, to detect a moderate effect size of Cohen’s d = 0.25 using two-sided two-sample t-test with 80% statistical power at type I error = 0.05 level, and assuming 15% drop-off, it was calculated that 300 participants for each study arm were needed. By comparison, sample sizes for the few controlled trials involving mandated reporter training have been much smaller [32] [33]. Another study involved a control group of 94 and an experimental group of 37 [28]. Our sample (n = 762) experienced low non-completion and provided a strong basis for statistical analysis.

All variables were summarized before analyses using descriptive statistics, means, medians, and standard deviations for continuous variables and frequencies and percentages for categorical variables. Distributions of continuous variables were assessed using histograms, box plots, normal probability plots, and the Shapiro-Wilk test for normality. Demographic variables were compared by intervention groups using Chi-square tests, and exact tests were used as needed. Statistical significance was set at *p <* 0.05. Baseline knowledge scores were calculated using descriptive statistics for each knowledge item and were aggregated to produce a mean score for each item and a mean total knowledge score. Baseline attitudes were calculated using descriptive statistics for each attitude item. Attitude items a, b, e, i, j, l, and m, which were negatively-worded, were reverse-coded so that for all attitude items a higher number represented a more positive response.

For the knowledge scores (individual items and total knowledge score), and for the attitude items, the change from baseline (pre-test) to re-test was calculated in the control group, while the change from baseline (pre-test) to post-test was calculated in the experimental group. A paired t-test was used to make comparisons between pre and post responses within each group, while a two-sample t-test was then applied to compare the mean change between the control and experimental groups. For the individual knowledge items, a generalized estimating equations (GEE) model was used to compare the change in the proportion of correct answers from pre-test or re-test to post-test between the control and experimental groups. A Cohen’s d statistic for means and a Cohen’s h statistic for proportions was calculated to further quantify the effect size of the change from pre-test to post-test, using 0.2, 0.5, and 0.8 as cut-off values for small, medium, and large effect sizes for both d and h. To rule out any bias introduced by pre-test differences between groups in knowledge or attitude items, Chi-square tests or two-sample t-tests were used to make group comparisons at pre-test, and no significant differences were found. In the follow-up cohort after 4 months, paired t-tests measured change in knowledge and attitudes. All analyses were performed using SAS version 9.4, and a significance level of 0.05 was used for all comparisons.

## Results

**Fig 1:**
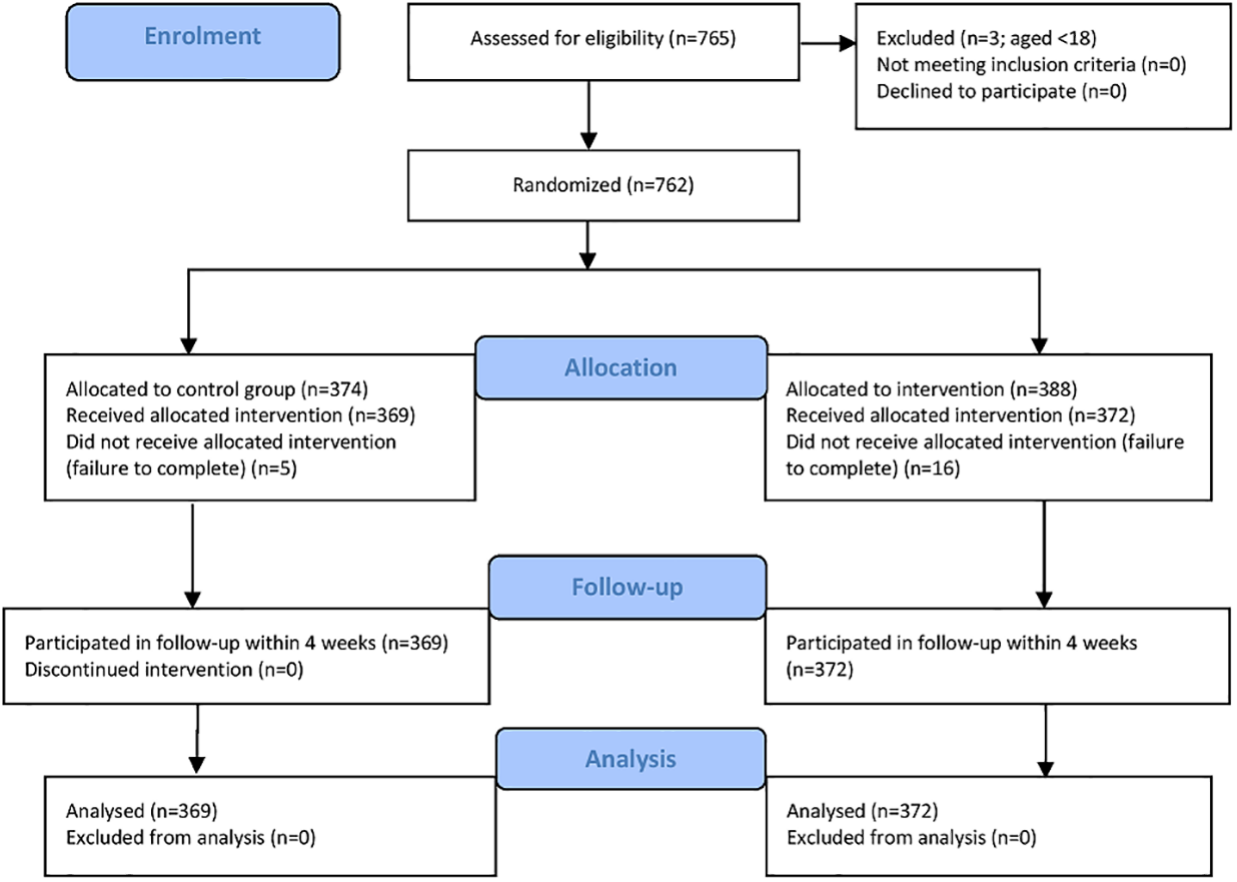
Participant flow diagram.

As shown in the participant flow diagram, 765 participants were recruited (Fig 1). Three were excluded because they were under 18 years of age. After randomization, 374 participants were assigned to the control group and 388 to the intervention group. Non-completion was low (3%), resulting in n = 369 participants in the control group and n = 372 participants in the experimental group. In addition, of the participants in the primary study, 460 agreed to be re-contacted four months later for a subsequent follow-up of knowledge and attitudes. Recontact with these individuals yielded n = 201 participants in the subsequent 4 month follow-up study. No adverse effects were reported.

Analysis was by originally assigned groups. Analysis by chi-square tests showed each group had comparable baseline demographics (Table 1). The entire sample was representative of the workforce [15]: 59.6% were aged over 30; 97.7% were female; 84.2% were non-Hispanic white; 30.9% had a Bachelor’s degree and 26.6% had a high school education; 72.1% were employed full-time and 22.8% were permanent part-time; 61.0% were parents or guardians; 72.2% worked in an urban or suburban setting; and 75.7% had at least 3 years’ experience as a practitioner. Notably, 78.5% had previously received training about child abuse and neglect.

**Table 1:** Demographic characteristics.

Variable		Total (N = 741)	Control (N = 369)	Experimental (N = 372)	P-value*
**Age**	18–29	299 (40.4)	148 (40.1)	151 (40.6)	0.742
	30–44	216 (29.1)	104 (28.2)	112 (30.1)	
	45+	226 (30.5)	117 (31.7)	109 (29.3)	
**Gender**	Male	17 (2.3)	13 (3.5)	3 (1.1)	0.026
	Female	724 (97.7)	356 (96.5)	368 (98.9)	
**Race/Ethnicity**	Non-Hispanic White	624 (84.2)	309 (83.7)	315 (84.7)	0.932
	Non-Hispanic Black	59 (8.0)	31 (8.4)	28 (7.5)	
	Hispanic	25 (3.4)	14 (3.8)	11 (3.0)	
	Non-Hispanic Asian	15 (2.0)	7 (1.9)	8 (2.1)	
	Non-Hispanic Other	18 (2.4)	8 (2.2)	10 (2.7)	
**Education**	High School	197 (26.6)	94 (25.5)	103 (27.7)	0.851
	CDA	101 (13.6)	52 (14.1)	49 (13.2)	
	Associates	149 (20.1)	75 (20.3)	74 (19.9)	
	Bachelors	229 (30.9)	112 (30.3)	117 (31.4)	
	Masters	65 (8.8)	36 (9.8)	29 (7.8)	
**Employment**	Permanent Full-Time	534 (72.1)	258 (69.9)	276 (74.2)	0.431
	Permanent Part-Time	169 (22.8)	90 (24.4)	79 (21.2)	
	Substitute Teacher	6 (0.8)	5 (1.4)	1 (0.3)	
	Seasonal	28 (3.8)	14 (3.8)	14 (3.8)	
	Volunteer	0 (0.0)	0 (0.0)	0 (0.0)	
	Other	4 (0.5)	2 (0.5)	2 (0.5)	
**Parent/Guardian**	Yes	452 (61.0)	225 (61.0)	227 (61.0)	0.990
	No	289 (39.0)	144 (39.0)	145 (39.0)	
**Previously Trained**	Yes	582 (78.5)	288 (78.1)	294 (79.0)	0.744
	No	159 (21.5)	81 (21.9)	78 (21.0)	
**Work Environment**	Rural	206 (27.8)	102 (27.6)	104 (28.0)	0.659
	Suburban	371 (50.1)	190 (51.5)	181 (48.6)	
	Urban	164 (22.1)	77 (20.9)	87 (23.4)	
**Work Setting**	Home	20 (2.7)	10 (2.7)	10 (2.7)	0.995
	Commercial	110 (14.8)	54 (14.6)	56 (15.1)	
	Non-Commercial	414 (55.9)	209 (56.7)	205 (55.1)	
	Head Start	62 (8.4)	30 (8.1)	32 (8.6)	
	Religious	102 (13.8)	51 (13.8)	51 (13.7)	
	Other	33 (4.4)	15 (4.1)	18 (4.8)	
**Years as Practitioner**	<1	68 (9.2)	39 (10.6)	29 (7.8)	0.820
	1–2	112 (15.1)	57 (15.4)	55 (14.8)	
	3–5	145 (19.6)	68 (18.4)	77 (20.7)	
	6–10	154 (20.8)	76 (20.6)	78 (21.0)	
	11–15	75 (10.1)	36 (9.8)	39 (10.5)	
	>15	187 (25.2)	93 (25.2)	94 (25.2)	

* Chi-square test, exact test used if needed

Baseline data showed each group had almost identical knowledge and attitudes. For knowledge, the mean baseline scores out of a possible 21 were 13.54 (95% CI = 13.27, 13.82) for the control group, and 13.54 (95% CI = 13.28, 13.80) for the intervention group (Table 2).

**Table 2:** Knowledge: Comparisons within and between control and experimental groups.

Variable	Control (N = 369)					Experimental (N = 372)					C v E
	Pre-test (baseline)	Re-test (no intervention)	Change	Effect Size	P-value	Pre-test (baseline)	Post-test after intervention	Change	Effect Size	P-value	P-value
Total score*	13.54 (13.27, 13.82)	13.59 (13.32, 13.86)	0.05 (-0.12, 0.22)	0.03	0.684	13.54 (13.28, 13.80)	16.19 (15.94, 16.42)	2.65 (2.37, 2.93)	0.95	<0.001	<0.001
Question 1a^†^	94.3	94.9	0.6	0.03	0.637	95.2	98.1	2.9	0.17	0.014	0.057
Question 1b^†^	93.5	94.6	1.1	0.05	0.371	94.4	97.0	2.6	0.13	0.013	0.168
Question 1c^†^	6.0	6.0	0.0	0.0	1.0	7.3	9.9	2.6	0.09	0.123	0.311
Question 1d^†^	91.9	95.7	3.8	0.16	0.011	90.6	97.8	7.2	0.33	<0.001	0.050
Question 1e^†^	93.0	95.9	2.9	0.13	0.043	90.3	98.7	8.4	0.40	<0.001	0.005
Question 1f^†^	93.8	97.0	3.2	0.16	0.008	90.1	98.7	8.6	0.41	<0.001	0.016
Question 1g^†^	8.1	6.5	-1.6	0.06	0.180	7.0	20.4	13.4	0.40	<0.001	<0.001
Question 2a^†^	83.2	85.4	2.2	0.06	0.157	84.1	96.8	12.7	0.46	<0.001	<0.001
Question 2b^†^	44.2	40.4	-3.8	0.08	0.051	44.6	55.1	10.5	0.21	<0.001	<0.001
Question 2c^†^	91.1	91.9	0.8	0.03	0.564	88.7	94.4	5.7	0.21	<0.001	0.022
Question 2d^†^	60.7	57.2	-3.5	0.07	0.052	58.3	59.7	1.4	0.03	0.612	0.129
Question 2e^†^	74.0	75.3	1.3	0.03	0.384	71.0	92.5	21.5	0.58	<0.001	<0.001
Question 3a^†^	44.2	46.1	1.9	0.04	0.262	48.7	82.8	34.1	0.74	<0.001	<0.001
Question 3b^†^	81.6	81.6	0.0	0.0	1.0	80.6	92.2	11.6	0.35	<0.001	<0.001
Question 3c^†^	47.7	49.1	1.4	0.03	0.411	45.2	84.9	39.7	0.87	<0.001	<0.001
Question 4^†^	39.6	41.2	1.6	0.03	0.460	35.5	40.9	5.4	0.11	0.058	0.288
Question 5^†^	64.0	63.1	-0.9	0.02	0.662	64.2	84.1	19.9	0.46	<0.001	<0.001
Question 6^†^	52.8	41.7	-11.1	0.22	<0.001	54.6	70.7	16.1	0.33	<0.001	<0.001
Question 7^†^	74.0	74.0	0.0	0.0	1.0	75.5	84.4	8.9	0.22	<0.001	0.003
Question 8^†^	45.5	46.1	0.6	0.01	0.670	54.3	62.1	7.8	0.16	<0.001	0.004
Question 9^†^	71.5	75.9	4.4	0.02	0.038	73.9	97.8	23.9	0.77	<0.001	<0.001

* Paired t-tests and Two-sample t-test for change from Pre-test to Re-test or Post-test (Re-Pre, Post-Pre): Mean (95% CI), Cohen’s d effect size

† Generalized Estimating Equations (GEE) model for repeated measures: % correct, Cohen’s h effect size

Total Knowledge score ranges from 0–21. Effect size: 0.2 = small, 0.5 = medium, 0.8 = large, for both Cohen’s d and Cohen’s h

For baseline attitudes, no meaningful differences were found between control and intervention groups (Table 3). Scores for only three of 13 items differed by > 0.1 (items b, c, and m), and no differences were > 0.22. On the 7-point Likert scale, differences of > 0.5 were classified as meaningful and used as a cut-off for comparisons.

**Table 3:** Attitudes: Comparisons within and between control and experimental groups.

Variable	Control (N = 369)					Experimental (N = 372)					C v E
	Pre-test (baseline)	Re-test (no intervention)	Change	Effect Size	P-value	Pre-test (baseline)	Post-test after intervention	Change	Effect Size	P-value	P-value
Attitude average *	5.78 (5.71, 5.86)	5.95 (5.87, 6.03)	0.17 (0.12, 0.21)	0.36	<0.001	5.80 (5.73, 5.87)	6.39 (6.33, 6.46)	0.59 (0.53, 0.65)	0.98	<0.001	<0.001
Question a*	5.81 (5.66, 5.97)	6.08 (6.95, 6.21)	0.27 (0.12, 0.41)	0.18	<0.001	5.86 (5.71, 6.01)	6.44 (6.33, 6.55)	0.58 (0.43, 0.74)	0.38	<0.001	0.004
Question b*	5.63 (5.48, 5.78)	5.86 (5.72, 6.0)	0.23 (0.09, 0.37)	0.16	0.002	5.74 (5.59, 5.88)	6.32 (6.21, 6.44)	0.58 (0.43, 0.74)	0.39	<0.001	<0.001
Question c*	6.29 (6.17, 6.41)	6.33 (6.22, 6.44)	0.04 (-0.08, 0.15)	0.04	0.595	6.14 (6.0, 6.28)	6.57 (6.48, 6.67)	0.43 (0.29, 0.57)	0.31	<0.001	<0.001
Question d*	6.09 (5.96, 6.23)	6.27 (6.16, 6.38)	0.17 (0.03, 0.32)	0.12	0.012	6.10 (5.97, 6.23)	6.65 (6.58, 6.72)	0.55 (0.43, 0.68)	0.45	<0.001	<0.001
Question e*	6.15 (6.03, 6.27)	6.11 (5.98, 6.24)	-0.04 (-0.17, 0.09)	0.03	0.549	6.13(6.01, 6.25)	6.43 (6.32, 6.55)	0.30 (0.16, 0.44)	0.22	<0.001	<0.001
Question f*	6.01 (5.88, 6.13)	6.17 (6.06, 6.29)	0.17 (0.05, 0.28)	0.15	0.012	6.0 (5.87, 6.13)	6.49 (6.40, 6.59)	0.49 (0.35, 0.63)	0.36	<0.001	<0.001
Question g*	6.27 (6.14, 6.40)	6.41 (6.30, 6.52)	0.14 (0.03, 0.25)	0.13	0.020	6.29 (6.16, 6.42)	6.60 (6.51, 6.69)	0.31 (0.19, 0.44)	0.25	<0.001	0.043
Question h*	5.59 (5.43, 5.74)	5.88 (5.74, 6.02)	0.29 (0.15, 0.43)	0.21	<0.001	5.61 (5.46, 5.76)	6.35 (6.24, 6.47)	0.74 (0.59, 0.90)	0.50	<0.001	<0.001
Question i*	5.06 (4.88, 5.25)	5.38 (5.20, 5.56)	0.32 (0.16, 0.48)	0.21	<0.001	5.04 (4.86, 5.22)	6.11 (5.96, 6.26)	1.07 (0.86, 1.28)	0.52	<0.001	<0.001
Question j*	6.09 (5.97, 6.22)	6.14 (6.02, 6.26)	0.05 (-0.07, 0.16)	0.04	0.447	6.08 (5.95, 6.21)	6.52 (6.41, 6.62)	0.44 (0.30, 0.57)	0.33	<0.001	<0.001
Question k*	5.29 (5.12, 5.46)	5.36 (5.19, 5.53)	0.08 (-0.09, 0.24)	0.05	0.407	5.24 (5.07, 5.41)	6.20 (6.05, 6.34)	0.95 (0.76, 1.14)	0.51	<0.001	<0.001
Question l*	5.24 (5.07, 5.41)	5.52 (5.35, 5.68)	0.28 (0.10, 0.45)	0.16	0.002	5.26 (5.10, 5.43)	6.04 (5.89, 6.19)	0.78 (0.60, 0.95)	0.45	<0.001	<0.001
Question m*	5.67 (5.53, 5.82)	5.86 (5.72, 6.0)	0.19 (0.04, 0.33)	0.13	0.014	5.89 (5.76, 6.02)	6.37 (6.26, 6.48)	0.48 (0.33, 0.63)	0.33	<0.001	0.006

* Paired t-tests and Two-sample t-test for change from Pre-test to Re-test or Post-test (Re-Pre, Post-Pre): Mean (95% CI), Cohen’s d effect sizeAttitude Range: 1 = more negative response to 7 = more positive response. Effect size: 0.2 = small, 0.5 = medium, 0.8 = larg

### Outcomes

#### Knowledge

Post-test results showed that after experiencing *iLookOut*, the total knowledge score in the intervention group increased from 13.54 to 16.19 out of 21 (95% CI; large effect size of 0.95, *p <* 0.001), whereas the control group’s total score was almost identical (95% CI; 13.54 to 13.59, effect size 0.03, *p =* 0.684) (Table 2). In the intervention group, one knowledge item (3c) underwent highly marked change (large effect size 0.87, *p <* 0.001), 14 items changed with medium effect sizes ranging from 0.22 to 0.77 (items 1d, 1e, 1f, 1g, 2a, 2b, 2c, 2e, 3a, 3b, 5, 6, 7, 9; all *p <* 0.001), and six items changed with small effect sizes (items 1a, 1b, 1c, 2d, 4, 8; all *p <* 0.001) (Table 2). For the control group, results for all items were highly stable and no individual items underwent positive change with an effect size greater than 0.22 (Table 2).

#### Attitudes

Post-test results showed that after experiencing *iLookOut*, attitudes in the intervention group underwent marked change. All 13 items changed positively with medium effect sizes ranging from 0.22 to 0.52 (all *p <* 0.001) (Table 3), with three items (h, i, k) changing with effect sizes exceeding 0.5. Contextually, this group’s attitudes regarding seven of the 13 items changed positively by at least 0.5 of a point on the Likert scale. By comparison, the control group’s attitudes remained constant, with only two items (h, i) experiencing change with medium effect sizes of 0.21 (*p <* 0.001) (Table 3), and contextually the control group’s attitudes did not change for any item by more than 0.32 of a point on the Likert scale.

#### Follow-up after 4 months post-intervention

On re-contact for follow-up testing after four months, there were 201 participants (43.69%), all of whom had received the intervention and whose knowledge and attitude had been measured after it during the initial 4-week study. The follow-up of these participants demonstrated a sustained significant increase in mean knowledge from baseline of 13.54 to 15.16 (Table 4). However, the mean follow-up knowledge score for these 201 CCPs declined to 15.16 (95% CI = 14.87, 15.45) from its post-test peak of 16.86 (*p <* 0.001, 95% CI = 16.60, 17.12). Three knowledge items showed notable declines of > 15% (items 1g, 6, 8).

**Table 4:** Post-intervention to 4 month follow-up–knowledge.

Variable	Post-intervention (N = 201)	Follow-up after 4 months (N = 201)	Change	Effect Size	P-value
Total score*	16.86 (16.60, 17.12)	15.16 (14.87, 15.45)	-1.70 (-2.01, -1.38)	0.75	<0.001
Question 1a^†^	97.5	94.0	-3.5	0.18	0.118
Question 1b^†^	96.0	93.5	-2.5	0.11	0.302
Question 1c^†^	11.4	10.4	-1.0	0.03	0.845
Question 1d^†^	99.5	94.0	-5.5	0.35	0.003
Question 1e^†^	100.0	94.5	-5.5	0.47	<0.001
Question 1f^†^	100.0	97.0	-3.0	0.35	0.031
Question 1g^†^	28.9	12.9	-16.0	0.40	<0.001
Question 2a^†^	100.0	95.0	-5.0	0.45	0.002
Question 2b^†^	65.7	75.6	9.9	0.22	0.009
Question 2c^†^	94.5	93.0	-1.5	0.06	0.664
Question 2d^†^	68.2	73.1	4.9	0.11	0.149
Question 2e^†^	95.5	86.6	-8.9	0.32	0.003
Question 3a^†^	90.5	79.1	-11.4	0.32	<0.001
Question 3b^†^	96.0	95.5	-0.5	0.02	1.000
Question 3c^†^	90.5	75.6	-14.9	0.41	<0.001
Question 4^†^	48.3	48.8	0.5	0.0	0.909
Question 5^†^	92.0	83.1	-8.9	0.27	0.003
Question 6^†^	75.1	49.8	-25.3	0.53	<0.001
Question 7^†^	93.0	79.1	-13.9	0.41	<0.001
Question 8^†^	43.8	1.0	-42.8	1.25	<0.001
Question 9^†^	99.0	84.1	-14.9	0.62	<0.001

*Paired t-test for Post-test vs. Follow-up: Mean (95% CI).

†McNemar’s test for Post-test vs. Follow-up: % correct

For attitudes, several items remained stable from a very high post-intervention baseline (Table 5). There were some significant negative changes with five of 13 items changing with medium effect sizes over 0.5 and also decreasing by over 0.5 of a point on the Likert scale (items c, g, h, i, k). However, these declines were from a very high base so that even with the downward trend, the attitudes remained strong.

**Table 5:** Post-intervention to 4 month follow-up–attitudes.

Variable	Post-intervention (N = 201)	Follow-up after 4 months (N = 201)	Change	Effect size	P-value
Attitude average	6.54 (6.47, 6.61)	6.10 (5.99, 6.21)	-0.44 (-0.55, -0.33)	0.56	<0.001
Question a*	6.51 (6.35, 6.66)	6.45 (6.32, 6.57)	-0.06 (-0.23, 0.11)	0.05	0.481
Question b*	6.41 (6.26, 6.57)	6.12 (5.96, 6.28)	-0.29 (-0.49, -0.10)	0.21	0.004
Question c*	6.76 (6.67, 6.84)	6.14 (5.94, 6.34)	-0.62 (-0.83, -0.40)	0.40	<0.001
Question d*	6.71 (6.62, 6.81)	6.27 (6.11, 6.44)	-0.44 (-0.63, -0.25)	0.32	<0.001
Question e*	6.64 (6.54, 6.74)	6.61 (6.51, 6.71)	-0.03 (-0.14, 0.07)	0.04	0.519
Question f*	6.63 (6.52, 6.74)	6.23 (6.07, 6.40)	-0.39 (-0.58, -0.20)	0.28	<0.001
Question g*	6.72 (6.62, 6.82)	5.99 (5.74, 6.23)	-0.74 (-0.99, -0.48)	0.40	<0.001
Question h*	6.56 (6.43, 6.68)	6.00 (5.79, 6.22)	-0.55 (-0.80, -0.31)	0.31	<0.001
Question i*	6.44 (6.27, 6.61)	5.70 (5.47, 5.93)	-0.74 (-0.98, -0.50)	0.42	<0.001
Question j*	6.63 (6.52, 6.73)	6.40 (6.25, 6.56)	-0.22 (-0.38, -0.07)	0.19	0.006
Question k*	6.37 (6.19, 6.55)	5.45 (5.18, 5.71)	-0.93 (-1.24, -0.62)	0.42	<0.001
Question l*	6.09 (5.89, 6.29)	5.72 (5.51, 5.93)	-0.37 (-0.60, -0.14)	0.22	0.002
Question m*	6.55 (6.41, 6.68)	6.23 (6.09, 6.38)	-0.31 (-0.48, -0.15)	0.26	<0.001

*Paired t-test for Post-test vs. Follow-up: Mean (95% CI).

## Discussion

This study found that compared to the control group, participants who received the iLook Out for Child Abuse (*iLookOut*) educational intervention displayed significantly higher overall knowledge of the duty to report child abuse and neglect, and significantly higher knowledge on a wide range of individual knowledge items. In addition, the intervention group’s attitudes showed significant movement in the desired direction on every attitudinal item, compared with the control group. The stability in both knowledge and attitudes amongst the control group indicates the absence of any “improvement with practice” effect, and indicates the *iLookOut* educational intervention brings about positive change in knowledge and attitudes in this group of professionals.

### Knowledge

This study found that *iLookOut* produced significant improvements in knowledge regarding all aspects of the reporting duty, ranging from the types of injuries required to be reported, protections for reporters, the consequences for failing to report, and the mechanism for reporting. Improved total knowledge was evident, and improvement on 15 out of 21 knowledge items was shown with either large or medium effect size, despite the relatively high baseline knowledge possessed by participants. However, despite these impressive improvements, some knowledge items remained less accurately answered, with participants demonstrating mistaken beliefs that they were required to report any kind of injury caused by a parent, any witnessing of domestic violence, and any bruising in a child aged under five, and a further persistent misapprehension about to whom they should report.

### Attitudes

This study also found that *iLookOut* produced significant improvements in the attitude towards the reporting duty. The intervention group showed improvement on all 13 attitude items, with the most significant changes on three items regarding the role of reporting in promoting children’s long-term interests; the safety of reporters from legal liability if the report is unsubstantiated; and the duty to report applying even if one’s supervisor disagrees. Some of the positive changes in attitude were less expected, as *iLookOut* only indirectly addressed key issues underlying these attitudes. For example, after completing *iLookOut* participants were more likely to hold that child protection services (CPS) would respond effectively to reports, and that children and their families could receive helpful services. Because *iLookOut* did not present detailed data about the efficacy of CPS, this suggests that when CCPs better understand the nature and consequences of child abuse, as well as how to engage CPS, they are more likely to view the system as responsive to the needs of at-risk children and their families.

### Four month follow-up: Sustained gains

The study found that initial positive gains in both knowledge and attitudes were sustained over a four month follow-up period. Knowledge regression occurred significantly on three items, although notably these were poorly answered initially (domestic violence, how soon to report, to whom to report). Regression in knowledge was expected given the normal transience of single dose interventions and the need for longitudinal reinforcement to sustain gains in learning, but the sustained knowledge gains overall indicate the efficacy of *iLookOut* in producing sustained cognitive gain. To address the identified regression, forthcoming versions of *iLookOut* will add further content to clarify areas of confusion, and leverage electronic communication and spaced content retrieval techniques to enhance cognitive mapping. Similarly, attitudes at four month follow-up demonstrated persistent overall gain, with attitudes towards some items being further strengthened, suggesting a deepened attitudinal response over time, fostered by the unusually deep engagement of the intervention with the affective dimension of this context. However, attitudes towards some items regressed, perhaps indicating a growing understanding of the complexity of some aspects of this context. For example, subsequent decreased desire to fulfil professional responsibility by reporting may be explicated by a growing understanding that some meritorious reports do not yield positive outcomes for families.

To address this, forthcoming versions of the intervention will explore such possibilities. However, other attitudinal regression may be due to contamination of attitudes by incorrect knowledge. For example, participants were less committed than they had previously been about whether they should be required by law to report child maltreatment. We would hypothesise this regression is linked with the identified knowledge gaps; this less positive attitude could be explained by an incorrect belief that the law requires all cases of exposure to domestic violence to be reported. To address this, future versions of *iLookOut* will add further content to clarify areas of confusion in knowledge, and create further connections between the development of knowledge and attitudes to create a coherent cognitive and affective framework.

### Contributions to knowledge

To our knowledge, relative to other studies, the *iLookOut* intervention is the first randomized controlled trial with CCPs to explore changes in knowledge of the duty to report all kinds of child maltreatment, and attitudes towards the duty. Other types of study have shown increased knowledge, including one study which showed a significant difference between pretest and post-test scores (15.3 to 17.8 out of 20) [31]. However, there are few if any comparable studies with a large sample, control and intervention groups, carefully designed scales, application to multiple forms of maltreatment, and with a focus on attitudinal development. The systematic review of training and procedural interventions to improve child protection [34] identified only three studies using a control group and outcome measures of one or more of learning achievement, attitudinal change, and clinical behaviour [29] [40] [41]. Even these three studies were limited, either assessing knowledge only of sexual abuse [29] [41], or relying only on self-reported change in knowledge rather than objective measures [29] [40]. Furthermore, none of these studies were conducted with CCPs, which are a group of professionals who have rarely received educational efforts that have been rigorously designed and evaluated, with two exceptions [33] [35]. Since the systematic review [34], only two controlled trials have been conducted and each had a different focus to *iLookOut*. One was a small study of nurses’ clinical capacity to detect cases [32], and the other was devoted to exploring knowledge of child sexual abuse and attitudes towards it [33], with this program being further explored for effects on reporting of child sexual abuse [42].

Overall, the research implications are that a customized, multimethod educational intervention delivered online to a large group of CCPs can significantly increase knowledge and change attitudes about the duty to report suspected child maltreatment, with these increases able to be largely sustained over time. While the precise mechanism of change in developing knowledge and attitudes has not been isolated in this context [43], these findings can be plausibly explained as an exemplar of adult learning [44]. Child protection training for professionals is an educational intervention through which professionals develop cognitive capacity, building knowledge and skills, as well as attitudes. While previous studies have focused on the cognitive domain, the *iLookOut* intervention was designed to include measures to enhance empathy towards maltreated children as a means of fostering positive attitudes towards the duty to report suspected cases. Empathy is known to encourage prosocial altruistic behaviour, and can be fostered through education [45] [46]. Combined with greater cognitive understanding of the nature and function of child protection systems, this additional attention to the affective domain may have acted as a mechanism to foster more positive attitudes towards the duty to report. Further research into the empathic domain would be beneficial, especially to investigate areas known to be more resistant to empathic development and response [45]. As done to an extent in some other studies [40] [42], research to explore the effect of increased knowledge and attitudes on actual reporting practice would be beneficial.

Clinically and practically, the significance of these findings derives from the low rate of reports made by CCPs, and the need for tools to overcome barriers to preparing and motivating CCPs to identify and report child maltreatment. There is a need for robust education of CCPs, and other professionals, using proven methodologies. Studies of other professionals indicate reports of maltreatment are more likely when the reporter has higher knowledge of indicators of maltreatment [47], and higher knowledge of the reporting law and procedures [22] [25] [48]. In addition, as suggested elsewhere [49], this study confirmed the feasibility of administering an online, interactive educational intervention without financial compensation for a large cohort of professionals with heavy workloads and competing time demands.

#### Generalisability

This randomized controlled trial was conducted with a representative sample across demographic strata, with slight overrepresentation of Non-Hispanic White and slight underrepresentation of non-Hispanic Black participants. While the profession studied is inherently almost exclusively female, there is currently no proven influence of gender on knowledge or attitudes in this context, and overall findings indicate beneficial outcomes from this educational intervention can be expected across a range of participants.

The trial was conducted with one group of professionals (CCPs). Given that a range of professions may have different baseline knowledge and attitudes, findings may vary across professional groups, and possibly across genders. However, nonrandomized studies with other professions across genders have shown increases are possible [31]. Additionally, given the high baseline knowledge in our sample, likely influenced by high exposure to prior education about child maltreatment (78.4%), knowledge gains amongst other groups with less prior education could be higher than demonstrated in this study.

Similarly, this study was conducted in one state of the U.S. (Pennsylvania). While further research at statewide level elsewhere would need to confirm generalizability, this is the largest statewide study conducted in the U.S. and featured a representative sample, suggesting applicability of outcomes more generally. In other jurisdictions, CCPs and other professions may have lower baseline knowledge and different baseline attitudes. This is particularly so, given the likely increase in general public awareness of child sexual abuse after the extremely high profile Sandusky scandal in Pennsylvania, with attendant media attention and subsequent legislative changes, all of which occurred in the two year period preceding this study [39]. This may have produced a sample possessing high baseline knowledge and pro-active attitudes relative to other jurisdictions, placing a low ceiling on potential gains from any intervention. The results of this study suggest that in jurisdictions with similar duties to report child maltreatment, and similar child protection systems, this intervention is likely to also improve knowledge and attitudes, possibly with even more significant change.

#### Limitations

The study had several limitations. First, despite being one of the few randomized controlled trials to be conducted in this context, even an RCT remains susceptible to contamination of internal validity by maturation and history, and threatened external validity by the interaction of pretesting and treatment [50]. While a randomized Solomon four group design can overcome these threats [51], we chose not to adopt this approach due to the need for a much larger sample. Nevertheless, we reduced the impact of maturation and history by ensuring a short period of time between pretest and post-test, and external validity did not appear affected based on the stability of the control group’s outcomes. Second, the design meant participation was limited to CCPs with internet access at home or at work. While online education is increasingly commonplace, future studies could nevertheless ensure involvement of participants by providing internet access for those who do not have it. Third, the sample included few home-based CCPs, and this subset of participants needs to be further explored in future research. Fourth, the four month follow-up group was smaller than we had hoped. However, the 43.69% response rate among those who were re-contacted is comparable to other internet-based research [52], and these participants were demographically similar to those who declined. Finally, the study involved a wide range of participating facilities, and therefore involved clusters of individual CCPs. While it is possible and even likely that participants had received some child abuse training, this would likely be the same kind of training across the entire cohort since such training is state-mandated and must be approved at the state level. Accordingly, it is unlikely that groups of participants had unique or different training content provided by their center, or that participants from the same facilities had correlated scores because of such training. Future research could further consider this possibility.

## Conclusion

The findings of this study support efforts to deliver customized multidisciplinary education to professionals as part of society’s broader response to child maltreatment. Childcare professionals who experienced *iLookOut*, a customized online educational program designed to improve knowledge of the legal duty to report child abuse and neglect, and of attitudes towards the duty, demonstrated substantially improved outcomes in both knowledge and attitudes. These gains were largely sustained at four month follow-up, although some slight decline indicates further gains may be produced by repeated iterations focused on areas of confusion and conceptual and practical difficulty. The study offers support for further investment in online educational programs and inclusion of components aimed at developing participants’ cognitive and affective attributes.

## Supporting information

S1 Fig CONSORT 2010 checklist. (PDF)

S1 File Study protocol. (PDF)

S2 File Knowledge and attitudes scales. (PDF)

S3 File Data repository–demographics. (PDF)

S4 File Data repository–knowledge. (PDF)

S5 File Data repository–attitudes. (PDF)
