# Supplementary material for: Educating early childhood care and education providers to improve knowledge and attitudes about reporting child maltreatment: A randomized controlled trial
Source: PLoS One. 2017 May 19;12(5):e0177777. doi: 10.1371/journal.pone.0177777 (PMC5438118; doi:10.1371/journal.pone.0177777)
Supplement: S1 File — (PDF) [file pone.0177777.s002.pdf]

PENNSTATE

Protocol for
Human Subject Research

Protocol Title:
Early Childhood practitioner's knowledge and attitudes regarding reporting child abuse/neglect: iLookOut

Principal Investigator:
Benjamin H Levi, MD, PhD
Department of Humanities/Pediatrics
blevi@hmc.psu.edu
717 531-5158

Version Date:
January 26, 2014

Clinicaltrials.gov Registration #:
NCT02225301

If you need help…

University Park and other campuses:	College of Medicine and Hershey Medical Center:
Office for Research Protections Human Research Protection Program<br>The 330 Building, Suite 205<br>University Park, PA 16802-7014<br>Phone: 814-865-1775<br>Fax: 814-863-8699<br>Email: irb-orp@psu.edu	Human Subjects Protection Office<br>90 Hope Drive, Mail Code A115, P.O. Box 855<br>Hershey, PA 17033<br>(Physical Office Location: Academic Support Building Room 1140)<br>Phone: 717-531-5687<br>Fax number: 717-531-3937<br>Email: irb-hspo@psu.edu

Instructions for using this protocol template:
- Add this completed protocol template to your study in CATS IRB (http://irb.psu.edu) in the "Basic Information" section. Links to Penn State's protocol templates are available in the same location where they are uploaded and their use is required.
- This template is provided to help investigators prepare a protocol that includes the necessary information needed by the IRB to assess risks to human subjects in research.
- There may be sections in this template that do not apply. If so, provide the response "Not Applicable".
- All guidance language appears in ***red italics*** and should be deleted from the final version of the protocol.

Table of Contents

1.0 Objectives 2
2.0 Background 2
3.0 Inclusion and Exclusion Criteria 3
4.0 Recruitment Methods 3
5.0 Consent Process and Documentation 4
6.0 Study Design and Procedures 5
7.0 Data and Specimen Banking 6
8.0 Statistical Plan 6
9.0 Confidentiality, Privacy and Data Management 7
10.0 Data and Safety Monitoring Plan 7
11.0 Risks 8
12.0 Potential Benefits to Subjects and Others 8
13.0 Sharing Results with Subjects 8
14.0 Economic Burden to Subjects 8
15.0 Number of Subjects 8
16.0 Resources Available 9
17.0 Other Approvals 9
18.0 Subject Stipend and/or Travel Reimbursements 9
19.0 Multi-Site Research 9
20.0 Adverse Event Reporting 9
21.0 Study Monitoring, Auditing and Inspecting 10
22.0 References 10
23.0 Appendix 10

1.0 Objectives

1.1 Study Objectives

(1)To evaluate the effectiveness of the eLearning Module (iLook Out for Child Abuse) created to train and inform early childhood practitioners (ECPs);

(2)To identify ECPs' current knowledge regarding Pennsylvania law regarding mandated reporting of suspected child abuse;

(3)To explore the factors influencing ECPs' current knowledge and attitudes;

(4)To evaluate the impact of an online educational program on ECPs' knowledge, attitudes, and patterns of decision-making regarding mandated reporting of suspected child abuse;

(5)To explore the factors influencing the impact of an online educational program on ECPs' knowledge and attitudes regarding mandated reporting of suspected child abuse

1.2 Primary Study Endpoints

Recognition and reporting of 'reasonable suspicion' of Child Abuse

1.3 Secondary Study Endpoints

NA

2.0 Background

2.1 Scientific Background and Gaps

Despite their daily interactions with young children, little research has been conducted into early childhood practitioners' (ECPs') knowledge of, and attitudes towards, reporting of child abuse/neglect. A

confluence of circumstances presently offers a rare opportunity to conduct innovative, rigorous and socially meaningful research with a large sample of these professionals. These circumstances include the current social and legal milieu in Pennsylvania, the establishment of the Center for the Protection of Children (CPC) at Penn State and a new initiative within the CPC to develop and disseminate an online educational program for early childhood practitioners throughout Pennsylvania.

2.2 Previous Data

A field study was completed with 60 Penn State Harrisburg undergraduate students majoring in early childhood education to pilot a draft of the present study's pre-/post-assessment instrument.

A subset of the pre-/post-assessment's knowledge items were subsequently pilot-tested with ECPs actively employed at area childcare facilities.

2.3 Study Rationale

The purpose of this study is to explore ECPs' knowledge, attitudes, and patterns of decision-making regarding reporting suspected child abuse.

3.0 Inclusion and Exclusion Criteria

3.1 Inclusion Criteria

Early Childhood Practitioners (ECPs) ≥18 years of age –where "ECP" refers to the various childcare professionals and others who work with young children, including teachers, aides, therapists, administrators, support staff, volunteers, and others.

3.2 Exclusion Criteria

<18 years of age.

3.3 Early Withdrawal of Subjects

3.3.1 Criteria for removal from study

Participants may withdraw from the study at any time.

3.3.2 Follow-up for withdrawn subjects

There will be no follow-up with withdrawn subjects.

4.0 Recruitment Methods

4.1 Identification of subjects

There are two phases to this investigation, the first a randomized controlled trial, the other non-randomized. For the randomized controlled trial, potential participants will be identified using listings from organizations that provide education and other professional services to ECPs (such as Better Kid Care, Capital Area Early Childhood Training Institute, and others). The first 12 participants will be used as a pilot to test the function of the web tool. The data of those pilot testers may or may not be included in the analyses depending on the tool.

For the subsequent (non-randomized) phase, enrollment will be open to any and all ECPs in Pennsylvania.

4.2 Recruitment process

For the <u>randomized controlled trial</u>, a letter and/or email (attached) will be sent to childcare facility directors explaining that we are seeking participants to help us evaluate the impact of *iLook Out for Child Abuse* on ECPs' knowledge and attitudes about reporting suspected child abuse. Directors who are interested will receive a subsequent email or letter with instructions for how they and their staff can learn

more about the specifics of the study, and choose whether they wish to participate. Each individual staff member will be asked for their consent prior to any study activities. We will also contact directors by phone to follow-up and answer any questions they may have.

For the subsequent open research phase, informational fliers and general announcements will be sent (by USPS and email) to childcare facilities and posted on list-serves and other websites frequented by ECPs, describing the content and purpose of *iLook Out for Child Abuse*, and requesting their participation.

4.3 Recruitment materials

For the randomized controlled trial, a letter and/or email (attached) will be sent to childcare facility directors explaining that we are seeking participants to help us evaluate the impact of *iLook Out for Child Abuse* on ECPs' knowledge and attitudes about reporting suspected child abuse. Directors who are interested will receive a subsequent email or letter with instructions for how they and their staff can learn more about the specifics of the study, and choose whether they wish to participate. Each individual staff member will be asked for their consent prior to any study activities. We will also contact directors by phone to follow-up and answer any questions they may have.

For the subsequent open research phase, informational fliers and general announcements will be sent (by USPS and email) to childcare facilities and posted on list-serves and other websites frequented by ECPs, describing the content and purpose of *iLook Out for Child Abuse*, and requesting their participation.

4.4 Eligibility/screening of subjects

NA

5.0 Consent Process and Documentation

5.1 Consent Process

5.1.1 Obtaining Informed Consent

5.1.1.1 Timing and Location of Consent

Upon accessing the website for *iLook Out for Child Abuse*, individuals will be provided a summary explanation of the research, and informed that choosing to proceed with the learning module constitutes consent for their data to be collected. Individuals not wishing to participate need not proceed to the learning module, and it will be made clear that participants may stop at any time.

5.1.1.2 Coercion or Undue Influence during Consent

Because potential participants can choose to proceed (or not proceed) with the learning module without the researchers ever knowing their identity, there is no opportunity for research to exert undue influence for their participation.

5.1.2 Waiver or alteration of the informed consent requirement

NA

5.2 Consent Documentation

5.2.1 Written Documentation of Consent

Participants consent will be implicit in their choice to proceed with the online learning module.

5.2.2 Waiver of Documentation of Consent

NA

5.3 Consent – Other Considerations

5.3.1 Non-English Speaking Subjects

The online learning module was designed only for individuals who speak English.

5.3.2 Cognitively Impaired Adults

5.3.2.1 Capability of Providing Consent

Only those individuals capable of understanding the instructions will proceed, which itself will provide evidence of the capacity to provide informed consent.

5.3.2.2 Adults Unable To Consent

NA

5.3.2.3 Assent

NA

5.3.3 Subjects who are not yet adults (infants, children, teenagers)

5.3.3.1 Parental Permission

NA

5.3.3.2 Assent

NA

6.0 Study Design and Procedures

6.1 Study Design

All components of the learning module being studied are online, and will be accessed by participants through a secure website. As such, participants will complete their registration online (including collection of demographic data and creation of a username and password), after which they will be sent an email with a secure link for activating their personal account.

In the randomized controlled phase of this study, participants will be randomized to either the control arm or the intervention arm. The only difference between these arms is that control arm participants will complete two (2) post-assessments instead of one, the purpose of which is to control for test/re-test improvement. Consequently, all participants will be asked to complete the multiple-choice pre-assessment, the learning module, the post-assessment, and an evaluation of the learning module. However, participants in the control arm will be asked to complete the post-assessment directly following the pre-assessment (i.e., in addition to completing it after the learning module).

Participants who agree to be contacted several months later will also be asked to complete the post-assessment one final time and a few additional evaluation questions, the purpose being to assess long-term impact of the intervention on knowledge and attitudes.

Because we hope to geographically cluster the recruitment of participants, the researchers will also examine whether the reporting rates of suspected abuse for a given county change from their baseline following completion of the learning module. Because these reporting rates can only be examined at the county level, and information about them is both anonymous and aggregate only, there is no potential for linking the identities of study participants with individuals who have made reports of suspected abuse.

6.2 Study Procedures

Following registration and collection of demographic data, participants will activate their online account through the link provided to them via email. Participants will then be asked to complete the pre-

assessment, followed by the post-assessment if they are in the control arm. All participants will then be asked to complete the learning module, followed by the post-assessment, followed by an evaluation of the learning module. Those participants who consent to be contacted later will also be asked several months later to repeat the post-assessment one final time, as well as answer questions about their impressions of the learning module and its impact.

The randomized controlled trial phase of this research project is expected to last ~3 months in order to enroll 300-500 individuals, who will be divided into two groups –whose only difference will be the repeat administration of the post-test for the control arm group.

Following the close of the randomized controlled trial phase, data will be analyzed, and edits will be made to the *iLook Out for Child Abuse* learning module if it appears that (based on the data) there are areas that yield less effective learning.

The follow-up to phase I of the *iLook Out for Child Abuse* learning module will occur over approximately 2-4 months. The Center for the Application of Information and Technologies, who is storing the research data, will send the research team email, name, and participant ID number for only the participants that have agreed to long-term follow-up. This information will be transferred through secure files, and it will not be attached to any research data at the time of transfer. The Penn State Hershey research staff will replace the existing research number with a new participant ID prior to sending out follow-up surveys.

Participants who have agreed to follow-up will receive a separate email with a new summary explanation of research and email message stating the purposes for our contact. The new summary explanation will clearly explain the use of REDCap in the follow-up portion of the study. If a participant responds to the message and requests to be taken off of our follow-up list, the participant will be taken off our list. All other participants will be sent a survey invitation through REDCap. The reminder email attached to this submission will be sent out to participants who have not completed the survey on approximately a bi-weekly or weekly basis.

The study PI would like to directly email the follow-up participants who have not completed the survey during the last week the survey is open. This will allow the study team to make one last request for participation and thank individuals for participation in the *iLookOut for Child Abuse* learning module and follow-up survey. This final email message will be paired with a final REDCap survey request, and the email text is included in this submission.

Prior to formal recruitment of participants for the randomized controlled trial phase, area ECPs known to the researchers will be asked to test-run the registration, assessments, and learning module for the purposes of providing final feedback on language clarity, ease of use, etc., so that any last-minute adjustments can be made before enrolling participants.

After the participant completes the *iLook Out for Child Abuse* learning module, Center for the Application of Information and Technologies, who is storing the research data, will provide the researchers listed on this application the names of the participants that completed the module. This is necessary to register participants for official credit with PA Keys, which is a participant benefit for participating in this training.

6.3 Duration of Participation

Individuals who choose to participate in the study may choose to complete the study in one sitting, or may log out of the learning module and return at a later time. It is anticipated that will take individuals 5-10 minutes to complete registration and provide demographic data; 5-10 minutes to complete the pre-assessment; 60-90 minutes to complete the *iLook Out for Child Abuse* learning module; 5-10 minutes to complete the post-assessment; and 5 minutes to complete the evaluation of the learning module. Upon successful completion of the learning module, assessments, and evaluation, participants will be able to

print and save a certificate documenting two hours of approved credit for Pennsylvania state required training on mandated reporting.

Participants who agree to long-term follow-up will be contacted again approximately four months after completion of the original learning module. The follow-up survey will take participants approximately 20 minutes to complete. After the follow-up survey is complete, the participant's participation in this study will be complete.

7.0 Data and Specimen Banking

7.1 Data and/or specimens being stored
Information collected will include demographic information, as well as responses to the pre-/post-assessments, learning module, and program evaluation.

7.2 Location of storage
Data will be collected online, and the study coordinated out of the Penn State Hershey Center for the Protection of Children on the campus of the Milton S Hershey Medical Center, College of Medicine. Data will be stored on the servers of the Center for the Application of Information and Technologies at Western Illinois University, with de-identified data available to study team members.

7.3 Duration of storage
Data will be stored until the research is completed.

7.4 Access to data and/or specimens
Study team members will have full access to de-identified data. Center for the Application of Information and Technologies staff will be able to access full information –which might be needed if a participant was having trouble accessing their account, and needed proof of their having completed their training.

For some complex analyses, de-identified (non-PHI) data may be shared with consultants outside the research team.

7.5 Procedures to release data or specimens
From the SQL database that initially collects that data, an Excel-type file will be exported from The Center for the Application of Information and Technologies, which is building and will manage the website through which this learning module will be administered. The site is a role-based privilege system. Only users who will have the admin roles will be able to view the results of the assessments.

7.6 Process for returning results
NA

8.0 Statistical Plan

8.1 Sample size determination
Based on the statistical results from the IRB study # 43589 a study group of 300 is required for appropriate baseline data for the randomized controlled trial phase of this study. Because the second phase of the study will be open enrollment, no limit is projected.

8.2 Statistical methods
Dr. Chengwu Yang and Erik Lehman from the Penn State Hershey Public Health Department will use standard statistical methods to analyze the results.

9.0 Confidentiality, Privacy and Data Management

9.1 Confidentiality

9.1.1 Identifiers associated with data and/or specimens

Data will be stored electronically on password-protected, secure servers at the Center for the Application of Information Technologies which is also hosting the learning module and assessments. The site has role based privileges.

Data and participant information stored at the Penn State Hershey Medical Center will be stored in secure electronic and paper files.

9.1.1.1 Use of Codes, Master List

NA

9.1.2 Storage of Data and/or Specimens

Data will be stored electronically on password-protected, secure servers at the Center for the Application of Information Technologies which is also hosting the learning module and assessments. The site has role based privileges.

Data and participant information stored at the Penn State Hershey Medical Center will be stored in secure electronic and paper files.

9.1.3 Access to Data and/or Specimens

Only members of the research team and project members at the Center for the Application of Information Technologies will have access to the data. The primary data storage site is role-based with administrative privileges for viewing the results of the assessments. All data will be stored securely and accessible only to research personnel listed on this application and authorized employees of the Center for the Application of Information Technologies.

9.1.4 Transferring Data and/or Specimens

Data will be stored and formatted in a MYSQL database and then transferred into an Excel spreadsheet. Data transferred from the Center for the Application of Information Technologies to the study team will primarily include de-identified data. Identifiable information such as participant name may be shared with researchers listed on this application for the purposes of registering participants for PA Keys credit. For data from the first part of Phase I, these identifiers will not be connected to study data.

9.2 Privacy

Data will be de-identified for the purposes of research. To allow for verification that a participant did in fact successfully complete the learning module (and hence should receive certification of this), the data stored at the Center for the Application of Information Technologies will link individual names with post-assessment results, and will link the names to email addresses for the purposes of sending follow-up questions to participants who agree to be contacted several months after completing the learning module.

The Center for the Application of Information Technologies will transfer email, participant name, and study code number only for the participants that have agreed to long term follow-up. This allows us to conduct the follow-up portion of the study through REDCap, a secure data management program.

10.0 Data and Safety Monitoring Plan

10.1 Periodic evaluation of data

NA

10.2 Data that are reviewed

NA

10.3 Method of collection of safety information

NA

10.4 Frequency of data collection

Data will be collected from participants as they progress through the assessment tools.

10.5 Individual's reviewing the data

NA

10.6 Frequency of review of cumulative data

NA

10.7 Statistical tests

NA

10.8 Suspension of research

NA

11.0 Risks

There is a risk of loss of confidentiality participant information or identity is obtained by someone other than the investigators, but precautions will be taken to prevent this from happening.

The study has no risks for participants beyond those encountered in everyday life.

12.0 Potential Benefits to Subjects and Others

12.1 Potential Benefits to Subjects

Successfully completing the training module that is part of this research study will provide participants with education about child abuse and their responsibilities as mandated reporters, as well as a certificate noting two hours of approved training on Mandated Reporting through PA KEYS. There are no financial or other direct benefits from participation.

12.2 Potential Benefits to Others

To the extent that the learning module is effective in improving participants' awareness and knowledge, they will be better prepared to take the appropriate steps to identify and report children at risk for child abuse –which would benefit society as a whole.

13.0 Sharing Results with Subjects

Upon successful completion of the learning module, participants would learn the results of their responses on the post-assessment tool. We anticipate that aggregate results will be published, and generally available to the public.

14.0 Economic Burden to Subjects

14.1 Costs

There will not be a cost associated with participation in the study.

14.2 Compensation for research-related injury

NA

It is the policy of the institution to provide neither financial compensation nor free medical treatment for research-related injury. In the event of injury resulting from this research, medical treatment is available

but will be provided at the usual charge. Costs for the treatment of research-related injuries will be charged to subjects or their insurance carriers.
It is the policy of the institution to provide neither financial compensation nor free medical treatment for research-related injury. In the event of injury resulting from this research, medical treatment is available but will be provided at the usual charge. Such charges may be paid by the study sponsor as outlined in the research agreement and explained in the consent form.

15.0 Number of Subjects

The number of participants for the randomized controlled trial phase will need to enroll 300-500, based on statistical analysis and Power Study of IRB # 43589. However, the design of the study does not allow us to control the number of participants that respond to the elearning invitation or the number of staff members child care centers may provide access to the link. If centers show interest in the learning module, we could receive a maximum of 1,500 participants.

16.0 Resources Available

16.1 Facilities and locations

Recruitment will occur via email, list-serve postings, and fliers, with participants accessing the learning module online at locations convenient to them.

16.2 Feasibility of recruiting the required number of subjects

We are working with several organizations that will help us recruit participants (e.g., Better Kid Care, Capital Area Early Childhood Training Institute, and Pennsylvania Child Care Association) by providing information to their members and other affiliates.

16.3 PI Time devoted to conducting the research

Benjamin Levi, MD, PhD will continue to devote the time and attention required for the management of this research project.

16.4 Availability of medical or psychological resources

NA

16.5 Process for informing Study Team

Routine study meetings, emails, and phone calls.

17.0 Other Approvals

NA

18.0 Subject Stipend and/or Travel Reimbursements

NA

19.0 Multi-Site Research

NA

19.1 Communication Plans

NA

19.2 Data Submission and Security Plan

NA

19.3 Subject Enrollment

NA

19.4 Reporting of Adverse Events and New Information

NA

19.5 Audit and Monitoring Plans

NA

20.0 Adverse Event Reporting

20.1 Reporting Adverse Reactions and Unanticipated Problems to the Responsible IRB

In accordance with applicable policies of The Pennsylvania State University Institutional Review Board (IRB), the investigator will report, to the IRB, any observed or reported harm (adverse event) experienced by a subject or other individual, which in the opinion of the investigator is determined to be (1) unexpected; and (2) probably related to the research procedures. Harms (adverse events) will be submitted to the IRB in accordance with the IRB policies and procedures.

20.2 Auditing and Inspecting

The investigator will permit study-related monitoring, audits, and inspections by the Penn State quality assurance program office(s), IRB, the sponsor, and government regulatory bodies, of all study related documents (e.g. source documents, regulatory documents, data collection instruments, study data etc.). The investigator will ensure the capability for inspections of applicable study-related facilities (e.g. pharmacy, diagnostic laboratory, etc.).

21.0 Study Monitoring, Auditing and Inspecting

21.1 Auditing and Inspecting

The investigator will permit study-related monitoring, audits, and inspections by the Penn State quality assurance program office(s), IRB, the sponsor, and government regulatory bodies, of all study related documents (e.g. source documents, regulatory documents, data collection instruments, study data etc.). The investigator will ensure the capability for inspections of applicable study-related facilities (e.g. pharmacy, diagnostic laboratory, etc.).

22.0 References

Levi BH, Boehme S, Dellasega C. *What is Reasonable Suspicion of Child Abuse?* J. Public Child Welfare. 2012;6(5):569-589.

Crowell K, Levi BH. *Mandated Reporting Thresholds For Community Professionals*. Child Welfare. 2012;91(1):35-53.

Levi BH, Crowell K. *Child Abuse Experts Disagree About The Threshold For Mandated Reporting*. Clinical Pediatrics. 2011;50(4):321-9.

Levi BH, Portwood S. *Reasonable Suspicion of Child Abuse: Finding a Common Language*, J. Law and Medicine. 2011;39(1):62-69

23.0 Appendix
