# Supplementary material for: Educating early childhood care and education providers to improve knowledge and attitudes about reporting child maltreatment: A randomized controlled trial
Source: PLoS One. 2017 May 19;12(5):e0177777. doi: 10.1371/journal.pone.0177777 (PMC5438118; doi:10.1371/journal.pone.0177777)
Supplement: S2 File — (PDF) [file pone.0177777.s003.pdf]

S2 File Knowledge and attitudes scales

Knowledge scale

Key
* For each item in Q 1a-1g, 2a-2e, and 3a-3c, participants were given a choice of yes, no, or unsure.
# For Q 4, participants were given one choice from: any of the above, all of the above, or none of the above.
** For Q 5 and Q 9, participants were given one choice from: true, false or unsure. For Q 6 – Q 8 participants chose only one response.

1. Under recently enacted Pennsylvania law, you would be required to report suspected abuse when a parent, caregiver, or other adult does or causes which of the following to a child?*

a) Physically restrain a child by locking them in a closet
b) Place duct tape over a child's mouth as a form of punishment
c) Cause any kind of physical injury
d) Cause substantial pain from disciplining a child
e) Impair physical functioning from disciplining a child
f) Forcefully slap a child under one year of age
g) Expose a child to domestic violence

2. In which of the following scenarios does bruising raise sufficient concern for child abuse that Pennsylvania law requires you to report it?*

a) Any bruising in an infant who hasn't started pulling to stand
b) Any bruising in a child younger than 5 years old
c) Any bruising from spanking
d) Bruising on a toddler's shins
e) Bruising on a toddler's ears

3. Under Pennsylvania law, if you fail to report suspected abuse/neglect, which of the following penalties might you face?*

a) Financial penalty
b) Loss of professional license
c) Incarceration (i.e., prison)

4. Under recently enacted Pennsylvania law, for an act (or failure to act) to count as abuse/neglect, which of the following must be true? #

The act (or failure to act) must be committed intentionally
The act (or failure to act) must be committed knowingly
The act (or failure to act) must be committed recklessly

5. Under Pennsylvania law, you can be held legally liable if you suspect child abuse/neglect and report it, but it turns out to be unfounded.**

6. Under Pennsylvania law, once you have “reasonable cause to suspect” child abuse/neglect, you must report your suspicion to authorities:

Within a day
Within 48 hours
Within 72 hours
Immediately
Within a week
I am unsure

7. Under Pennsylvania law, you must report:

Whenever I think it is possible that a child has been abused/neglected
Only when I am certain that a child has been abused/neglected
Whenever I have reasonable cause to suspect that a child has been abused/neglected
Only if I think it is more likely than not that a child has been abused/neglected
I am unsure

8. According to newly enacted Pennsylvania law, you are required to report suspected child abuse/neglect to:

The police
ChildLine only
My supervisor only
Both ChildLine and my supervisor
I am unsure

9. Under Pennsylvania law, are you required to report suspected child abuse/neglect if a child was put at significant risk for being injured even when no injury or harm actually occurred? **

Attitude scale

a) I might not report child abuse/neglect for fear of backlash from the family or others
b) I might not report child abuse/neglect because it could result in parents doing something bad to their child
c) I would like to fulfill my professional responsibility by reporting cases where I suspect child abuse/neglect
d) Reporting cases of suspected child abuse/neglect improves children’s safety
e) I would not report child abuse/neglect if I thought the child would be removed from his/her family
f) Reporting suspected child abuse/neglect can result in children and families receiving helpful services
g) Early childhood practitioners should be required by law to report suspected child abuse/neglect
h) Children’s long-term interests are promoted by early childhood practitioners reporting suspected child abuse/neglect
i) I am concerned that I could be held legally liable for reporting child abuse/neglect that turns out to be unfounded
j) It is a waste of time to report child abuse/neglect because often no one follows up on the report
k) I should still report suspected child abuse/neglect even if my work supervisor disagrees with me
l) I have little confidence that Children and Youth Services will respond effectively if I report suspected child abuse/neglect
m) I would not report suspected child abuse/neglect because it is too hard to be sure that abuse occurred
