# Supplementary material for: Educating early childhood care and education providers to improve knowledge and attitudes about reporting child maltreatment: A randomized controlled trial
Source: PLoS One. 2017 May 19;12(5):e0177777. doi: 10.1371/journal.pone.0177777 (PMC5438118; doi:10.1371/journal.pone.0177777)
Supplement: S3 File — (PDF) [file pone.0177777.s004.pdf]

Data dictionary

S3 File Data repository - Demographics

**Data Set Name**	WORK.USER	**Observations**	765
**Member Type**	DATA	**Variables**	37
**Engine**	V9	**Indexes**	0
**Created**	08/15/2014 10:53:47	**Observation Length**	296
**Last Modified**	08/15/2014 10:53:47	**Deleted Observations**	0
**Protection**		**Compressed**	NO
**Data Set Type**		**Sorted**	NO
**Label**			
**Data Representation**	WINDOWS_64		
**Encoding**	wlatin1 Western (Windows)		

Alphabetic List of Variables and Attributes					
**#**	**Variable**	**Type**	**Len**	**Format**	**Informat**
**11**	age_category_id	Num	8	AGEID.	BEST32.
**5**	confident_to_recognize_abuse	Num	8	YN.	BEST32.
**2**	contact_by_email	Num	8	YN.	BEST32.
**8**	county_id	Num	8	COUNTYID.	BEST32.
**1**	dim_user_id	Num	8	BEST12.	BEST32.
**14**	education_level_id	Num	8	EDUCATIONLEVELID.	BEST32.
**19**	employment_type_id	Num	8	EMPLOYMENTID.	BEST32.
**10**	gender_id	Num	8	GENDERID.	BEST32.
**28**	has_consern_for_abuse_ear	Num	8	YN.	BEST32.
**24**	has_consern_for_abuse_infant	Num	8	YN.	BEST32.
**29**	has_consern_for_abuse_none	Num	8	YN.	BEST32.
**27**	has_consern_for_abuse_shin	Num	8	YN.	BEST32.
**26**	has_consern_for_abuse_spanking	Num	8	YN.	BEST32.
**25**	has_consern_for_abuse_younger5	Num	8	YN.	BEST32.
**31**	has_ethnicity_african_american	Num	8	YN.	BEST32.
**34**	has_ethnicity_asian	Num	8	YN.	BEST32.
**32**	has_ethnicity_hispanic	Num	8	YN.	BEST32.
**33**	has_ethnicity_native_american	Num	8	YN.	BEST32.
**35**	has_ethnicity_native_hawaiian	Num	8	YN.	BEST32.
**36**	has_ethnicity_other	Num	8	YN.	BEST32.
**30**	has_ethnicity_white	Num	8	YN.	BEST32.
**22**	have_reported_abuse_id	Num	8	ABUSEID.	BEST32.

Data dictionary

Alphabetic List of Variables and Attributes					
**#**	**Variable**	**Type**	**Len**	**Format**	**Informat**
**23**	have_suspected_abuse_id	Num	8	ABUSEID.	BEST32.
**6**	is_supervisor	Num	8	YN.	BEST32.
**16**	job_responsibility_id	Num	8	JOBRESPONSIBILITYID.	BEST32.
**13**	marital_status_id	Num	8	MARITALSTATUSID.	BEST32.
**18**	number_of_children_category_id	Num	8	NUMBEROFCHILDRENID.	BEST32.
**3**	parent	Num	8	YN.	BEST32.
**15**	preparedness_to_report_id	Num	8	PERPAREDNESSTOREPORTID.	BEST32.
**4**	previously_trained	Num	8	YN.	BEST32.
**12**	religion_id	Num	8	RELIGIONID.	BEST32.
**37**	religiousness_type_id	Num	8	RELIGIOUSNESSID.	BEST32.
**7**	research_category_id	Num	8	RESEARCHCATEGORYID.	BEST32.
**9**	work_environment_type_id	Num	8	WORKENVIRONMENTID.	BEST32.
**17**	work_setting_id	Num	8	WORKSETTINGID.	BEST32.
**20**	years_as_practitioner_id	Num	8	YEARSEXPERIENCEID.	BEST32.
**21**	years_at_workplace_id	Num	8	YEARSEXPERIENCEID.	BEST32.

Alphabetic List of Variables and Attributes	
**#**	**Label**
**11**	How many years old are you?
**5**	Do you feel confident in your ability to identify signs of child abuse/neglect?
**2**	Would you be willing to have the research team (from the survey center) contact you by email in a few months to learn more about your views on mandated reporting by asking you some follow-up questions?
**8**	Select the county in which you work:
**1**	User ID
**14**	What is your highest level of education?
**19**	On what basis are you currently employed?
**10**	What is your gender?
**28**	Has concern for abuse for toddler ear
**24**	Has concern for abuse infant
**29**	Has concern for abuse for none
**27**	Has concern for abuse for toddler shin
**26**	Has concern for abuse for spanking
**25**	Has concern for abuse age younger than 5
**31**	Non-Hispanic Black ethnicity
**34**	Asian ethnicity

Data dictionary

Alphabetic List of Variables and Attributes	
#	Label
32	Hispanic ethnicity
33	Native American ethnicity
35	Native Hawaiian ethnicity
36	Other ethnicity
30	Non-Hispanic White ethnicity
22	In your work as an early childhood practitioner, have you ever reported a case of child abuse/neglect?
23	In your work as an early childhood practitioner, have you ever suspected a case of child abuse/neglect and not reported it?
6	In your current role, are you considered to be a supervisor?
16	Which of the following best describes your primary job responsibilities?
13	What is your marital status?
18	How many children are cared for in your childhood work setting?
3	Are you a parent/guardian?
15	How prepared do you feel to report child abuse/neglect should the need arise?
4	Have you ever had any training about child abuse/neglect?
12	What is your religion?
37	How spiritual/religious would you say you are?
7	Study group
9	Select the environment in which you work:
17	Which of the following best describes your childhood work setting?
20	Including this year, how many years have you worked as an early childhood practitioner?
21	Including this year, how many years have you worked at your current workplace?

Summary

Would you be willing to have the research team (from the survey center) contact you by email in a few months to learn more about your views on mandated reporting by asking you some follow-up questions?				
contact_by_email	Frequency	Percent	Cumulative Frequency	Cumulative Percent
no	304	39.74	304	39.74
YES	461	60.26	765	100.00

Are you a parent/guardian?				
parent	Frequency	Percent	Cumulative Frequency	Cumulative Percent
no	303	39.61	303	39.61
YES	462	60.39	765	100.00

Have you ever had any training about child abuse/neglect?				
previously_trained	Frequency	Percent	Cumulative Frequency	Cumulative Percent
no	166	21.70	166	21.70
YES	599	78.30	765	100.00

Do you feel confident in your ability to identify signs of child abuse/neglect?				
confident_to_recognize_abuse	Frequency	Percent	Cumulative Frequency	Cumulative Percent
no	103	13.46	103	13.46
YES	662	86.54	765	100.00

In your current role, are you considered to be a supervisor?				
is_supervisor	Frequency	Percent	Cumulative Frequency	Cumulative Percent
no	507	66.27	507	66.27
YES	258	33.73	765	100.00

Study group				
research_category_id	Frequency	Percent	Cumulative Frequency	Cumulative Percent
Experimental	376	49.15	376	49.15
Control	389	50.85	765	100.00

Summary

Summary

Select the county in which you work:				
**county_id**	**Frequency**	**Percent**	**Cumulative Frequency**	**Cumulative Percent**
**ADAMS**	56	7.32	56	7.32
**ALLEFHENY**	60	7.84	116	15.16
**BEAVER**	21	2.75	137	17.91
**BLAIR**	5	0.65	142	18.56
**BRADFORD**	2	0.26	144	18.82
**BUCKS**	18	2.35	162	21.18
**BUTLER**	7	0.92	169	22.09
**CAMBRIA**	1	0.13	170	22.22
**CARBON**	4	0.52	174	22.75
**CENTRE**	10	1.31	184	24.05
**CHESTER**	25	3.27	209	27.32
**CLINTON**	7	0.92	216	28.24
**COLUMBIA**	3	0.39	219	28.63
**CRAWFORD**	6	0.78	225	29.41
**CUMBERLAND**	26	3.40	251	32.81
**DAUPHIN**	31	4.05	282	36.86
**DELAWARE**	56	7.32	338	44.18
**ELK**	12	1.57	350	45.75
**ERIE**	9	1.18	359	46.93
**FAYETTE**	5	0.65	364	47.58
**FRANKLIN**	9	1.18	373	48.76
**HUNTINGDON**	1	0.13	374	48.89
**LACKAWANNA**	19	2.48	393	51.37
**LANCASTER**	39	5.10	432	56.47
**LEBANON**	5	0.65	437	57.12
**LEHIGH**	6	0.78	443	57.91
**LUZERNE**	76	9.93	519	67.84
**LYCOMING**	16	2.09	535	69.93
**MONTGOMERY**	58	7.58	593	77.52
**MONTOUR**	13	1.70	606	79.22
**NORTHUMBERLAND**	1	0.13	607	79.35
**PERRY**	18	2.35	625	81.70
**PHILIDELPHIA**	23	3.01	648	84.71

Summary

Select the county in which you work:				
**county_id**	**Frequency**	**Percent**	**Cumulative Frequency**	**Cumulative Percent**
**WASHINGTON**	28	3.66	676	88.37
**WAYNE**	3	0.39	679	88.76
**WESTMORELAND**	23	3.01	702	91.76
**WYOMING**	1	0.13	703	91.90
**YORK**	62	8.10	765	100.00

Select the environment in which you work:				
**work_environment_type_id**	**Frequency**	**Percent**	**Cumulative Frequency**	**Cumulative Percent**
**Rural**	213	27.84	213	27.84
**Suburban**	384	50.20	597	78.04
**Urban**	168	21.96	765	100.00

What is your gender?				
**gender_id**	**Frequency**	**Percent**	**Cumulative Frequency**	**Cumulative Percent**
**Male**	18	2.35	18	2.35
**Female**	747	97.65	765	100.00

How many years old are you?				
**age_category_id**	**Frequency**	**Percent**	**Cumulative Frequency**	**Cumulative Percent**
**<18**	3	0.39	3	0.39
**18-29**	307	40.13	310	40.52
**30-44**	224	29.28	534	69.80
**45+**	231	30.20	765	100.00

What is your religion?				
**religion_id**	**Frequency**	**Percent**	**Cumulative Frequency**	**Cumulative Percent**
**Baptist**	1	0.13	1	0.13
**Jewish**	1	0.13	2	0.26
**Not provided**	763	99.74	765	100.00

Summary

How spiritual/religious would you say you are?				
**religiousness_type_id**	**Frequency**	**Percent**	**Cumulative Frequency**	**Cumulative Percent**
**EXTREMELY_NOT_RELIGIOUS**	10	1.31	10	1.31
**NOT_RELIGIOUS**	56	7.32	66	8.63
**SOMEWHAT_NOT_RELIGIOUS**	15	1.96	81	10.59
**NUETRAL**	122	15.95	203	26.54
**SOMEWHAT_RELIGIOUS**	223	29.15	426	55.69
**RELIGIOUS**	292	38.17	718	93.86
**EXTREMELY_RELIGIOUS**	47	6.14	765	100.00

What is your marital status?				
**marital_status_id**	**Frequency**	**Percent**	**Cumulative Frequency**	**Cumulative Percent**
**Single**	292	38.17	292	38.17
**Married**	408	53.33	700	91.50
**Separated**	52	6.80	752	98.30
**Widowed**	13	1.70	765	100.00

What is your highest level of education?				
**education_level_id**	**Frequency**	**Percent**	**Cumulative Frequency**	**Cumulative Percent**
**8th grade**	1	0.13	1	0.13
**High school**	202	26.41	203	26.54
**CDA**	101	13.20	304	39.74
**Associates**	152	19.87	456	59.61
**Bachelors**	239	31.24	695	90.85
**Masters**	70	9.15	765	100.00

Summary

How prepared do you feel to report child abuse/neglect should the need arise?				
preparedness_to_report_id	Frequency	Percent	Cumulative Frequency	Cumulative Percent
Entirely unprepared	9	1.18	9	1.18
Unprepared	17	2.22	26	3.40
Somewhat unprepared	47	6.14	73	9.54
Neither	47	6.14	120	15.69
Somewhat prepared	265	34.64	385	50.33
Prepared	288	37.65	673	87.97
Very prepared	92	12.03	765	100.00

Which of the following best describes your primary job responsibilities?				
job_responsibility_id	Frequency	Percent	Cumulative Frequency	Cumulative Percent
Pre-school teacher	548	71.63	548	71.63
Early intervention specialist	2	0.26	550	71.90
Kindergarten teacher	6	0.78	556	72.68
Grades 1-4 teacher	14	1.83	570	74.51
Support staff	25	3.27	595	77.78
Assistant director	21	2.75	616	80.52
Director	83	10.85	699	91.37
Other	66	8.63	765	100.00

Which of the following best describes your childhood work setting?				
work_setting_id	Frequency	Percent	Cumulative Frequency	Cumulative Percent
Home	22	2.88	22	2.88
Commercial	114	14.90	136	17.78
Non-commercial	427	55.82	563	73.59
Head start	64	8.37	627	81.96
Religious	105	13.73	732	95.69
Other	33	4.31	765	100.00

Summary

How many children are cared for in your childhood work setting?				
**number_of_children_category_id**	**Frequency**	**Percent**	**Cumulative Frequency**	**Cumulative Percent**
**<10**	46	6.01	46	6.01
**10-25**	253	33.07	299	39.08
**>25**	466	60.92	765	100.00

On what basis are you currently employed?				
**employment_type_id**	**Frequency**	**Percent**	**Cumulative Frequency**	**Cumulative Percent**
**Permanent full-time**	552	72.16	552	72.16
**Permanent part-time**	171	22.35	723	94.51
**Substitute teacher**	6	0.78	729	95.29
**Seasonal**	29	3.79	758	99.08
**Volunteer**	2	0.26	760	99.35
**Other**	5	0.65	765	100.00

Including this year, how many years have you worked as an early childhood practitioner?				
**years_as_practitioner_id**	**Frequency**	**Percent**	**Cumulative Frequency**	**Cumulative Percent**
**<1**	68	8.89	68	8.89
**1-2**	117	15.29	185	24.18
**3-5**	151	19.74	336	43.92
**6-10**	159	20.78	495	64.71
**11-15**	76	9.93	571	74.64
**>15**	194	25.36	765	100.00

Summary

Including this year, how many years have you worked at your current workplace?				
years_at_workplace_id	Frequency	Percent	Cumulative Frequency	Cumulative Percent
<1	160	20.92	160	20.92
1-2	165	21.57	325	42.48
3-5	160	20.92	485	63.40
6-10	136	17.78	621	81.18
11-15	55	7.19	676	88.37
>15	89	11.63	765	100.00

In your work as an early childhood practitioner, have you ever reported a case of child abuse/neglect?				
have_reported_abuse_id	Frequency	Percent	Cumulative Frequency	Cumulative Percent
No	527	68.89	527	68.89
Once	98	12.81	625	81.70
2-5 times	100	13.07	725	94.77
6-10 times	16	2.09	741	96.86
More than 10 times	8	1.05	749	97.91
No answer	16	2.09	765	100.00

In your work as an early childhood practitioner, have you ever suspected a case of child abuse/neglect and not reported it?				
have_suspected_abuse_id	Frequency	Percent	Cumulative Frequency	Cumulative Percent
No	638	83.40	638	83.40
Once	57	7.45	695	90.85
2-5 times	36	4.71	731	95.56
6-10 times	3	0.39	734	95.95
No answer	31	4.05	765	100.00

Has concern for abuse infant				
has_consern_for_abuse_infant	Frequency	Percent	Cumulative Frequency	Cumulative Percent
no	134	17.52	134	17.52
YES	631	82.48	765	100.00

Summary

Has concern for abuse age younger than 5				
has_consern_for_abuse_younger5	Frequency	Percent	Cumulative Frequency	Cumulative Percent
no	540	70.59	540	70.59
YES	225	29.41	765	100.00

Has concern for abuse for spanking				
has_consern_for_abuse_spanking	Frequency	Percent	Cumulative Frequency	Cumulative Percent
no	154	20.13	154	20.13
YES	611	79.87	765	100.00

Has concern for abuse for toddler shin				
has_consern_for_abuse_shin	Frequency	Percent	Cumulative Frequency	Cumulative Percent
no	619	80.92	619	80.92
YES	146	19.08	765	100.00

Has concern for abuse for toddler ear				
has_consern_for_abuse_ear	Frequency	Percent	Cumulative Frequency	Cumulative Percent
no	267	34.90	267	34.90
YES	498	65.10	765	100.00

Has concern for abuse for none				
has_consern_for_abuse_none	Frequency	Percent	Cumulative Frequency	Cumulative Percent
no	748	97.78	748	97.78
YES	17	2.22	765	100.00

Non-Hispanic White ethnicity				
has_ethnicity_white	Frequency	Percent	Cumulative Frequency	Cumulative Percent
no	118	15.42	118	15.42
YES	647	84.58	765	100.00

Summary

Non-Hispanic Black ethnicity				
has_ethnicity_african_american	Frequency	Percent	Cumulative Frequency	Cumulative Percent
no	697	91.11	697	91.11
YES	68	8.89	765	100.00

Hispanic ethnicity				
has_ethnicity_hispanic	Frequency	Percent	Cumulative Frequency	Cumulative Percent
no	740	96.73	740	96.73
YES	25	3.27	765	100.00

Native American ethnicity				
has_ethnicity_native_american	Frequency	Percent	Cumulative Frequency	Cumulative Percent
no	762	99.61	762	99.61
YES	3	0.39	765	100.00

Asian ethnicity				
has_ethnicity_asian	Frequency	Percent	Cumulative Frequency	Cumulative Percent
no	748	97.78	748	97.78
YES	17	2.22	765	100.00

Native Hawaiian ethnicity				
has_ethnicity_native_hawaiian	Frequency	Percent	Cumulative Frequency	Cumulative Percent
no	764	99.87	764	99.87
YES	1	0.13	765	100.00

Other ethnicity				
has_ethnicity_other	Frequency	Percent	Cumulative Frequency	Cumulative Percent
no	752	98.30	752	98.30
YES	13	1.70	765	100.00
