# Supplementary material for: Educating early childhood care and education providers to improve knowledge and attitudes about reporting child maltreatment: A randomized controlled trial
Source: PLoS One. 2017 May 19;12(5):e0177777. doi: 10.1371/journal.pone.0177777 (PMC5438118; doi:10.1371/journal.pone.0177777)
Supplement: S4 File — (PDF) [file pone.0177777.s005.pdf]

Data dictionary

S4 File Data repository - Knowledge

Data Set Name	WORK.KNOW	Observations	1857
Member Type	DATA	Variables	54
Engine	V9	Indexes	0
Created	08/15/2014 10:53:47	Observation Length	432
Last Modified	08/15/2014 10:53:47	Deleted Observations	0
Protection		Compressed	NO
Data Set Type		Sorted	NO
Label			
Data Representation	WINDOWS_64		
Encoding	wlatin1 Western (Windows)		

Alphabetic List of Variables and Attributes						
#	Variable	Type	Len	Format	Informat	Label
4	date	Num	8	MMDDYY10.		Date of submission
1	dim_user_id	Num	8	BEST12.	BEST32.	dim_user_idect ID
2	event	Num	8	LESSONTYPEID.		Time
3	group	Num	8	GRP.		Study group
23	know_q4	Num	8	KNOWQ4F.		Under recently enacted Pennsylvania law, for an act (or failure to act) to count as abuse/neglect, which of the following must be true?
24	know_q5	Num	8	KNOWQ5F.		Under Pennsylvania law, you can be held legally liable if you suspect child abuse/neglect and report it, but it turns out to be unfounded.
25	know_q6	Num	8	KNOWQ6F.		Under Pennsylvania law, once you have reasonable cause to suspect child abuse/neglect, you must report your suspicion to authorities:
26	know_q7	Num	8	KNOWQ7F.		Under Pennsylvania law, you must report:
27	know_q8	Num	8	KNOWQ8F.		According to newly enacted Pennsylvania law, you are required to report suspected child abuse/neglect to:
28	know_q9	Num	8	KNOWQ9F.		To count as physical child abuse, it must result in a child experiencing:
21	know_q10	Num	8	YN.		Under Pennsylvania law, are you required to report suspected child abuse/neglect if a child was put at significant risk for being injured even when no injury or harm actually occurred?
29	know_q11	Num	8	KNOWQ11F.		Requirements for reporting suspected child abuse/neglect refer to recent events. How long ago counts as recent under Pennsylvania law?
22	know_q12	Num	8	YN.		Do you feel confident in your ability to identify signs of child abuse/neglect?
30	know_q13	Num	8	KNOWQ13F.		How prepared do you feel to report child abuse/neglect should the need arise?
52	know_q10_correct	Num	8	YN.		Knowledge Question 10 correct
53	know_q11_correct	Num	8	YN.		Knowledge Question 11 correct

Data dictionary

Alphabetic List of Variables and Attributes						
**#**	**Variable**	**Type**	**Len**	**Format**	**Informat**	**Label**
**6**	know_q1a	Num	8	YN.		Physically restrain a child by locking them in a closet
**31**	know_q1a_correct	Num	8	YN.		Knowledge Question 1a correct
**7**	know_q1b	Num	8	YN.		Place duct tape over a child’s mouth as a form of punishment
**32**	know_q1b_correct	Num	8	YN.		Knowledge Question 1b correct
**8**	know_q1c	Num	8	YN.		Cause any kind of physical injury
**33**	know_q1c_correct	Num	8	YN.		Knowledge Question 1c correct
**9**	know_q1d	Num	8	YN.		Cause substantial pain from disciplining a child
**34**	know_q1d_correct	Num	8	YN.		Knowledge Question 1d correct
**10**	know_q1e	Num	8	YN.		Impair physical functioning from disciplining a child
**35**	know_q1e_correct	Num	8	YN.		Knowledge Question 1e correct
**11**	know_q1f	Num	8	YN.		Forcefully slap a child under one year of age
**36**	know_q1f_correct	Num	8	YN.		Knowledge Question 1f correct
**12**	know_q1g	Num	8	YN.		Expose a child to domestic violence
**37**	know_q1g_correct	Num	8	YN.		Knowledge Question 1g correct
**13**	know_q2a	Num	8	YN.		Any bruising in an infant who hasn’t started pulling to stand
**38**	know_q2a_correct	Num	8	YN.		Knowledge Question 2a correct
**14**	know_q2b	Num	8	YN.		Any bruising in a child younger than 5 years old
**39**	know_q2b_correct	Num	8	YN.		Knowledge Question 2b correct
**15**	know_q2c	Num	8	YN.		Any bruising from spanking
**40**	know_q2c_correct	Num	8	YN.		Knowledge Question 2c correct
**16**	know_q2d	Num	8	YN.		Bruising on toddler’s shins
**41**	know_q2d_correct	Num	8	YN.		Knowledge Question 2d correct
**17**	know_q2e	Num	8	YN.		Bruising on toddler’s ears
**42**	know_q2e_correct	Num	8	YN.		Knowledge Question 2e correct
**18**	know_q3a	Num	8	YN.		Financial penalty
**43**	know_q3a_correct	Num	8	YN.		Knowledge Question 3a correct
**19**	know_q3b	Num	8	YN.		Loss of professional license
**44**	know_q3b_correct	Num	8	YN.		Knowledge Question 3b correct
**20**	know_q3c	Num	8	YN.		Incarceration
**45**	know_q3c_correct	Num	8	YN.		Knowledge Question 3c correct
**46**	know_q4_correct	Num	8	YN.		Knowledge Question 4 correct
**47**	know_q5_correct	Num	8	YN.		Knowledge Question 5 correct
**48**	know_q6_correct	Num	8	YN.		Knowledge Question 6 correct
**49**	know_q7_correct	Num	8	YN.		Knowledge Question 7 correct

Data dictionary

Alphabetic List of Variables and Attributes						
#	Variable	Type	Len	Format	Informat	Label
50	know_q8_correct	Num	8	YN.		Knowledge Question 8 correct
51	know_q9_correct	Num	8	YN.		Knowledge Question 9 correct
54	know_score	Num	8			Total knowledge score
5	time	Num	8	TIME.		Time of submission

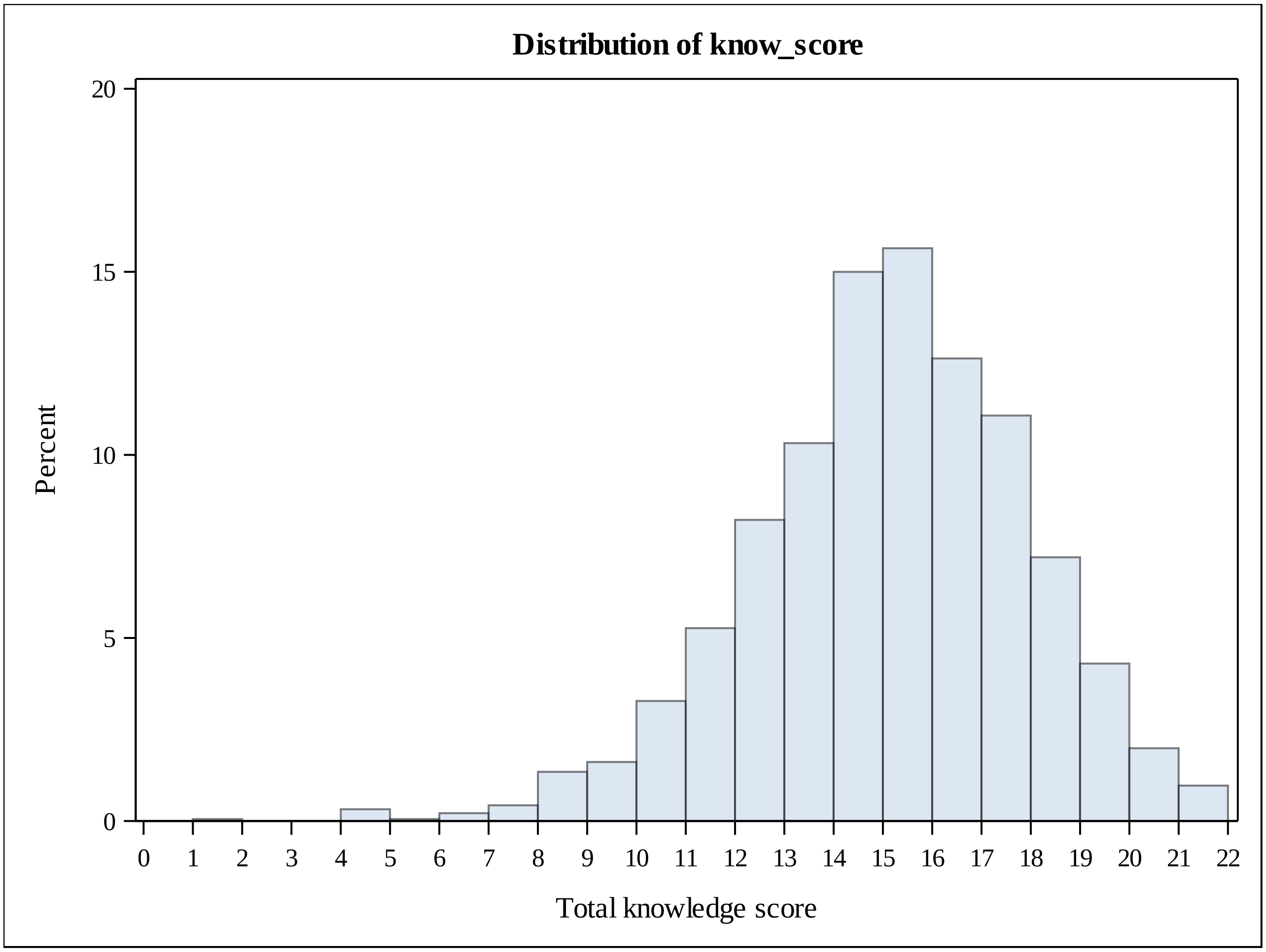

Analysis Variable : know_score Total knowledge score							
**N**	**N Miss**	**Mean**	**Std Dev**	**Median**	**Quartile Range**	**Minimum**	**Maximum**
1856	1	14.62	2.80	15.00	4.00	1.00	21.00

Analysis Variable : know_score Total knowledge score										
**Study group**	**Time**	**N Obs**	**N**	**N Miss**	**Mean**	**Std Dev**	**Median**	**Quartile Range**	**Minimum**	**Maximum**
Control	Pre-survey	371	371	0	13.53	2.66	14.00	3.00	4.00	21.00
	Post-survey	365	365	0	16.30	2.09	16.00	3.00	9.00	21.00
	Re-test	371	371	0	13.59	2.64	14.00	3.00	1.00	21.00
Experimental	Pre-survey	377	376	1	13.52	2.60	14.00	3.00	4.00	21.00
	Post-survey	373	373	0	16.20	2.29	16.00	3.00	4.00	21.00

Total knowledge score				
**know_score**	**Frequency**	**Percent**	**Cumulative Frequency**	**Cumulative Percent**
**1**	1	0.05	1	0.05
**4**	6	0.32	7	0.38
**5**	1	0.05	8	0.43
**6**	4	0.22	12	0.65
**7**	8	0.43	20	1.08
**8**	25	1.35	45	2.42
**9**	30	1.62	75	4.04
**10**	61	3.29	136	7.33
**11**	98	5.28	234	12.61
**12**	153	8.24	387	20.85
**13**	192	10.34	579	31.20
**14**	278	14.98	857	46.17
**15**	290	15.63	1147	61.80
**16**	234	12.61	1381	74.41
**17**	206	11.10	1587	85.51
**18**	134	7.22	1721	92.73
**19**	80	4.31	1801	97.04
**20**	37	1.99	1838	99.03
**21**	18	0.97	1856	100.00
**Frequency Missing = 1**				

Physically restrain a child by locking them in a closet				
**know_q1a**	**Frequency**	**Percent**	**Cumulative Frequency**	**Cumulative Percent**
**no**	33	1.78	33	1.78
**YES**	1786	96.18	1819	97.95
**Unsure**	38	2.05	1857	100.00

Table 1 of know_q1a by event				
**Controlling for group=Control**				
**know_q1a(Physically restrain a child by locking them in a closet)**	**event(Time)**			
**Frequency<br>Percent<br>Row Pct<br>Col Pct**	**Pre-survey**	**Post-survey**	**Re-test**	**Total**
**no**	11<br>0.99<br>57.89<br>2.96	1<br>0.09<br>5.26<br>0.27	7<br>0.63<br>36.84<br>1.89	19<br>1.72
**YES**	349<br>31.53<br>32.83<br>94.07	363<br>32.79<br>34.15<br>99.45	351<br>31.71<br>33.02<br>94.61	1063<br>96.03
**Unsure**	11<br>0.99<br>44.00<br>2.96	1<br>0.09<br>4.00<br>0.27	13<br>1.17<br>52.00<br>3.50	25<br>2.26
**Total**	371<br>33.51	365<br>32.97	371<br>33.51	1107<br>100.00

Table 2 of know_q1a by event				
Controlling for group=Experimental				
know_q1a(Physically restrain a child by locking them in a closet)	event(Time)			
Frequency<br>Percent<br>Row Pct<br>Col Pct	Pre-survey	Post-survey	Re-test	Total
no	8<br>1.07<br>57.14<br>2.12	6<br>0.80<br>42.86<br>1.61	0<br>0.00<br>0.00<br>.	14<br>1.87
YES	357<br>47.60<br>49.38<br>94.69	366<br>48.80<br>50.62<br>98.12	0<br>0.00<br>0.00<br>.	723<br>96.40
Unsure	12<br>1.60<br>92.31<br>3.18	1<br>0.13<br>7.69<br>0.27	0<br>0.00<br>0.00<br>.	13<br>1.73
Total	377<br>50.27	373<br>49.73	0<br>0.00	750<br>100.00

Knowledge Question 1a correct				
know_q1a_correct	Frequency	Percent	Cumulative Frequency	Cumulative Percent
no	71	3.82	71	3.82
YES	1786	96.18	1857	100.00

Table 1 of know_q1a_correct by event<br>Controlling for group=Control				
know_q1a_correct(Knowledge Question 1a correct)	event(Time)			
Frequency<br>Percent<br>Row Pct<br>Col Pct	Pre-survey	Post-survey	Re-test	Total
no	22<br>1.99<br>50.00<br>5.93	2<br>0.18<br>4.55<br>0.55	20<br>1.81<br>45.45<br>5.39	44<br>3.97
YES	349<br>31.53<br>32.83<br>94.07	363<br>32.79<br>34.15<br>99.45	351<br>31.71<br>33.02<br>94.61	1063<br>96.03
Total	371<br>33.51	365<br>32.97	371<br>33.51	1107<br>100.00

Table 2 of know_q1a_correct by event<br>Controlling for group=Experimental				
know_q1a_correct(Knowledge Question 1a correct)	event(Time)			
Frequency<br>Percent<br>Row Pct<br>Col Pct	Pre-survey	Post-survey	Re-test	Total
no	20<br>2.67<br>74.07<br>5.31	7<br>0.93<br>25.93<br>1.88	0<br>0.00<br>0.00<br>.	27<br>3.60
YES	357<br>47.60<br>49.38<br>94.69	366<br>48.80<br>50.62<br>98.12	0<br>0.00<br>0.00<br>.	723<br>96.40
Total	377<br>50.27	373<br>49.73	0<br>0.00	750<br>100.00

Place duct tape over a child’s mouth as a form of punishment				
know_q1b	Frequency	Percent	Cumulative Frequency	Cumulative Percent
no	38	2.05	38	2.05
YES	1771	95.37	1809	97.42
Unsure	48	2.58	1857	100.00

Table 1 of know_q1b by event				
Controlling for group=Control				
know_q1b(Place duct tape over a child’s mouth as a form of punishment)	event(Time)			
Frequency<br>Percent<br>Row Pct<br>Col Pct	Pre-survey	Post-survey	Re-test	Total
no	11<br>0.99<br>55.00<br>2.96	4<br>0.36<br>20.00<br>1.10	5<br>0.45<br>25.00<br>1.35	20<br>1.81
YES	346<br>31.26<br>32.83<br>93.26	358<br>32.34<br>33.97<br>98.08	350<br>31.62<br>33.21<br>94.34	1054<br>95.21
Unsure	14<br>1.26<br>42.42<br>3.77	3<br>0.27<br>9.09<br>0.82	16<br>1.45<br>48.48<br>4.31	33<br>2.98
Total	371<br>33.51	365<br>32.97	371<br>33.51	1107<br>100.00

Table 2 of know_q1b by event				
Controlling for group=Experimental				
know_q1b(Place duct tape over a child's mouth as a form of punishment)	event(Time)			
Frequency<br>Percent<br>Row Pct<br>Col Pct	Pre-survey	Post-survey	Re-test	Total
no	9<br>1.20<br>50.00<br>2.39	9<br>1.20<br>50.00<br>2.41	0<br>0.00<br>0.00<br>.	18<br>2.40
YES	355<br>47.33<br>49.51<br>94.16	362<br>48.27<br>50.49<br>97.05	0<br>0.00<br>0.00<br>.	717<br>95.60
Unsure	13<br>1.73<br>86.67<br>3.45	2<br>0.27<br>13.33<br>0.54	0<br>0.00<br>0.00<br>.	15<br>2.00
Total	377<br>50.27	373<br>49.73	0<br>0.00	750<br>100.00

Knowledge Question 1b correct				
know_q1b_correct	Frequency	Percent	Cumulative Frequency	Cumulative Percent
**no**	86	4.63	86	4.63
**YES**	1771	95.37	1857	100.00

Table 1 of know_q1b_correct by event				
Controlling for group=Control				
know_q1b_correct(Knowledge Question 1b correct)	event(Time)			
Frequency<br>Percent<br>Row Pct<br>Col Pct	Pre-survey	Post-survey	Re-test	Total
**no**	25<br>2.26<br>47.17<br>6.74	7<br>0.63<br>13.21<br>1.92	21<br>1.90<br>39.62<br>5.66	53<br>4.79
**YES**	346<br>31.26<br>32.83<br>93.26	358<br>32.34<br>33.97<br>98.08	350<br>31.62<br>33.21<br>94.34	1054<br>95.21
**Total**	371<br>33.51	365<br>32.97	371<br>33.51	1107<br>100.00

Table 2 of know_q1b_correct by event				
Controlling for group=Experimental				
know_q1b_correct(Knowledge Question 1b correct)	event(Time)			
Frequency<br>Percent<br>Row Pct<br>Col Pct	Pre-survey	Post-survey	Re-test	Total
**no**	22<br>2.93<br>66.67<br>5.84	11<br>1.47<br>33.33<br>2.95	0<br>0.00<br>0.00<br>.	33<br>4.40
**YES**	355<br>47.33<br>49.51<br>94.16	362<br>48.27<br>50.49<br>97.05	0<br>0.00<br>0.00<br>.	717<br>95.60
**Total**	377<br>50.27	373<br>49.73	0<br>0.00	750<br>100.00

Cause any kind of physical injury				
know_q1c	Frequency	Percent	Cumulative Frequency	Cumulative Percent
no	146	7.86	146	7.86
YES	1598	86.05	1744	93.91
Unsure	113	6.09	1857	100.00

Table 1 of know_q1c by event				
Controlling for group=Control				
know_q1c(Cause any kind of physical injury)	event(Time)			
Frequency<br>Percent<br>Row Pct<br>Col Pct	Pre-survey	Post-survey	Re-test	Total
no	23<br>2.08<br>28.05<br>6.20	36<br>3.25<br>43.90<br>9.86	23<br>2.08<br>28.05<br>6.20	82<br>7.41
YES	313<br>28.27<br>33.19<br>84.37	322<br>29.09<br>34.15<br>88.22	308<br>27.82<br>32.66<br>83.02	943<br>85.19
Unsure	35<br>3.16<br>42.68<br>9.43	7<br>0.63<br>8.54<br>1.92	40<br>3.61<br>48.78<br>10.78	82<br>7.41
Total	371<br>33.51	365<br>32.97	371<br>33.51	1107<br>100.00

Table 2 of know_q1c by event				
Controlling for group=Experimental				
know_q1c(Cause any kind of physical injury)	event(Time)			
Frequency<br>Percent<br>Row Pct<br>Col Pct	Pre-survey	Post-survey	Re-test	Total
**no**	27<br>3.60<br>42.19<br>7.16	37<br>4.93<br>57.81<br>9.92	0<br>0.00<br>0.00<br>.	64<br>8.53
**YES**	324<br>43.20<br>49.47<br>85.94	331<br>44.13<br>50.53<br>88.74	0<br>0.00<br>0.00<br>.	655<br>87.33
**Unsure**	26<br>3.47<br>83.87<br>6.90	5<br>0.67<br>16.13<br>1.34	0<br>0.00<br>0.00<br>.	31<br>4.13
**Total**	377<br>50.27	373<br>49.73	0<br>0.00	750<br>100.00

Knowledge Question 1c correct				
**know_q1c_correct**	**Frequency**	**Percent**	**Cumulative Frequency**	**Cumulative Percent**
**no**	1711	92.14	1711	92.14
**YES**	146	7.86	1857	100.00

Table 1 of know_q1c_correct by event				
**Controlling for group=Control**				
**know_q1c_correct(Knowledge Question 1c correct)**	**event(Time)**			
**Frequency<br>Percent<br>Row Pct<br>Col Pct**	**Pre-survey**	**Post-survey**	**Re-test**	**Total**
**no**	348<br>31.44<br>33.95<br>93.80	329<br>29.72<br>32.10<br>90.14	348<br>31.44<br>33.95<br>93.80	1025<br>92.59
**YES**	23<br>2.08<br>28.05<br>6.20	36<br>3.25<br>43.90<br>9.86	23<br>2.08<br>28.05<br>6.20	82<br>7.41
**Total**	371<br>33.51	365<br>32.97	371<br>33.51	1107<br>100.00

Table 2 of know_q1c_correct by event				
**Controlling for group=Experimental**				
**know_q1c_correct(Knowledge Question 1c correct)**	**event(Time)**			
**Frequency<br>Percent<br>Row Pct<br>Col Pct**	**Pre-survey**	**Post-survey**	**Re-test**	**Total**
**no**	350<br>46.67<br>51.02<br>92.84	336<br>44.80<br>48.98<br>90.08	0<br>0.00<br>0.00<br>.	686<br>91.47
**YES**	27<br>3.60<br>42.19<br>7.16	37<br>4.93<br>57.81<br>9.92	0<br>0.00<br>0.00<br>.	64<br>8.53
**Total**	377<br>50.27	373<br>49.73	0<br>0.00	750<br>100.00

Cause substantial pain from disciplining a child				
know_q1d	Frequency	Percent	Cumulative Frequency	Cumulative Percent
no	35	1.88	35	1.88
YES	1761	94.83	1796	96.72
Unsure	61	3.28	1857	100.00

Table 1 of know_q1d by event				
Controlling for group=Control				
know_q1d(Cause substantial pain from disciplining a child)	event(Time)			
Frequency<br>Percent<br>Row Pct<br>Col Pct	Pre-survey	Post-survey	Re-test	Total
no	9<br>0.81<br>56.25<br>2.43	3<br>0.27<br>18.75<br>0.82	4<br>0.36<br>25.00<br>1.08	16<br>1.45
YES	340<br>30.71<br>32.26<br>91.64	360<br>32.52<br>34.16<br>98.63	354<br>31.98<br>33.59<br>95.42	1054<br>95.21
Unsure	22<br>1.99<br>59.46<br>5.93	2<br>0.18<br>5.41<br>0.55	13<br>1.17<br>35.14<br>3.50	37<br>3.34
Total	371<br>33.51	365<br>32.97	371<br>33.51	1107<br>100.00

Table 2 of know_q1d by event				
Controlling for group=Experimental				
know_q1d(Cause substantial pain from disciplining a child)	event(Time)			
Frequency<br>Percent<br>Row Pct<br>Col Pct	Pre-survey	Post-survey	Re-test	Total
no	13<br>1.73<br>68.42<br>3.45	6<br>0.80<br>31.58<br>1.61	0<br>0.00<br>0.00<br>.	19<br>2.53
YES	342<br>45.60<br>48.37<br>90.72	365<br>48.67<br>51.63<br>97.86	0<br>0.00<br>0.00<br>.	707<br>94.27
Unsure	22<br>2.93<br>91.67<br>5.84	2<br>0.27<br>8.33<br>0.54	0<br>0.00<br>0.00<br>.	24<br>3.20
Total	377<br>50.27	373<br>49.73	0<br>0.00	750<br>100.00

Knowledge Question 1d correct				
know_q1d_correct	Frequency	Percent	Cumulative Frequency	Cumulative Percent
no	96	5.17	96	5.17
YES	1761	94.83	1857	100.00

Table 1 of know_q1d_correct by event<br>Controlling for group=Control				
know_q1d_correct(Knowledge Question 1d correct)	event(Time)			
Frequency<br>Percent<br>Row Pct<br>Col Pct	Pre-survey	Post-survey	Re-test	Total
no	31<br>2.80<br>58.49<br>8.36	5<br>0.45<br>9.43<br>1.37	17<br>1.54<br>32.08<br>4.58	53<br>4.79
YES	340<br>30.71<br>32.26<br>91.64	360<br>32.52<br>34.16<br>98.63	354<br>31.98<br>33.59<br>95.42	1054<br>95.21
Total	371<br>33.51	365<br>32.97	371<br>33.51	1107<br>100.00

Table 2 of know_q1d_correct by event<br>Controlling for group=Experimental				
know_q1d_correct(Knowledge Question 1d correct)	event(Time)			
Frequency<br>Percent<br>Row Pct<br>Col Pct	Pre-survey	Post-survey	Re-test	Total
no	35<br>4.67<br>81.40<br>9.28	8<br>1.07<br>18.60<br>2.14	0<br>0.00<br>0.00<br>.	43<br>5.73
YES	342<br>45.60<br>48.37<br>90.72	365<br>48.67<br>51.63<br>97.86	0<br>0.00<br>0.00<br>.	707<br>94.27
Total	377<br>50.27	373<br>49.73	0<br>0.00	750<br>100.00

Impair physical functioning from disciplining a child				
know_q1e	Frequency	Percent	Cumulative Frequency	Cumulative Percent
no	24	1.29	24	1.29
YES	1768	95.21	1792	96.50
Unsure	65	3.50	1857	100.00

Table 1 of know_q1e by event				
Controlling for group=Control				
know_q1e(Impair physical functioning from disciplining a child)	event(Time)			
Frequency<br>Percent<br>Row Pct<br>Col Pct	Pre-survey	Post-survey	Re-test	Total
no	6<br>0.54<br>46.15<br>1.62	2<br>0.18<br>15.38<br>0.55	5<br>0.45<br>38.46<br>1.35	13<br>1.17
YES	344<br>31.07<br>32.45<br>92.72	361<br>32.61<br>34.06<br>98.90	355<br>32.07<br>33.49<br>95.69	1060<br>95.75
Unsure	21<br>1.90<br>61.76<br>5.66	2<br>0.18<br>5.88<br>0.55	11<br>0.99<br>32.35<br>2.96	34<br>3.07
Total	371<br>33.51	365<br>32.97	371<br>33.51	1107<br>100.00

Table 2 of know_q1e by event				
Controlling for group=Experimental				
know_q1e(Impair physical functioning from disciplining a child)	event(Time)			
Frequency<br>Percent<br>Row Pct<br>Col Pct	Pre-survey	Post-survey	Re-test	Total
no	8<br>1.07<br>72.73<br>2.12	3<br>0.40<br>27.27<br>0.80	0<br>0.00<br>0.00<br>.	11<br>1.47
YES	340<br>45.33<br>48.02<br>90.19	368<br>49.07<br>51.98<br>98.66	0<br>0.00<br>0.00<br>.	708<br>94.40
Unsure	29<br>3.87<br>93.55<br>7.69	2<br>0.27<br>6.45<br>0.54	0<br>0.00<br>0.00<br>.	31<br>4.13
Total	377<br>50.27	373<br>49.73	0<br>0.00	750<br>100.00

Knowledge Question 1e correct				
know_q1e_correct	Frequency	Percent	Cumulative Frequency	Cumulative Percent
no	89	4.79	89	4.79
YES	1768	95.21	1857	100.00

Table 1 of know_q1e_correct by event				
Controlling for group=Control				
know_q1e_correct(Knowledge Question 1e correct)	event(Time)			
Frequency<br>Percent<br>Row Pct<br>Col Pct	Pre-survey	Post-survey	Re-test	Total
no	27<br>2.44<br>57.45<br>7.28	4<br>0.36<br>8.51<br>1.10	16<br>1.45<br>34.04<br>4.31	47<br>4.25
YES	344<br>31.07<br>32.45<br>92.72	361<br>32.61<br>34.06<br>98.90	355<br>32.07<br>33.49<br>95.69	1060<br>95.75
Total	371<br>33.51	365<br>32.97	371<br>33.51	1107<br>100.00

Table 2 of know_q1e_correct by event				
Controlling for group=Experimental				
know_q1e_correct(Knowledge Question 1e correct)	event(Time)			
Frequency<br>Percent<br>Row Pct<br>Col Pct	Pre-survey	Post-survey	Re-test	Total
no	37<br>4.93<br>88.10<br>9.81	5<br>0.67<br>11.90<br>1.34	0<br>0.00<br>0.00<br>.	42<br>5.60
YES	340<br>45.33<br>48.02<br>90.19	368<br>49.07<br>51.98<br>98.66	0<br>0.00<br>0.00<br>.	708<br>94.40
Total	377<br>50.27	373<br>49.73	0<br>0.00	750<br>100.00

Forcefully slap a child under one year of age				
know_q1f	Frequency	Percent	Cumulative Frequency	Cumulative Percent
no	35	1.88	35	1.88
YES	1777	95.69	1812	97.58
Unsure	45	2.42	1857	100.00

Table 1 of know_q1f by event				
Controlling for group=Control				
know_q1f(Forcefully slap a child under one year of age)	event(Time)			
Frequency<br>Percent<br>Row Pct<br>Col Pct	Pre-survey	Post-survey	Re-test	Total
no	10<br>0.90<br>62.50<br>2.70	1<br>0.09<br>6.25<br>0.27	5<br>0.45<br>31.25<br>1.35	16<br>1.45
YES	347<br>31.35<br>32.46<br>93.53	363<br>32.79<br>33.96<br>99.45	359<br>32.43<br>33.58<br>96.77	1069<br>96.57
Unsure	14<br>1.26<br>63.64<br>3.77	1<br>0.09<br>4.55<br>0.27	7<br>0.63<br>31.82<br>1.89	22<br>1.99
Total	371<br>33.51	365<br>32.97	371<br>33.51	1107<br>100.00

Table 2 of know_q1f by event				
Controlling for group=Experimental				
know_q1f(Forcefully slap a child under one year of age)	event(Time)			
Frequency<br>Percent<br>Row Pct<br>Col Pct	Pre-survey	Post-survey	Re-test	Total
no	15<br>2.00<br>78.95<br>3.98	4<br>0.53<br>21.05<br>1.07	0<br>0.00<br>0.00<br>.	19<br>2.53
YES	340<br>45.33<br>48.02<br>90.19	368<br>49.07<br>51.98<br>98.66	0<br>0.00<br>0.00<br>.	708<br>94.40
Unsure	22<br>2.93<br>95.65<br>5.84	1<br>0.13<br>4.35<br>0.27	0<br>0.00<br>0.00<br>.	23<br>3.07
Total	377<br>50.27	373<br>49.73	0<br>0.00	750<br>100.00

Knowledge Question 1f correct				
know_q1f_correct	Frequency	Percent	Cumulative Frequency	Cumulative Percent
no	80	4.31	80	4.31
YES	1777	95.69	1857	100.00

Table 1 of know_q1f_correct by event				
Controlling for group=Control				
know_q1f_correct(Knowledge Question 1f correct)	event(Time)			
Frequency<br>Percent<br>Row Pct<br>Col Pct	Pre-survey	Post-survey	Re-test	Total
no	24<br>2.17<br>63.16<br>6.47	2<br>0.18<br>5.26<br>0.55	12<br>1.08<br>31.58<br>3.23	38<br>3.43
YES	347<br>31.35<br>32.46<br>93.53	363<br>32.79<br>33.96<br>99.45	359<br>32.43<br>33.58<br>96.77	1069<br>96.57
Total	371<br>33.51	365<br>32.97	371<br>33.51	1107<br>100.00

Table 2 of know_q1f_correct by event				
Controlling for group=Experimental				
know_q1f_correct(Knowledge Question 1f correct)	event(Time)			
Frequency<br>Percent<br>Row Pct<br>Col Pct	Pre-survey	Post-survey	Re-test	Total
no	37<br>4.93<br>88.10<br>9.81	5<br>0.67<br>11.90<br>1.34	0<br>0.00<br>0.00<br>.	42<br>5.60
YES	340<br>45.33<br>48.02<br>90.19	368<br>49.07<br>51.98<br>98.66	0<br>0.00<br>0.00<br>.	708<br>94.40
Total	377<br>50.27	373<br>49.73	0<br>0.00	750<br>100.00

Expose a child to domestic violence				
know_q1g	Frequency	Percent	Cumulative Frequency	Cumulative Percent
no	230	12.39	230	12.39
YES	1431	77.06	1661	89.45
Unsure	196	10.55	1857	100.00

Table 1 of know_q1g by event				
Controlling for group=Control				
know_q1g(Expose a child to domestic violence)	event(Time)			
Frequency<br>Percent<br>Row Pct<br>Col Pct	Pre-survey	Post-survey	Re-test	Total
no	31<br>2.80<br>24.41<br>8.36	71<br>6.41<br>55.91<br>19.45	25<br>2.26<br>19.69<br>6.74	127<br>11.47
YES	277<br>25.02<br>32.25<br>74.66	286<br>25.84<br>33.29<br>78.36	296<br>26.74<br>34.46<br>79.78	859<br>77.60
Unsure	63<br>5.69<br>52.07<br>16.98	8<br>0.72<br>6.61<br>2.19	50<br>4.52<br>41.32<br>13.48	121<br>10.93
Total	371<br>33.51	365<br>32.97	371<br>33.51	1107<br>100.00

Table 2 of know_q1g by event				
Controlling for group=Experimental				
know_q1g(Expose a child to domestic violence)	event(Time)			
Frequency<br>Percent<br>Row Pct<br>Col Pct	Pre-survey	Post-survey	Re-test	Total
no	27<br>3.60<br>26.21<br>7.16	76<br>10.13<br>73.79<br>20.38	0<br>0.00<br>0.00<br>.	103<br>13.73
YES	282<br>37.60<br>49.30<br>74.80	290<br>38.67<br>50.70<br>77.75	0<br>0.00<br>0.00<br>.	572<br>76.27
Unsure	68<br>9.07<br>90.67<br>18.04	7<br>0.93<br>9.33<br>1.88	0<br>0.00<br>0.00<br>.	75<br>10.00
Total	377<br>50.27	373<br>49.73	0<br>0.00	750<br>100.00

Knowledge Question 1g correct				
know_q1g_correct	Frequency	Percent	Cumulative Frequency	Cumulative Percent
no	1627	87.61	1627	87.61
YES	230	12.39	1857	100.00

Table 1 of know_q1g_correct by event				
Controlling for group=Control				
know_q1g_correct(Knowledge Question 1g correct)	event(Time)			
Frequency<br>Percent<br>Row Pct<br>Col Pct	Pre-survey	Post-survey	Re-test	Total
no	340<br>30.71<br>34.69<br>91.64	294<br>26.56<br>30.00<br>80.55	346<br>31.26<br>35.31<br>93.26	980<br>88.53
YES	31<br>2.80<br>24.41<br>8.36	71<br>6.41<br>55.91<br>19.45	25<br>2.26<br>19.69<br>6.74	127<br>11.47
Total	371<br>33.51	365<br>32.97	371<br>33.51	1107<br>100.00

Table 2 of know_q1g_correct by event				
Controlling for group=Experimental				
know_q1g_correct(Knowledge Question 1g correct)	event(Time)			
Frequency<br>Percent<br>Row Pct<br>Col Pct	Pre-survey	Post-survey	Re-test	Total
no	350<br>46.67<br>54.10<br>92.84	297<br>39.60<br>45.90<br>79.62	0<br>0.00<br>0.00<br>.	647<br>86.27
YES	27<br>3.60<br>26.21<br>7.16	76<br>10.13<br>73.79<br>20.38	0<br>0.00<br>0.00<br>.	103<br>13.73
Total	377<br>50.27	373<br>49.73	0<br>0.00	750<br>100.00

Any bruising in an infant who hasn't started pulling to stand				
know_q2a	Frequency	Percent	Cumulative Frequency	Cumulative Percent
no	71	3.83	71	3.83
YES	1662	89.55	1733	93.37
Unsure	123	6.63	1856	100.00
Frequency Missing = 1				

Table 1 of know_q2a by event				
Controlling for group=Control				
know_q2a(Any bruising in an infant who hasn't started pulling to stand)	event(Time)			
Frequency<br>Percent<br>Row Pct<br>Col Pct	Pre-survey	Post-survey	Re-test	Total
no	20<br>1.81<br>45.45<br>5.39	4<br>0.36<br>9.09<br>1.10	20<br>1.81<br>45.45<br>5.39	44<br>3.97
YES	308<br>27.82<br>31.27<br>83.02	360<br>32.52<br>36.55<br>98.63	317<br>28.64<br>32.18<br>85.44	985<br>88.98
Unsure	43<br>3.88<br>55.13<br>11.59	1<br>0.09<br>1.28<br>0.27	34<br>3.07<br>43.59<br>9.16	78<br>7.05
Total	371<br>33.51	365<br>32.97	371<br>33.51	1107<br>100.00

Table 2 of know_q2a by event				
Controlling for group=Experimental				
know_q2a(Any bruising in an infant who hasn't started pulling to stand)	event(Time)			
Frequency<br>Percent<br>Row Pct<br>Col Pct	Pre-survey	Post-survey	Re-test	Total
no	19<br>2.54<br>70.37<br>5.05	8<br>1.07<br>29.63<br>2.14	0<br>0.00<br>0.00<br>.	27<br>3.60
YES	316<br>42.19<br>46.68<br>84.04	361<br>48.20<br>53.32<br>96.78	0<br>0.00<br>0.00<br>.	677<br>90.39
Unsure	41<br>5.47<br>91.11<br>10.90	4<br>0.53<br>8.89<br>1.07	0<br>0.00<br>0.00<br>.	45<br>6.01
Total	376<br>50.20	373<br>49.80	0<br>0.00	749<br>100.00
Frequency Missing = 1				

Knowledge Question 2a correct				
**know_q2a_correct**	**Frequency**	**Percent**	**Cumulative Frequency**	**Cumulative Percent**
**no**	194	10.45	194	10.45
**YES**	1662	89.55	1856	100.00
**Frequency Missing = 1**				

Table 1 of know_q2a_correct by event				
**Controlling for group=Control**				
**know_q2a_correct(Knowledge Question 2a correct)**	**event(Time)**			
**Frequency<br>Percent<br>Row Pct<br>Col Pct**	**Pre-survey**	**Post-survey**	**Re-test**	**Total**
**no**	63<br>5.69<br>51.64<br>16.98	5<br>0.45<br>4.10<br>1.37	54<br>4.88<br>44.26<br>14.56	122<br>11.02
**YES**	308<br>27.82<br>31.27<br>83.02	360<br>32.52<br>36.55<br>98.63	317<br>28.64<br>32.18<br>85.44	985<br>88.98
**Total**	371<br>33.51	365<br>32.97	371<br>33.51	1107<br>100.00

Table 2 of know_q2a_correct by event				
**Controlling for group=Experimental**				
**know_q2a_correct(Knowledge Question 2a correct)**	**event(Time)**			
**Frequency<br>Percent<br>Row Pct<br>Col Pct**	**Pre-survey**	**Post-survey**	**Re-test**	**Total**
**no**	60<br>8.01<br>83.33<br>15.96	12<br>1.60<br>16.67<br>3.22	0<br>0.00<br>0.00<br>.	72<br>9.61
**YES**	316<br>42.19<br>46.68<br>84.04	361<br>48.20<br>53.32<br>96.78	0<br>0.00<br>0.00<br>.	677<br>90.39
**Total**	376<br>50.20	373<br>49.80	0<br>0.00	749<br>100.00
**Frequency Missing = 1**				

Any bruising in a child younger than 5 years old				
know_q2b	Frequency	Percent	Cumulative Frequency	Cumulative Percent
no	876	47.20	876	47.20
YES	686	36.96	1562	84.16
Unsure	294	15.84	1856	100.00
Frequency Missing = 1				

Table 1 of know_q2b by event				
Controlling for group=Control				
know_q2b(Any bruising in a child younger than 5 years old)	event(Time)			
Frequency<br>Percent<br>Row Pct<br>Col Pct	Pre-survey	Post-survey	Re-test	Total
no	165<br>14.91<br>32.80<br>44.47	188<br>16.98<br>37.38<br>51.51	150<br>13.55<br>29.82<br>40.43	503<br>45.44
YES	120<br>10.84<br>28.24<br>32.35	162<br>14.63<br>38.12<br>44.38	143<br>12.92<br>33.65<br>38.54	425<br>38.39
Unsure	86<br>7.77<br>48.04<br>23.18	15<br>1.36<br>8.38<br>4.11	78<br>7.05<br>43.58<br>21.02	179<br>16.17
Total	371<br>33.51	365<br>32.97	371<br>33.51	1107<br>100.00

Table 2 of know_q2b by event				
Controlling for group=Experimental				
know_q2b(Any bruising in a child younger than 5 years old)	event(Time)			
Frequency<br>Percent<br>Row Pct<br>Col Pct	Pre-survey	Post-survey	Re-test	Total
no	167<br>22.30<br>44.77<br>44.41	206<br>27.50<br>55.23<br>55.23	0<br>0.00<br>0.00<br>.	373<br>49.80
YES	116<br>15.49<br>44.44<br>30.85	145<br>19.36<br>55.56<br>38.87	0<br>0.00<br>0.00<br>.	261<br>34.85
Unsure	93<br>12.42<br>80.87<br>24.73	22<br>2.94<br>19.13<br>5.90	0<br>0.00<br>0.00<br>.	115<br>15.35
Total	376<br>50.20	373<br>49.80	0<br>0.00	749<br>100.00
Frequency Missing = 1				

Knowledge Question 2b correct				
know_q2b_correct	Frequency	Percent	Cumulative Frequency	Cumulative Percent
no	980	52.80	980	52.80
YES	876	47.20	1856	100.00
Frequency Missing = 1				

Table 1 of know_q2b_correct by event				
Controlling for group=Control				
know_q2b_correct(Knowledge Question 2b correct)	event(Time)			
Frequency<br>Percent<br>Row Pct<br>Col Pct	Pre-survey	Post-survey	Re-test	Total
no	206<br>18.61<br>34.11<br>55.53	177<br>15.99<br>29.30<br>48.49	221<br>19.96<br>36.59<br>59.57	604<br>54.56
YES	165<br>14.91<br>32.80<br>44.47	188<br>16.98<br>37.38<br>51.51	150<br>13.55<br>29.82<br>40.43	503<br>45.44
Total	371<br>33.51	365<br>32.97	371<br>33.51	1107<br>100.00

Table 2 of know_q2b_correct by event				
Controlling for group=Experimental				
know_q2b_correct(Knowledge Question 2b correct)	event(Time)			
Frequency<br>Percent<br>Row Pct<br>Col Pct	Pre-survey	Post-survey	Re-test	Total
no	209<br>27.90<br>55.59<br>55.59	167<br>22.30<br>44.41<br>44.77	0<br>0.00<br>0.00<br>.	376<br>50.20
YES	167<br>22.30<br>44.77<br>44.41	206<br>27.50<br>55.23<br>55.23	0<br>0.00<br>0.00<br>.	373<br>49.80
Total	376<br>50.20	373<br>49.80	0<br>0.00	749<br>100.00
Frequency Missing = 1				

Any bruising from spanking				
know_q2c	Frequency	Percent	Cumulative Frequency	Cumulative Percent
no	51	2.75	51	2.75
YES	1714	92.35	1765	95.10
Unsure	91	4.90	1856	100.00
Frequency Missing = 1				

Table 1 of know_q2c by event				
Controlling for group=Control				
know_q2c(Any bruising from spanking)	event(Time)			
Frequency<br>Percent<br>Row Pct<br>Col Pct	Pre-survey	Post-survey	Re-test	Total
no	7<br>0.63<br>38.89<br>1.89	7<br>0.63<br>38.89<br>1.92	4<br>0.36<br>22.22<br>1.08	18<br>1.63
YES	337<br>30.44<br>32.72<br>90.84	353<br>31.89<br>34.27<br>96.71	340<br>30.71<br>33.01<br>91.64	1030<br>93.04
Unsure	27<br>2.44<br>45.76<br>7.28	5<br>0.45<br>8.47<br>1.37	27<br>2.44<br>45.76<br>7.28	59<br>5.33
Total	371<br>33.51	365<br>32.97	371<br>33.51	1107<br>100.00

Table 2 of know_q2c by event				
Controlling for group=Experimental				
know_q2c(Any bruising from spanking)	event(Time)			
Frequency<br>Percent<br>Row Pct<br>Col Pct	Pre-survey	Post-survey	Re-test	Total
**no**	16<br>2.14<br>48.48<br>4.26	17<br>2.27<br>51.52<br>4.56	0<br>0.00<br>0.00<br>.	33<br>4.41
**YES**	332<br>44.33<br>48.54<br>88.30	352<br>47.00<br>51.46<br>94.37	0<br>0.00<br>0.00<br>.	684<br>91.32
**Unsure**	28<br>3.74<br>87.50<br>7.45	4<br>0.53<br>12.50<br>1.07	0<br>0.00<br>0.00<br>.	32<br>4.27
**Total**	376<br>50.20	373<br>49.80	0<br>0.00	749<br>100.00
Frequency Missing = 1				

Knowledge Question 2c correct				
know_q2c_correct	Frequency	Percent	Cumulative Frequency	Cumulative Percent
no	142	7.65	142	7.65
YES	1714	92.35	1856	100.00
Frequency Missing = 1				

Table 1 of know_q2c_correct by event				
Controlling for group=Control				
know_q2c_correct(Knowledge Question 2c correct)	event(Time)			
Frequency<br>Percent<br>Row Pct<br>Col Pct	Pre-survey	Post-survey	Re-test	Total
no	34<br>3.07<br>44.16<br>9.16	12<br>1.08<br>15.58<br>3.29	31<br>2.80<br>40.26<br>8.36	77<br>6.96
YES	337<br>30.44<br>32.72<br>90.84	353<br>31.89<br>34.27<br>96.71	340<br>30.71<br>33.01<br>91.64	1030<br>93.04
Total	371<br>33.51	365<br>32.97	371<br>33.51	1107<br>100.00

Table 2 of know_q2c_correct by event				
Controlling for group=Experimental				
know_q2c_correct(Knowledge Question 2c correct)	event(Time)			
Frequency<br>Percent<br>Row Pct<br>Col Pct	Pre-survey	Post-survey	Re-test	Total
no	44<br>5.87<br>67.69<br>11.70	21<br>2.80<br>32.31<br>5.63	0<br>0.00<br>0.00<br>.	65<br>8.68
YES	332<br>44.33<br>48.54<br>88.30	352<br>47.00<br>51.46<br>94.37	0<br>0.00<br>0.00<br>.	684<br>91.32
Total	376<br>50.20	373<br>49.80	0<br>0.00	749<br>100.00
Frequency Missing = 1				

Bruising on toddler's shins				
know_q2d	Frequency	Percent	Cumulative Frequency	Cumulative Percent
no	1091	58.78	1091	58.78
YES	476	25.65	1567	84.43
Unsure	289	15.57	1856	100.00
Frequency Missing = 1				

Table 1 of know_q2d by event				
Controlling for group=Control				
know_q2d(Bruising on toddler's shins)	event(Time)			
Frequency<br>Percent<br>Row Pct<br>Col Pct	Pre-survey	Post-survey	Re-test	Total
no	226<br>20.42<br>34.77<br>60.92	211<br>19.06<br>32.46<br>57.81	213<br>19.24<br>32.77<br>57.41	650<br>58.72
YES	65<br>5.87<br>23.64<br>17.52	131<br>11.83<br>47.64<br>35.89	79<br>7.14<br>28.73<br>21.29	275<br>24.84
Unsure	80<br>7.23<br>43.96<br>21.56	23<br>2.08<br>12.64<br>6.30	79<br>7.14<br>43.41<br>21.29	182<br>16.44
Total	371<br>33.51	365<br>32.97	371<br>33.51	1107<br>100.00

Table 2 of know_q2d by event				
Controlling for group=Experimental				
know_q2d(Bruising on toddler's shins)	event(Time)			
Frequency<br>Percent<br>Row Pct<br>Col Pct	Pre-survey	Post-survey	Re-test	Total
**no**	218<br>29.11<br>49.43<br>57.98	223<br>29.77<br>50.57<br>59.79	0<br>0.00<br>0.00<br>.	441<br>58.88
**YES**	75<br>10.01<br>37.31<br>19.95	126<br>16.82<br>62.69<br>33.78	0<br>0.00<br>0.00<br>.	201<br>26.84
**Unsure**	83<br>11.08<br>77.57<br>22.07	24<br>3.20<br>22.43<br>6.43	0<br>0.00<br>0.00<br>.	107<br>14.29
**Total**	376<br>50.20	373<br>49.80	0<br>0.00	749<br>100.00
Frequency Missing = 1				

Knowledge Question 2d correct				
know_q2d_correct	Frequency	Percent	Cumulative Frequency	Cumulative Percent
no	765	41.22	765	41.22
YES	1091	58.78	1856	100.00
Frequency Missing = 1				

Table 1 of know_q2d_correct by event				
Controlling for group=Control				
know_q2d_correct(Knowledge Question 2d correct)	event(Time)			
Frequency<br>Percent<br>Row Pct<br>Col Pct	Pre-survey	Post-survey	Re-test	Total
no	145<br>13.10<br>31.73<br>39.08	154<br>13.91<br>33.70<br>42.19	158<br>14.27<br>34.57<br>42.59	457<br>41.28
YES	226<br>20.42<br>34.77<br>60.92	211<br>19.06<br>32.46<br>57.81	213<br>19.24<br>32.77<br>57.41	650<br>58.72
Total	371<br>33.51	365<br>32.97	371<br>33.51	1107<br>100.00

Table 2 of know_q2d_correct by event				
Controlling for group=Experimental				
know_q2d_correct(Knowledge Question 2d correct)	event(Time)			
Frequency<br>Percent<br>Row Pct<br>Col Pct	Pre-survey	Post-survey	Re-test	Total
no	158<br>21.09<br>51.30<br>42.02	150<br>20.03<br>48.70<br>40.21	0<br>0.00<br>0.00<br>.	308<br>41.12
YES	218<br>29.11<br>49.43<br>57.98	223<br>29.77<br>50.57<br>59.79	0<br>0.00<br>0.00<br>.	441<br>58.88
Total	376<br>50.20	373<br>49.80	0<br>0.00	749<br>100.00
Frequency Missing = 1				

Bruising on toddler's ears				
know_q2e	Frequency	Percent	Cumulative Frequency	Cumulative Percent
no	113	6.09	113	6.09
YES	1506	81.14	1619	87.23
Unsure	237	12.77	1856	100.00
Frequency Missing = 1				

Table 1 of know_q2e by event				
Controlling for group=Control				
know_q2e(Bruising on toddler's ears)	event(Time)			
Frequency<br>Percent<br>Row Pct<br>Col Pct	Pre-survey	Post-survey	Re-test	Total
no	24<br>2.17<br>36.36<br>6.47	14<br>1.26<br>21.21<br>3.84	28<br>2.53<br>42.42<br>7.55	66<br>5.96
YES	274<br>24.75<br>30.58<br>73.85	343<br>30.98<br>38.28<br>93.97	279<br>25.20<br>31.14<br>75.20	896<br>80.94
Unsure	73<br>6.59<br>50.34<br>19.68	8<br>0.72<br>5.52<br>2.19	64<br>5.78<br>44.14<br>17.25	145<br>13.10
Total	371<br>33.51	365<br>32.97	371<br>33.51	1107<br>100.00

Table 2 of know_q2e by event				
Controlling for group=Experimental				
know_q2e(Bruising on toddler's ears)	event(Time)			
Frequency<br>Percent<br>Row Pct<br>Col Pct	Pre-survey	Post-survey	Re-test	Total
**no**	28<br>3.74<br>59.57<br>7.45	19<br>2.54<br>40.43<br>5.09	0<br>0.00<br>0.00<br>.	47<br>6.28
**YES**	265<br>35.38<br>43.44<br>70.48	345<br>46.06<br>56.56<br>92.49	0<br>0.00<br>0.00<br>.	610<br>81.44
**Unsure**	83<br>11.08<br>90.22<br>22.07	9<br>1.20<br>9.78<br>2.41	0<br>0.00<br>0.00<br>.	92<br>12.28
**Total**	376<br>50.20	373<br>49.80	0<br>0.00	749<br>100.00
Frequency Missing = 1				

Knowledge Question 2e correct				
know_q2e_correct	Frequency	Percent	Cumulative Frequency	Cumulative Percent
no	350	18.86	350	18.86
YES	1506	81.14	1856	100.00
Frequency Missing = 1				

Table 1 of know_q2e_correct by event				
Controlling for group=Control				
know_q2e_correct(Knowledge Question 2e correct)	event(Time)			
Frequency<br>Percent<br>Row Pct<br>Col Pct	Pre-survey	Post-survey	Re-test	Total
no	97<br>8.76<br>45.97<br>26.15	22<br>1.99<br>10.43<br>6.03	92<br>8.31<br>43.60<br>24.80	211<br>19.06
YES	274<br>24.75<br>30.58<br>73.85	343<br>30.98<br>38.28<br>93.97	279<br>25.20<br>31.14<br>75.20	896<br>80.94
Total	371<br>33.51	365<br>32.97	371<br>33.51	1107<br>100.00

Table 2 of know_q2e_correct by event				
Controlling for group=Experimental				
know_q2e_correct(Knowledge Question 2e correct)	event(Time)			
Frequency<br>Percent<br>Row Pct<br>Col Pct	Pre-survey	Post-survey	Re-test	Total
no	111<br>14.82<br>79.86<br>29.52	28<br>3.74<br>20.14<br>7.51	0<br>0.00<br>0.00<br>.	139<br>18.56
YES	265<br>35.38<br>43.44<br>70.48	345<br>46.06<br>56.56<br>92.49	0<br>0.00<br>0.00<br>.	610<br>81.44
Total	376<br>50.20	373<br>49.80	0<br>0.00	749<br>100.00
Frequency Missing = 1				

Financial penalty				
know_q3a	Frequency	Percent	Cumulative Frequency	Cumulative Percent
no	245	13.20	245	13.20
YES	1146	61.75	1391	74.95
Unsure	465	25.05	1856	100.00
Frequency Missing = 1				

Table 1 of know_q3a by event				
Controlling for group=Control				
know_q3a(Financial penalty)	event(Time)			
Frequency<br>Percent<br>Row Pct<br>Col Pct	Pre-survey	Post-survey	Re-test	Total
no	49<br>4.43<br>36.57<br>13.21	34<br>3.07<br>25.37<br>9.32	51<br>4.61<br>38.06<br>13.75	134<br>12.10
YES	165<br>14.91<br>25.27<br>44.47	316<br>28.55<br>48.39<br>86.58	172<br>15.54<br>26.34<br>46.36	653<br>58.99
Unsure	157<br>14.18<br>49.06<br>42.32	15<br>1.36<br>4.69<br>4.11	148<br>13.37<br>46.25<br>39.89	320<br>28.91
Total	371<br>33.51	365<br>32.97	371<br>33.51	1107<br>100.00

Table 2 of know_q3a by event				
Controlling for group=Experimental				
know_q3a(Financial penalty)	event(Time)			
Frequency<br>Percent<br>Row Pct<br>Col Pct	Pre-survey	Post-survey	Re-test	Total
no	64<br>8.54<br>57.66<br>17.02	47<br>6.28<br>42.34<br>12.60	0<br>0.00<br>0.00<br>.	111<br>14.82
YES	184<br>24.57<br>37.32<br>48.94	309<br>41.26<br>62.68<br>82.84	0<br>0.00<br>0.00<br>.	493<br>65.82
Unsure	128<br>17.09<br>88.28<br>34.04	17<br>2.27<br>11.72<br>4.56	0<br>0.00<br>0.00<br>.	145<br>19.36
Total	376<br>50.20	373<br>49.80	0<br>0.00	749<br>100.00
Frequency Missing = 1				

Knowledge Question 3a correct				
know_q3a_correct	Frequency	Percent	Cumulative Frequency	Cumulative Percent
no	710	38.25	710	38.25
YES	1146	61.75	1856	100.00
Frequency Missing = 1				

Table 1 of know_q3a_correct by event				
Controlling for group=Control				
know_q3a_correct(Knowledge Question 3a correct)	event(Time)			
Frequency<br>Percent<br>Row Pct<br>Col Pct	Pre-survey	Post-survey	Re-test	Total
no	206<br>18.61<br>45.37<br>55.53	49<br>4.43<br>10.79<br>13.42	199<br>17.98<br>43.83<br>53.64	454<br>41.01
YES	165<br>14.91<br>25.27<br>44.47	316<br>28.55<br>48.39<br>86.58	172<br>15.54<br>26.34<br>46.36	653<br>58.99
Total	371<br>33.51	365<br>32.97	371<br>33.51	1107<br>100.00

Table 2 of know_q3a_correct by event				
Controlling for group=Experimental				
know_q3a_correct(Knowledge Question 3a correct)	event(Time)			
Frequency<br>Percent<br>Row Pct<br>Col Pct	Pre-survey	Post-survey	Re-test	Total
no	192<br>25.63<br>75.00<br>51.06	64<br>8.54<br>25.00<br>17.16	0<br>0.00<br>0.00<br>.	256<br>34.18
YES	184<br>24.57<br>37.32<br>48.94	309<br>41.26<br>62.68<br>82.84	0<br>0.00<br>0.00<br>.	493<br>65.82
Total	376<br>50.20	373<br>49.80	0<br>0.00	749<br>100.00
Frequency Missing = 1				

Loss of professional license				
know_q3b	Frequency	Percent	Cumulative Frequency	Cumulative Percent
no	68	3.66	68	3.66
YES	1596	85.99	1664	89.66
Unsure	192	10.34	1856	100.00
Frequency Missing = 1				

Table 1 of know_q3b by event				
Controlling for group=Control				
know_q3b(Loss of professional license)	event(Time)			
Frequency<br>Percent<br>Row Pct<br>Col Pct	Pre-survey	Post-survey	Re-test	Total
no	11<br>0.99<br>27.50<br>2.96	14<br>1.26<br>35.00<br>3.84	15<br>1.36<br>37.50<br>4.04	40<br>3.61
YES	303<br>27.37<br>31.93<br>81.67	343<br>30.98<br>36.14<br>93.97	303<br>27.37<br>31.93<br>81.67	949<br>85.73
Unsure	57<br>5.15<br>48.31<br>15.36	8<br>0.72<br>6.78<br>2.19	53<br>4.79<br>44.92<br>14.29	118<br>10.66
Total	371<br>33.51	365<br>32.97	371<br>33.51	1107<br>100.00

Table 2 of know_q3b by event				
Controlling for group=Experimental				
know_q3b(Loss of professional license)	event(Time)			
Frequency<br>Percent<br>Row Pct<br>Col Pct	Pre-survey	Post-survey	Re-test	Total
no	8<br>1.07<br>28.57<br>2.13	20<br>2.67<br>71.43<br>5.36	0<br>0.00<br>0.00<br>.	28<br>3.74
YES	303<br>40.45<br>46.83<br>80.59	344<br>45.93<br>53.17<br>92.23	0<br>0.00<br>0.00<br>.	647<br>86.38
Unsure	65<br>8.68<br>87.84<br>17.29	9<br>1.20<br>12.16<br>2.41	0<br>0.00<br>0.00<br>.	74<br>9.88
Total	376<br>50.20	373<br>49.80	0<br>0.00	749<br>100.00
Frequency Missing = 1				

Knowledge Question 3b correct				
know_q3b_correct	Frequency	Percent	Cumulative Frequency	Cumulative Percent
no	260	14.01	260	14.01
YES	1596	85.99	1856	100.00
Frequency Missing = 1				

Table 1 of know_q3b_correct by event				
Controlling for group=Control				
know_q3b_correct(Knowledge Question 3b correct)	event(Time)			
Frequency<br>Percent<br>Row Pct<br>Col Pct	Pre-survey	Post-survey	Re-test	Total
no	68<br>6.14<br>43.04<br>18.33	22<br>1.99<br>13.92<br>6.03	68<br>6.14<br>43.04<br>18.33	158<br>14.27
YES	303<br>27.37<br>31.93<br>81.67	343<br>30.98<br>36.14<br>93.97	303<br>27.37<br>31.93<br>81.67	949<br>85.73
Total	371<br>33.51	365<br>32.97	371<br>33.51	1107<br>100.00

Table 2 of know_q3b_correct by event				
Controlling for group=Experimental				
know_q3b_correct(Knowledge Question 3b correct)	event(Time)			
Frequency<br>Percent<br>Row Pct<br>Col Pct	Pre-survey	Post-survey	Re-test	Total
no	73<br>9.75<br>71.57<br>19.41	29<br>3.87<br>28.43<br>7.77	0<br>0.00<br>0.00<br>.	102<br>13.62
YES	303<br>40.45<br>46.83<br>80.59	344<br>45.93<br>53.17<br>92.23	0<br>0.00<br>0.00<br>.	647<br>86.38
Total	376<br>50.20	373<br>49.80	0<br>0.00	749<br>100.00
Frequency Missing = 1				

Incarceration				
know_q3c	Frequency	Percent	Cumulative Frequency	Cumulative Percent
no	269	14.49	269	14.49
YES	1153	62.12	1422	76.62
Unsure	434	23.38	1856	100.00
Frequency Missing = 1				

Table 1 of know_q3c by event				
Controlling for group=Control				
know_q3c(Incarceration)	event(Time)			
Frequency<br>Percent<br>Row Pct<br>Col Pct	Pre-survey	Post-survey	Re-test	Total
no	62<br>5.60<br>38.75<br>16.71	34<br>3.07<br>21.25<br>9.32	64<br>5.78<br>40.00<br>17.25	160<br>14.45
YES	177<br>15.99<br>26.58<br>47.71	307<br>27.73<br>46.10<br>84.11	182<br>16.44<br>27.33<br>49.06	666<br>60.16
Unsure	132<br>11.92<br>46.98<br>35.58	24<br>2.17<br>8.54<br>6.58	125<br>11.29<br>44.48<br>33.69	281<br>25.38
Total	371<br>33.51	365<br>32.97	371<br>33.51	1107<br>100.00

Table 2 of know_q3c by event				
Controlling for group=Experimental				
know_q3c(Incarceration)	event(Time)			
Frequency<br>Percent<br>Row Pct<br>Col Pct	Pre-survey	Post-survey	Re-test	Total
**no**	69<br>9.21<br>63.30<br>18.35	40<br>5.34<br>36.70<br>10.72	0<br>0.00<br>0.00<br>.	109<br>14.55
**YES**	170<br>22.70<br>34.91<br>45.21	317<br>42.32<br>65.09<br>84.99	0<br>0.00<br>0.00<br>.	487<br>65.02
**Unsure**	137<br>18.29<br>89.54<br>36.44	16<br>2.14<br>10.46<br>4.29	0<br>0.00<br>0.00<br>.	153<br>20.43
**Total**	376<br>50.20	373<br>49.80	0<br>0.00	749<br>100.00
Frequency Missing = 1				

Knowledge Question 3c correct				
know_q3c_correct	Frequency	Percent	Cumulative Frequency	Cumulative Percent
no	703	37.88	703	37.88
YES	1153	62.12	1856	100.00
Frequency Missing = 1				

Table 1 of know_q3c_correct by event				
Controlling for group=Control				
know_q3c_correct(Knowledge Question 3c correct)	event(Time)			
Frequency<br>Percent<br>Row Pct<br>Col Pct	Pre-survey	Post-survey	Re-test	Total
no	194<br>17.52<br>43.99<br>52.29	58<br>5.24<br>13.15<br>15.89	189<br>17.07<br>42.86<br>50.94	441<br>39.84
YES	177<br>15.99<br>26.58<br>47.71	307<br>27.73<br>46.10<br>84.11	182<br>16.44<br>27.33<br>49.06	666<br>60.16
Total	371<br>33.51	365<br>32.97	371<br>33.51	1107<br>100.00

Table 2 of know_q3c_correct by event				
Controlling for group=Experimental				
know_q3c_correct(Knowledge Question 3c correct)	event(Time)			
Frequency<br>Percent<br>Row Pct<br>Col Pct	Pre-survey	Post-survey	Re-test	Total
no	206<br>27.50<br>78.63<br>54.79	56<br>7.48<br>21.37<br>15.01	0<br>0.00<br>0.00<br>.	262<br>34.98
YES	170<br>22.70<br>34.91<br>45.21	317<br>42.32<br>65.09<br>84.99	0<br>0.00<br>0.00<br>.	487<br>65.02
Total	376<br>50.20	373<br>49.80	0<br>0.00	749<br>100.00
Frequency Missing = 1				

Under recently enacted Pennsylvania law, for an act (or failure to act) to count as abuse/neglect, which of the following must be true?				
know_q4	Frequency	Percent	Cumulative Frequency	Cumulative Percent
Any of the above	756	40.73	756	40.73
All of the above	1073	57.81	1829	98.55
None of the above	27	1.45	1856	100.00
Frequency Missing = 1				

Table 1 of know_q4 by event				
Controlling for group=Control				
know_q4(Under recently enacted Pennsylvania law, for an act (or failure to act) to count as abuse/neglect, which of the following must be true?)	event(Time)			
Frequency<br>Percent<br>Row Pct<br>Col Pct	Pre-survey	Post-survey	Re-test	Total
Any of the above	148<br>13.37<br>31.62<br>39.89	167<br>15.09<br>35.68<br>45.75	153<br>13.82<br>32.69<br>41.24	468<br>42.28
All of the above	216<br>19.51<br>34.67<br>58.22	195<br>17.62<br>31.30<br>53.42	212<br>19.15<br>34.03<br>57.14	623<br>56.28
None of the above	7<br>0.63<br>43.75<br>1.89	3<br>0.27<br>18.75<br>0.82	6<br>0.54<br>37.50<br>1.62	16<br>1.45
Total	371<br>33.51	365<br>32.97	371<br>33.51	1107<br>100.00

Table 2 of know_q4 by event				
Controlling for group=Experimental				
know_q4(Under recently enacted Pennsylvania law, for an act (or failure to act) to count as abuse/neglect, which of the following must be true?)	event(Time)			
Frequency<br>Percent<br>Row Pct<br>Col Pct	Pre-survey	Post-survey	Re-test	Total
Any of the above	135<br>18.02<br>46.88<br>35.90	153<br>20.43<br>53.13<br>41.02	0<br>0.00<br>0.00<br>.	288<br>38.45
All of the above	235<br>31.38<br>52.22<br>62.50	215<br>28.70<br>47.78<br>57.64	0<br>0.00<br>0.00<br>.	450<br>60.08
None of the above	6<br>0.80<br>54.55<br>1.60	5<br>0.67<br>45.45<br>1.34	0<br>0.00<br>0.00<br>.	11<br>1.47
Total	376<br>50.20	373<br>49.80	0<br>0.00	749<br>100.00
Frequency Missing = 1				

Knowledge Question 4 correct				
know_q4_correct	Frequency	Percent	Cumulative Frequency	Cumulative Percent
no	1100	59.27	1100	59.27
YES	756	40.73	1856	100.00
Frequency Missing = 1				

Table 1 of know_q4_correct by event				
Controlling for group=Control				
know_q4_correct(Knowledge Question 4 correct)	event(Time)			
Frequency<br>Percent<br>Row Pct<br>Col Pct	Pre-survey	Post-survey	Re-test	Total
no	223<br>20.14<br>34.90<br>60.11	198<br>17.89<br>30.99<br>54.25	218<br>19.69<br>34.12<br>58.76	639<br>57.72
YES	148<br>13.37<br>31.62<br>39.89	167<br>15.09<br>35.68<br>45.75	153<br>13.82<br>32.69<br>41.24	468<br>42.28
Total	371<br>33.51	365<br>32.97	371<br>33.51	1107<br>100.00

Table 2 of know_q4_correct by event				
Controlling for group=Experimental				
know_q4_correct(Knowledge Question 4 correct)	event(Time)			
Frequency<br>Percent<br>Row Pct<br>Col Pct	Pre-survey	Post-survey	Re-test	Total
no	241<br>32.18<br>52.28<br>64.10	220<br>29.37<br>47.72<br>58.98	0<br>0.00<br>0.00<br>.	461<br>61.55
YES	135<br>18.02<br>46.88<br>35.90	153<br>20.43<br>53.13<br>41.02	0<br>0.00<br>0.00<br>.	288<br>38.45
Total	376<br>50.20	373<br>49.80	0<br>0.00	749<br>100.00
Frequency Missing = 1				

Under Pennsylvania law, you can be held legally liable if you suspect child abuse/neglect and report it, but it turns out to be unfounded.				
know_q5	Frequency	Percent	Cumulative Frequency	Cumulative Percent
True	205	11.05	205	11.05
False	1319	71.07	1524	82.11
I am unsure	332	17.89	1856	100.00
Frequency Missing = 1				

Table 1 of know_q5 by event				
Controlling for group=Control				
know_q5(Under Pennsylvania law, you can be held legally liable if you suspect child abuse/neglect and report it, but it turns out to be unfounded.)	event(Time)			
Frequency<br>Percent<br>Row Pct<br>Col Pct	Pre-survey	Post-survey	Re-test	Total
True	28<br>2.53<br>21.71<br>7.55	59<br>5.33<br>45.74<br>16.16	42<br>3.79<br>32.56<br>11.32	129<br>11.65
False	236<br>21.32<br>30.93<br>63.61	294<br>26.56<br>38.53<br>80.55	233<br>21.05<br>30.54<br>62.80	763<br>68.93
I am unsure	107<br>9.67<br>49.77<br>28.84	12<br>1.08<br>5.58<br>3.29	96<br>8.67<br>44.65<br>25.88	215<br>19.42
Total	371<br>33.51	365<br>32.97	371<br>33.51	1107<br>100.00

Table 2 of know_q5 by event				
Controlling for group=Experimental				
know_q5(Under Pennsylvania law, you can be held legally liable if you suspect child abuse/neglect and report it, but it turns out to be unfounded.)	event(Time)			
Frequency<br>Percent<br>Row Pct<br>Col Pct	Pre-survey	Post-survey	Re-test	Total
True	28<br>3.74<br>36.84<br>7.45	48<br>6.41<br>63.16<br>12.87	0<br>0.00<br>0.00<br>.	76<br>10.15
False	242<br>32.31<br>43.53<br>64.36	314<br>41.92<br>56.47<br>84.18	0<br>0.00<br>0.00<br>.	556<br>74.23
I am unsure	106<br>14.15<br>90.60<br>28.19	11<br>1.47<br>9.40<br>2.95	0<br>0.00<br>0.00<br>.	117<br>15.62
Total	376<br>50.20	373<br>49.80	0<br>0.00	749<br>100.00
Frequency Missing = 1				

Knowledge Question 5 correct				
know_q5_correct	Frequency	Percent	Cumulative Frequency	Cumulative Percent
no	537	28.93	537	28.93
YES	1319	71.07	1856	100.00
Frequency Missing = 1				

Table 1 of know_q5_correct by event				
Controlling for group=Control				
know_q5_correct(Knowledge Question 5 correct)	event(Time)			
Frequency<br>Percent<br>Row Pct<br>Col Pct	Pre-survey	Post-survey	Re-test	Total
no	135<br>12.20<br>39.24<br>36.39	71<br>6.41<br>20.64<br>19.45	138<br>12.47<br>40.12<br>37.20	344<br>31.07
YES	236<br>21.32<br>30.93<br>63.61	294<br>26.56<br>38.53<br>80.55	233<br>21.05<br>30.54<br>62.80	763<br>68.93
Total	371<br>33.51	365<br>32.97	371<br>33.51	1107<br>100.00

Table 2 of know_q5_correct by event				
Controlling for group=Experimental				
know_q5_correct(Knowledge Question 5 correct)	event(Time)			
Frequency<br>Percent<br>Row Pct<br>Col Pct	Pre-survey	Post-survey	Re-test	Total
no	134<br>17.89<br>69.43<br>35.64	59<br>7.88<br>30.57<br>15.82	0<br>0.00<br>0.00<br>.	193<br>25.77
YES	242<br>32.31<br>43.53<br>64.36	314<br>41.92<br>56.47<br>84.18	0<br>0.00<br>0.00<br>.	556<br>74.23
Total	376<br>50.20	373<br>49.80	0<br>0.00	749<br>100.00
Frequency Missing = 1				

Under Pennsylvania law, once you have reasonable cause to suspect child abuse/neglect, you must report your suspicion to authorities:				
know_q6	Frequency	Percent	Cumulative Frequency	Cumulative Percent
Within a day	269	14.49	269	14.49
Within 48 hours	248	13.36	517	27.86
Within 72 hours	34	1.83	551	29.69
Immediately	1093	58.89	1644	88.58
Within a week	9	0.48	1653	89.06
I am unsure	203	10.94	1856	100.00
Frequency Missing = 1				

Table 1 of know_q6 by event				
Controlling for group=Control				
know_q6(Under Pennsylvania law, once you have reasonable cause to suspect child abuse/neglect, you must report your suspicion to authorities:)	event(Time)			
Frequency<br>Percent<br>Row Pct<br>Col Pct	Pre-survey	Post-survey	Re-test	Total
Within a day	53<br>4.79<br>31.93<br>14.29	49<br>4.43<br>29.52<br>13.42	64<br>5.78<br>38.55<br>17.25	166<br>15.00
Within 48 hours	64<br>5.78<br>42.38<br>17.25	26<br>2.35<br>17.22<br>7.12	61<br>5.51<br>40.40<br>16.44	151<br>13.64
Within 72 hours	5<br>0.45<br>29.41<br>1.35	5<br>0.45<br>29.41<br>1.37	7<br>0.63<br>41.18<br>1.89	17<br>1.54
Immediately	196<br>17.71<br>31.41<br>52.83	273<br>24.66<br>43.75<br>74.79	155<br>14.00<br>24.84<br>41.78	624<br>56.37
Within a week	0<br>0.00<br>0.00<br>0.00	4<br>0.36<br>80.00<br>1.10	1<br>0.09<br>20.00<br>0.27	5<br>0.45
I am unsure	53<br>4.79<br>36.81<br>14.29	8<br>0.72<br>5.56<br>2.19	83<br>7.50<br>57.64<br>22.37	144<br>13.01
Total	371<br>33.51	365<br>32.97	371<br>33.51	1107<br>100.00

Table 2 of know_q6 by event				
Controlling for group=Experimental				
know_q6(Under Pennsylvania law, once you have reasonable cause to suspect child abuse/neglect, you must report your suspicion to authorities:)	event(Time)			
Frequency<br>Percent<br>Row Pct<br>Col Pct	Pre-survey	Post-survey	Re-test	Total
Within a day	48<br>6.41<br>46.60<br>12.77	55<br>7.34<br>53.40<br>14.75	0<br>0.00<br>0.00<br>.	103<br>13.75
Within 48 hours	57<br>7.61<br>58.76<br>15.16	40<br>5.34<br>41.24<br>10.72	0<br>0.00<br>0.00<br>.	97<br>12.95
Within 72 hours	14<br>1.87<br>82.35<br>3.72	3<br>0.40<br>17.65<br>0.80	0<br>0.00<br>0.00<br>.	17<br>2.27
Immediately	205<br>27.37<br>43.71<br>54.52	264<br>35.25<br>56.29<br>70.78	0<br>0.00<br>0.00<br>.	469<br>62.62
Within a week	2<br>0.27<br>50.00<br>0.53	2<br>0.27<br>50.00<br>0.54	0<br>0.00<br>0.00<br>.	4<br>0.53
I am unsure	50<br>6.68<br>84.75<br>13.30	9<br>1.20<br>15.25<br>2.41	0<br>0.00<br>0.00<br>.	59<br>7.88
Total	376<br>50.20	373<br>49.80	0<br>0.00	749<br>100.00
Frequency Missing = 1				

Knowledge Question 6 correct				
know_q6_correct	Frequency	Percent	Cumulative Frequency	Cumulative Percent
no	763	41.11	763	41.11
YES	1093	58.89	1856	100.00
Frequency Missing = 1				

Table 1 of know_q6_correct by event				
Controlling for group=Control				
know_q6_correct(Knowledge Question 6 correct)	event(Time)			
Frequency<br>Percent<br>Row Pct<br>Col Pct	Pre-survey	Post-survey	Re-test	Total
no	175<br>15.81<br>36.23<br>47.17	92<br>8.31<br>19.05<br>25.21	216<br>19.51<br>44.72<br>58.22	483<br>43.63
YES	196<br>17.71<br>31.41<br>52.83	273<br>24.66<br>43.75<br>74.79	155<br>14.00<br>24.84<br>41.78	624<br>56.37
Total	371<br>33.51	365<br>32.97	371<br>33.51	1107<br>100.00

Table 2 of know_q6_correct by event				
Controlling for group=Experimental				
know_q6_correct(Knowledge Question 6 correct)	event(Time)			
Frequency<br>Percent<br>Row Pct<br>Col Pct	Pre-survey	Post-survey	Re-test	Total
no	171<br>22.83<br>61.07<br>45.48	109<br>14.55<br>38.93<br>29.22	0<br>0.00<br>0.00<br>.	280<br>37.38
YES	205<br>27.37<br>43.71<br>54.52	264<br>35.25<br>56.29<br>70.78	0<br>0.00<br>0.00<br>.	469<br>62.62
Total	376<br>50.20	373<br>49.80	0<br>0.00	749<br>100.00
Frequency Missing = 1				

Under Pennsylvania law, you must report:				
**know_q7**	**Frequency**	**Percent**	**Cumulative Frequency**	**Cumulative Percent**
**Whenever I think it is possible that a child has been abused/neglected**	247	13.31	247	13.31
**Only when I am certain that a child has been abused/neglected**	76	4.09	323	17.40
**Whenever I have reasonable cause to suspect that a child has been abused/neglected**	1472	79.31	1795	96.71
**Only if I think it is more likely than not that a child has been abused/neglected**	18	0.97	1813	97.68
**I am unsure**	43	2.32	1856	100.00
**Frequency Missing = 1**				

Table 1 of know_q7 by event				
**Controlling for group=Control**				
**know_q7(Under Pennsylvania law, you must report:)**	**event(Time)**			
**Frequency<br>Percent<br>Row Pct<br>Col Pct**	**Pre-survey**	**Post-survey**	**Re-test**	**Total**
**Whenever I think it is possible that a child has been abused/neglected**	55<br>4.97<br>37.67<br>14.82	27<br>2.44<br>18.49<br>7.40	64<br>5.78<br>43.84<br>17.25	146<br>13.19
**Only when I am certain that a child has been abused/neglected**	21<br>1.90<br>42.00<br>5.66	8<br>0.72<br>16.00<br>2.19	21<br>1.90<br>42.00<br>5.66	50<br>4.52
**Whenever I have reasonable cause to suspect that a child has been abused/neglected**	273<br>24.66<br>31.24<br>73.58	327<br>29.54<br>37.41<br>89.59	274<br>24.75<br>31.35<br>73.85	874<br>78.95
**Only if I think it is more likely than not that a child has been abused/neglected**	2<br>0.18<br>33.33<br>0.54	2<br>0.18<br>33.33<br>0.55	2<br>0.18<br>33.33<br>0.54	6<br>0.54
**I am unsure**	20<br>1.81<br>64.52<br>5.39	1<br>0.09<br>3.23<br>0.27	10<br>0.90<br>32.26<br>2.70	31<br>2.80
**Total**	371<br>33.51	365<br>32.97	371<br>33.51	1107<br>100.00

Table 2 of know_q7 by event				
Controlling for group=Experimental				
know_q7(Under Pennsylvania law, you must report:)	event(Time)			
Frequency<br>Percent<br>Row Pct<br>Col Pct	Pre-survey	Post-survey	Re-test	Total
Whenever I think it is possible that a child has been abused/neglected	57<br>7.61<br>56.44<br>15.16	44<br>5.87<br>43.56<br>11.80	0<br>0.00<br>0.00<br>.	101<br>13.48
Only when I am certain that a child has been abused/neglected	18<br>2.40<br>69.23<br>4.79	8<br>1.07<br>30.77<br>2.14	0<br>0.00<br>0.00<br>.	26<br>3.47
Whenever I have reasonable cause to suspect that a child has been abused/neglected	283<br>37.78<br>47.32<br>75.27	315<br>42.06<br>52.68<br>84.45	0<br>0.00<br>0.00<br>.	598<br>79.84
Only if I think it is more likely than not that a child has been abused/neglected	6<br>0.80<br>50.00<br>1.60	6<br>0.80<br>50.00<br>1.61	0<br>0.00<br>0.00<br>.	12<br>1.60
I am unsure	12<br>1.60<br>100.00<br>3.19	0<br>0.00<br>0.00<br>0.00	0<br>0.00<br>0.00<br>.	12<br>1.60
Total	376<br>50.20	373<br>49.80	0<br>0.00	749<br>100.00
Frequency Missing = 1				

Knowledge Question 7 correct				
know_q7_correct	Frequency	Percent	Cumulative Frequency	Cumulative Percent
no	384	20.69	384	20.69
YES	1472	79.31	1856	100.00
Frequency Missing = 1				

Table 1 of know_q7_correct by event				
Controlling for group=Control				
know_q7_correct(Knowledge Question 7 correct)	event(Time)			
Frequency<br>Percent<br>Row Pct<br>Col Pct	Pre-survey	Post-survey	Re-test	Total
no	98<br>8.85<br>42.06<br>26.42	38<br>3.43<br>16.31<br>10.41	97<br>8.76<br>41.63<br>26.15	233<br>21.05
YES	273<br>24.66<br>31.24<br>73.58	327<br>29.54<br>37.41<br>89.59	274<br>24.75<br>31.35<br>73.85	874<br>78.95
Total	371<br>33.51	365<br>32.97	371<br>33.51	1107<br>100.00

Table 2 of know_q7_correct by event				
Controlling for group=Experimental				
know_q7_correct(Knowledge Question 7 correct)	event(Time)			
Frequency<br>Percent<br>Row Pct<br>Col Pct	Pre-survey	Post-survey	Re-test	Total
no	93<br>12.42<br>61.59<br>24.73	58<br>7.74<br>38.41<br>15.55	0<br>0.00<br>0.00<br>.	151<br>20.16
YES	283<br>37.78<br>47.32<br>75.27	315<br>42.06<br>52.68<br>84.45	0<br>0.00<br>0.00<br>.	598<br>79.84
Total	376<br>50.20	373<br>49.80	0<br>0.00	749<br>100.00
Frequency Missing = 1				

According to newly enacted Pennsylvania law, you are required to report suspected child abuse/neglect to:				
**know_q8**	**Frequency**	**Percent**	**Cumulative Frequency**	**Cumulative Percent**
**The police**	108	5.82	108	5.82
**ChildLine only**	576	31.03	684	36.85
**My supervisor only**	55	2.96	739	39.82
**Both ChildLine and my supervisor**	981	52.86	1720	92.67
**I am unsure**	136	7.33	1856	100.00
**Frequency Missing = 1**				

Table 1 of know_q8 by event				
**Controlling for group=Control**				
**know_q8(According to newly enacted Pennsylvania law, you are required to report suspected child abuse/neglect to:)**	**event(Time)**			
**Frequency<br>Percent<br>Row Pct<br>Col Pct**	**Pre-survey**	**Post-survey**	**Re-test**	**Total**
**The police**	29<br>2.62<br>40.28<br>7.82	13<br>1.17<br>18.06<br>3.56	30<br>2.71<br>41.67<br>8.09	72<br>6.50
**ChildLine only**	115<br>10.39<br>31.51<br>31.00	135<br>12.20<br>36.99<br>36.99	115<br>10.39<br>31.51<br>31.00	365<br>32.97
**My supervisor only**	15<br>1.36<br>45.45<br>4.04	5<br>0.45<br>15.15<br>1.37	13<br>1.17<br>39.39<br>3.50	33<br>2.98
**Both ChildLine and my supervisor**	169<br>15.27<br>30.90<br>45.55	207<br>18.70<br>37.84<br>56.71	171<br>15.45<br>31.26<br>46.09	547<br>49.41
**I am unsure**	43<br>3.88<br>47.78<br>11.59	5<br>0.45<br>5.56<br>1.37	42<br>3.79<br>46.67<br>11.32	90<br>8.13
**Total**	371<br>33.51	365<br>32.97	371<br>33.51	1107<br>100.00

Table 2 of know_q8 by event				
Controlling for group=Experimental				
know_q8(According to newly enacted Pennsylvania law, you are required to report suspected child abuse/neglect to:)	event(Time)			
Frequency<br>Percent<br>Row Pct<br>Col Pct	Pre-survey	Post-survey	Re-test	Total
The police	25<br>3.34<br>69.44<br>6.65	11<br>1.47<br>30.56<br>2.95	0<br>0.00<br>0.00<br>.	36<br>4.81
ChildLine only	85<br>11.35<br>40.28<br>22.61	126<br>16.82<br>59.72<br>33.78	0<br>0.00<br>0.00<br>.	211<br>28.17
My supervisor only	19<br>2.54<br>86.36<br>5.05	3<br>0.40<br>13.64<br>0.80	0<br>0.00<br>0.00<br>.	22<br>2.94
Both ChildLine and my supervisor	203<br>27.10<br>46.77<br>53.99	231<br>30.84<br>53.23<br>61.93	0<br>0.00<br>0.00<br>.	434<br>57.94
I am unsure	44<br>5.87<br>95.65<br>11.70	2<br>0.27<br>4.35<br>0.54	0<br>0.00<br>0.00<br>.	46<br>6.14
Total	376<br>50.20	373<br>49.80	0<br>0.00	749<br>100.00
Frequency Missing = 1				

Knowledge Question 8 correct				
know_q8_correct	Frequency	Percent	Cumulative Frequency	Cumulative Percent
no	875	47.14	875	47.14
YES	981	52.86	1856	100.00
Frequency Missing = 1				

Table 1 of know_q8_correct by event				
Controlling for group=Control				
know_q8_correct(Knowledge Question 8 correct)	event(Time)			
Frequency<br>Percent<br>Row Pct<br>Col Pct	Pre-survey	Post-survey	Re-test	Total
no	202<br>18.25<br>36.07<br>54.45	158<br>14.27<br>28.21<br>43.29	200<br>18.07<br>35.71<br>53.91	560<br>50.59
YES	169<br>15.27<br>30.90<br>45.55	207<br>18.70<br>37.84<br>56.71	171<br>15.45<br>31.26<br>46.09	547<br>49.41
Total	371<br>33.51	365<br>32.97	371<br>33.51	1107<br>100.00

Table 2 of know_q8_correct by event				
Controlling for group=Experimental				
know_q8_correct(Knowledge Question 8 correct)	event(Time)			
Frequency<br>Percent<br>Row Pct<br>Col Pct	Pre-survey	Post-survey	Re-test	Total
no	173<br>23.10<br>54.92<br>46.01	142<br>18.96<br>45.08<br>38.07	0<br>0.00<br>0.00<br>.	315<br>42.06
YES	203<br>27.10<br>46.77<br>53.99	231<br>30.84<br>53.23<br>61.93	0<br>0.00<br>0.00<br>.	434<br>57.94
Total	376<br>50.20	373<br>49.80	0<br>0.00	749<br>100.00
Frequency Missing = 1				

To count as physical child abuse, it must result in a child experiencing:				
know_q9	Frequency	Percent	Cumulative Frequency	Cumulative Percent
Severe pain	37	4.95	37	4.95
Any pain	407	54.48	444	59.44
Substantial pain	119	15.93	563	75.37
Long-lasting pain	43	5.76	606	81.12
I am unsure	141	18.88	747	100.00
Frequency Missing = 1110				

Table 1 of know_q9 by event				
Controlling for group=Control				
know_q9(To count as physical child abuse, it must result in a child experiencing:)	event(Time)			
Frequency<br>Percent<br>Row Pct<br>Col Pct	Pre-survey	Post-survey	Re-test	Total
Severe pain	10<br>2.70<br>100.00<br>2.70	0<br>0.00<br>0.00<br>.	0<br>0.00<br>0.00<br>.	10<br>2.70
Any pain	203<br>54.72<br>100.00<br>54.72	0<br>0.00<br>0.00<br>.	0<br>0.00<br>0.00<br>.	203<br>54.72
Substantial pain	62<br>16.71<br>100.00<br>16.71	0<br>0.00<br>0.00<br>.	0<br>0.00<br>0.00<br>.	62<br>16.71
Long-lasting pain	25<br>6.74<br>100.00<br>6.74	0<br>0.00<br>0.00<br>.	0<br>0.00<br>0.00<br>.	25<br>6.74
I am unsure	71<br>19.14<br>100.00<br>19.14	0<br>0.00<br>0.00<br>.	0<br>0.00<br>0.00<br>.	71<br>19.14
Total	371<br>100.00	0<br>0.00	0<br>0.00	371<br>100.00
Frequency Missing = 736				

Table 2 of know_q9 by event				
Controlling for group=Experimental				
know_q9(To count as physical child abuse, it must result in a child experiencing:)	event(Time)			
Frequency<br>Percent<br>Row Pct<br>Col Pct	Pre-survey	Post-survey	Re-test	Total
Severe pain	27<br>7.18<br>100.00<br>7.18	0<br>0.00<br>0.00<br>.	0<br>0.00<br>0.00<br>.	27<br>7.18
Any pain	204<br>54.26<br>100.00<br>54.26	0<br>0.00<br>0.00<br>.	0<br>0.00<br>0.00<br>.	204<br>54.26
Substantial pain	57<br>15.16<br>100.00<br>15.16	0<br>0.00<br>0.00<br>.	0<br>0.00<br>0.00<br>.	57<br>15.16
Long-lasting pain	18<br>4.79<br>100.00<br>4.79	0<br>0.00<br>0.00<br>.	0<br>0.00<br>0.00<br>.	18<br>4.79
I am unsure	70<br>18.62<br>100.00<br>18.62	0<br>0.00<br>0.00<br>.	0<br>0.00<br>0.00<br>.	70<br>18.62
Total	376<br>100.00	0<br>0.00	0<br>0.00	376<br>100.00
Frequency Missing = 374				

Knowledge Question 9 correct				
know_q9_correct	Frequency	Percent	Cumulative Frequency	Cumulative Percent
no	628	84.07	628	84.07
YES	119	15.93	747	100.00
Frequency Missing = 1110				

Table 1 of know_q9_correct by event				
Controlling for group=Control				
know_q9_correct(Knowledge Question 9 correct)	event(Time)			
Frequency<br>Percent<br>Row Pct<br>Col Pct	Pre-survey	Post-survey	Re-test	Total
no	309<br>83.29<br>100.00<br>83.29	0<br>0.00<br>0.00<br>.	0<br>0.00<br>0.00<br>.	309<br>83.29
YES	62<br>16.71<br>100.00<br>16.71	0<br>0.00<br>0.00<br>.	0<br>0.00<br>0.00<br>.	62<br>16.71
Total	371<br>100.00	0<br>0.00	0<br>0.00	371<br>100.00
Frequency Missing = 736				

Table 2 of know_q9_correct by event				
Controlling for group=Experimental				
know_q9_correct(Knowledge Question 9 correct)	event(Time)			
Frequency<br>Percent<br>Row Pct<br>Col Pct	Pre-survey	Post-survey	Re-test	Total
no	319<br>84.84<br>100.00<br>84.84	0<br>0.00<br>0.00<br>.	0<br>0.00<br>0.00<br>.	319<br>84.84
YES	57<br>15.16<br>100.00<br>15.16	0<br>0.00<br>0.00<br>.	0<br>0.00<br>0.00<br>.	57<br>15.16
Total	376<br>100.00	0<br>0.00	0<br>0.00	376<br>100.00
Frequency Missing = 374				

Under Pennsylvania law, are you required to report suspected child abuse/neglect if a child was put at significant risk for being injured even when no injury or harm actually occurred?				
know_q10	Frequency	Percent	Cumulative Frequency	Cumulative Percent
no	54	2.91	54	2.91
YES	1538	82.87	1592	85.78
Unsure	264	14.22	1856	100.00
Frequency Missing = 1				

Table 1 of know_q10 by event				
Controlling for group=Control				
know_q10(Under Pennsylvania law, are you required to report suspected child abuse/neglect if a child was put at significant risk for being injured even when no injury or harm actually occurred?)	event(Time)			
Frequency<br>Percent<br>Row Pct<br>Col Pct	Pre-survey	Post-survey	Re-test	Total
no	23<br>2.08<br>58.97<br>6.20	9<br>0.81<br>23.08<br>2.47	7<br>0.63<br>17.95<br>1.89	39<br>3.52
YES	264<br>23.85<br>29.50<br>71.16	349<br>31.53<br>38.99<br>95.62	282<br>25.47<br>31.51<br>76.01	895<br>80.85
Unsure	84<br>7.59<br>48.55<br>22.64	7<br>0.63<br>4.05<br>1.92	82<br>7.41<br>47.40<br>22.10	173<br>15.63
Total	371<br>33.51	365<br>32.97	371<br>33.51	1107<br>100.00

Table 2 of know_q10 by event				
Controlling for group=Experimental				
know_q10(Under Pennsylvania law, are you required to report suspected child abuse/neglect if a child was put at significant risk for being injured even when no injury or harm actually occurred?)	event(Time)			
Frequency<br>Percent<br>Row Pct<br>Col Pct	Pre-survey	Post-survey	Re-test	Total
no	10<br>1.34<br>66.67<br>2.66	5<br>0.67<br>33.33<br>1.34	0<br>0.00<br>0.00<br>.	15<br>2.00
YES	278<br>37.12<br>43.23<br>73.94	365<br>48.73<br>56.77<br>97.86	0<br>0.00<br>0.00<br>.	643<br>85.85
Unsure	88<br>11.75<br>96.70<br>23.40	3<br>0.40<br>3.30<br>0.80	0<br>0.00<br>0.00<br>.	91<br>12.15
Total	376<br>50.20	373<br>49.80	0<br>0.00	749<br>100.00
Frequency Missing = 1				

Knowledge Question 10 correct				
know_q10_correct	Frequency	Percent	Cumulative Frequency	Cumulative Percent
no	318	17.13	318	17.13
YES	1538	82.87	1856	100.00
Frequency Missing = 1				

Table 1 of know_q10_correct by event				
Controlling for group=Control				
know_q10_correct(Knowledge Question 10 correct)	event(Time)			
Frequency<br>Percent<br>Row Pct<br>Col Pct	Pre-survey	Post-survey	Re-test	Total
no	107<br>9.67<br>50.47<br>28.84	16<br>1.45<br>7.55<br>4.38	89<br>8.04<br>41.98<br>23.99	212<br>19.15
YES	264<br>23.85<br>29.50<br>71.16	349<br>31.53<br>38.99<br>95.62	282<br>25.47<br>31.51<br>76.01	895<br>80.85
Total	371<br>33.51	365<br>32.97	371<br>33.51	1107<br>100.00

Table 2 of know_q10_correct by event				
Controlling for group=Experimental				
know_q10_correct(Knowledge Question 10 correct)	event(Time)			
Frequency<br>Percent<br>Row Pct<br>Col Pct	Pre-survey	Post-survey	Re-test	Total
no	98<br>13.08<br>92.45<br>26.06	8<br>1.07<br>7.55<br>2.14	0<br>0.00<br>0.00<br>.	106<br>14.15
YES	278<br>37.12<br>43.23<br>73.94	365<br>48.73<br>56.77<br>97.86	0<br>0.00<br>0.00<br>.	643<br>85.85
Total	376<br>50.20	373<br>49.80	0<br>0.00	749<br>100.00
Frequency Missing = 1				

Requirements for reporting suspected child abuse/neglect refer to recent events. How long ago counts as recent under Pennsylvania law?				
know_q11	Frequency	Percent	Cumulative Frequency	Cumulative Percent
1 day	95	12.72	95	12.72
1 week	90	12.05	185	24.77
1 month	81	10.84	266	35.61
1 year	41	5.49	307	41.10
2 years	54	7.23	361	48.33
I am unsure	386	51.67	747	100.00
Frequency Missing = 1110				

Table 1 of know_q11 by event				
Controlling for group=Control				
know_q11(Requirements for reporting suspected child abuse/neglect refer to recent events. How long ago counts as recent under Pennsylvania law?)	event(Time)			
Frequency<br>Percent<br>Row Pct<br>Col Pct	Pre-survey	Post-survey	Re-test	Total
1 day	43<br>11.59<br>100.00<br>11.59	0<br>0.00<br>0.00<br>.	0<br>0.00<br>0.00<br>.	43<br>11.59
1 week	39<br>10.51<br>100.00<br>10.51	0<br>0.00<br>0.00<br>.	0<br>0.00<br>0.00<br>.	39<br>10.51
1 month	38<br>10.24<br>100.00<br>10.24	0<br>0.00<br>0.00<br>.	0<br>0.00<br>0.00<br>.	38<br>10.24
1 year	22<br>5.93<br>100.00<br>5.93	0<br>0.00<br>0.00<br>.	0<br>0.00<br>0.00<br>.	22<br>5.93
2 years	25<br>6.74<br>100.00<br>6.74	0<br>0.00<br>0.00<br>.	0<br>0.00<br>0.00<br>.	25<br>6.74
I am unsure	204<br>54.99<br>100.00<br>54.99	0<br>0.00<br>0.00<br>.	0<br>0.00<br>0.00<br>.	204<br>54.99
Total	371<br>100.00	0<br>0.00	0<br>0.00	371<br>100.00
Frequency Missing = 736				

Table 2 of know_q11 by event				
Controlling for group=Experimental				
know_q11(Requirements for reporting suspected child abuse/neglect refer to recent events. How long ago counts as recent under Pennsylvania law?)	event(Time)			
Frequency<br>Percent<br>Row Pct<br>Col Pct	Pre-survey	Post-survey	Re-test	Total
1 day	52<br>13.83<br>100.00<br>13.83	0<br>0.00<br>0.00<br>.	0<br>0.00<br>0.00<br>.	52<br>13.83
1 week	51<br>13.56<br>100.00<br>13.56	0<br>0.00<br>0.00<br>.	0<br>0.00<br>0.00<br>.	51<br>13.56
1 month	43<br>11.44<br>100.00<br>11.44	0<br>0.00<br>0.00<br>.	0<br>0.00<br>0.00<br>.	43<br>11.44
1 year	19<br>5.05<br>100.00<br>5.05	0<br>0.00<br>0.00<br>.	0<br>0.00<br>0.00<br>.	19<br>5.05
2 years	29<br>7.71<br>100.00<br>7.71	0<br>0.00<br>0.00<br>.	0<br>0.00<br>0.00<br>.	29<br>7.71
I am unsure	182<br>48.40<br>100.00<br>48.40	0<br>0.00<br>0.00<br>.	0<br>0.00<br>0.00<br>.	182<br>48.40
Total	376<br>100.00	0<br>0.00	0<br>0.00	376<br>100.00
Frequency Missing = 374				

Knowledge Question 11 correct				
know_q11_correct	Frequency	Percent	Cumulative Frequency	Cumulative Percent
no	693	92.77	693	92.77
YES	54	7.23	747	100.00
Frequency Missing = 1110				

Table 1 of know_q11_correct by event				
Controlling for group=Control				
know_q11_correct(Knowledge Question 11 correct)	event(Time)			
Frequency<br>Percent<br>Row Pct<br>Col Pct	Pre-survey	Post-survey	Re-test	Total
no	346<br>93.26<br>100.00<br>93.26	0<br>0.00<br>0.00<br>.	0<br>0.00<br>0.00<br>.	346<br>93.26
YES	25<br>6.74<br>100.00<br>6.74	0<br>0.00<br>0.00<br>.	0<br>0.00<br>0.00<br>.	25<br>6.74
Total	371<br>100.00	0<br>0.00	0<br>0.00	371<br>100.00
Frequency Missing = 736				

Table 2 of know_q11_correct by event				
Controlling for group=Experimental				
know_q11_correct(Knowledge Question 11 correct)	event(Time)			
Frequency<br>Percent<br>Row Pct<br>Col Pct	Pre-survey	Post-survey	Re-test	Total
no	347<br>92.29<br>100.00<br>92.29	0<br>0.00<br>0.00<br>.	0<br>0.00<br>0.00<br>.	347<br>92.29
YES	29<br>7.71<br>100.00<br>7.71	0<br>0.00<br>0.00<br>.	0<br>0.00<br>0.00<br>.	29<br>7.71
Total	376<br>100.00	0<br>0.00	0<br>0.00	376<br>100.00
Frequency Missing = 374				

Do you feel confident in your ability to identify signs of child abuse/neglect?				
know_q12	Frequency	Percent	Cumulative Frequency	Cumulative Percent
no	60	5.41	60	5.41
YES	1049	94.59	1109	100.00
Frequency Missing = 748				

Table 1 of know_q12 by event				
Controlling for group=Control				
know_q12(Do you feel confident in your ability to identify signs of child abuse/neglect?)	event(Time)			
Frequency<br>Percent<br>Row Pct<br>Col Pct	Pre-survey	Post-survey	Re-test	Total
no	0<br>0.00<br>0.00<br>.	3<br>0.41<br>5.08<br>0.82	56<br>7.61<br>94.92<br>15.09	59<br>8.02
YES	0<br>0.00<br>0.00<br>.	362<br>49.18<br>53.47<br>99.18	315<br>42.80<br>46.53<br>84.91	677<br>91.98
Total	0<br>0.00	365<br>49.59	371<br>50.41	736<br>100.00
Frequency Missing = 371				

Table 2 of know_q12 by event				
Controlling for group=Experimental				
know_q12(Do you feel confident in your ability to identify signs of child abuse/neglect?)	event(Time)			
Frequency<br>Percent<br>Row Pct<br>Col Pct	Pre-survey	Post-survey	Re-test	Total
no	0<br>0.00<br>0.00<br>.	1<br>0.27<br>100.00<br>0.27	0<br>0.00<br>0.00<br>.	1<br>0.27
YES	0<br>0.00<br>0.00<br>.	372<br>99.73<br>100.00<br>99.73	0<br>0.00<br>0.00<br>.	372<br>99.73
Total	0<br>0.00	373<br>100.00	0<br>0.00	373<br>100.00
Frequency Missing = 377				

How prepared do you feel to report child abuse/neglect should the need arise?				
**know_q13**	**Frequency**	**Percent**	**Cumulative Frequency**	**Cumulative Percent**
**Entirely unprepared**	12	1.08	12	1.08
**Unprepared**	11	0.99	23	2.07
**Somewhat unprepared**	22	1.98	45	4.06
**Neither prepared nor unprepared**	89	8.03	134	12.08
**Somewhat prepared**	146	13.17	280	25.25
**Prepared**	394	35.53	674	60.78
**Very well prepared**	435	39.22	1109	100.00
**Frequency Missing = 748**				

Table 1 of know_q13 by event				
Controlling for group=Control				
know_q13(How prepared do you feel to report child abuse/neglect should the need arise?)	event(Time)			
Frequency<br>Percent<br>Row Pct<br>Col Pct	Pre-survey	Post-survey	Re-test	Total
Entirely unprepared	0<br>0.00<br>0.00<br>.	5<br>0.68<br>41.67<br>1.37	7<br>0.95<br>58.33<br>1.89	12<br>1.63
Unprepared	0<br>0.00<br>0.00<br>.	0<br>0.00<br>0.00<br>0.00	10<br>1.36<br>100.00<br>2.70	10<br>1.36
Somewhat unprepared	0<br>0.00<br>0.00<br>.	0<br>0.00<br>0.00<br>0.00	21<br>2.85<br>100.00<br>5.66	21<br>2.85
Neither prepared nor unprepared	0<br>0.00<br>0.00<br>.	10<br>1.36<br>12.66<br>2.74	69<br>9.38<br>87.34<br>18.60	79<br>10.73
Somewhat prepared	0<br>0.00<br>0.00<br>.	29<br>3.94<br>28.43<br>7.95	73<br>9.92<br>71.57<br>19.68	102<br>13.86
Prepared	0<br>0.00<br>0.00<br>.	122<br>16.58<br>52.59<br>33.42	110<br>14.95<br>47.41<br>29.65	232<br>31.52
Very well prepared	0<br>0.00<br>0.00<br>.	199<br>27.04<br>71.07<br>54.52	81<br>11.01<br>28.93<br>21.83	280<br>38.04
Total	0<br>0.00	365<br>49.59	371<br>50.41	736<br>100.00
Frequency Missing = 371				

Table 2 of know_q13 by event				
Controlling for group=Experimental				
know_q13(How prepared do you feel to report child abuse/neglect should the need arise?)	event(Time)			
Frequency<br>Percent<br>Row Pct<br>Col Pct	Pre-survey	Post-survey	Re-test	Total
Entirely unprepared	0<br>0.00<br>.<br>.	0<br>0.00<br>.<br>0.00	0<br>0.00<br>.<br>.	0<br>0.00
Unprepared	0<br>0.00<br>0.00<br>.	1<br>0.27<br>100.00<br>0.27	0<br>0.00<br>0.00<br>.	1<br>0.27
Somewhat unprepared	0<br>0.00<br>0.00<br>.	1<br>0.27<br>100.00<br>0.27	0<br>0.00<br>0.00<br>.	1<br>0.27
Neither prepared nor unprepared	0<br>0.00<br>0.00<br>.	10<br>2.68<br>100.00<br>2.68	0<br>0.00<br>0.00<br>.	10<br>2.68
Somewhat prepared	0<br>0.00<br>0.00<br>.	44<br>11.80<br>100.00<br>11.80	0<br>0.00<br>0.00<br>.	44<br>11.80
Prepared	0<br>0.00<br>0.00<br>.	162<br>43.43<br>100.00<br>43.43	0<br>0.00<br>0.00<br>.	162<br>43.43
Very well prepared	0<br>0.00<br>0.00<br>.	155<br>41.55<br>100.00<br>41.55	0<br>0.00<br>0.00<br>.	155<br>41.55
Total	0<br>0.00	373<br>100.00	0<br>0.00	373<br>100.00
Frequency Missing = 377				
