# Supplementary material for: Educating early childhood care and education providers to improve knowledge and attitudes about reporting child maltreatment: A randomized controlled trial
Source: PLoS One. 2017 May 19;12(5):e0177777. doi: 10.1371/journal.pone.0177777 (PMC5438118; doi:10.1371/journal.pone.0177777)
Supplement: S5 File — (PDF) [file pone.0177777.s006.pdf]

Data dictionary

S5 File Data repository - Attitudes

**Data Set Name**	WORK.ATTITUDE	**Observations**	1858
**Member Type**	DATA	**Variables**	18
**Engine**	V9	**Indexes**	0
**Created**	08/15/2014 10:53:47	**Observation Length**	144
**Last Modified**	08/15/2014 10:53:47	**Deleted Observations**	0
**Protection**		**Compressed**	NO
**Data Set Type**		**Sorted**	NO
**Label**			
**Data Representation**	WINDOWS_64		
**Encoding**	wlatin1 Western (Windows)		

Alphabetic List of Variables and Attributes

#	Variable	Type	Len	Format	Informat	Label
6	attitude_a	Num	8	AGREE.		I might not report child abuse/neglect for fear of backlash from the family or others.
7	attitude_b	Num	8	AGREE.		I might not report child abuse/neglect because it could result in parents doing something bad to their child.
8	attitude_c	Num	8	AGREE.		I would like to fulfill my professional responsibility by reporting cases where I suspect child abuse/neglect.
9	attitude_d	Num	8	AGREE.		Reporting cases of suspected child abuse/neglect improves children’s safety.
10	attitude_e	Num	8	AGREE.		I would not report child abuse/neglect if I thought the child would be removed from his/her family.
11	attitude_f	Num	8	AGREE.		Reporting suspected child abuse/neglect can result in children and families receiving helpful services.
12	attitude_g	Num	8	AGREE.		Early childhood practitioners should be required by law to report suspected child abuse/neglect.
13	attitude_h	Num	8	AGREE.		Children’s long-term interests are promoted by early childhood practitioners reporting suspected child abuse/neglect
14	attitude_i	Num	8	AGREE.		I am concerned that I could be held legally liable for reporting child abuse/neglect that turns out to be unfounded
15	attitude_j	Num	8	AGREE.		It is a waste of time to report child abuse/neglect because often no one follows up on the report
16	attitude_k	Num	8	AGREE.		I should still report suspected child abuse/neglect even if my work supervisor disagrees with me
17	attitude_l	Num	8	AGREE.		I have little confidence that Children and Youth Services will respond effectively if I report suspected child abuse/neglect
18	attitude_m	Num	8	AGREE.		I would not report suspected child abuse/neglect because it is too hard to be sure that abuse occurred
4	date	Num	8	MMDDYY10.		Date of submission
1	dim_user_id	Num	8	BEST12.	BEST32.	dim_user_idect ID

Data dictionary

Alphabetic List of Variables and Attributes						
**#**	**Variable**	**Type**	**Len**	**Format**	**Informat**	**Label**
**2**	event	Num	8	LESSONTYPEID.		Time
**3**	group	Num	8	GRP.		Study group
**5**	time	Num	8	TIME.		Time of submission

Summary

I might not report child abuse/neglect for fear of backlash from the family or others.				
**attitude_a**	**Frequency**	**Percent**	**Cumulative Frequency**	**Cumulative Percent**
**Strongly disagree**	1006	54.17	1006	54.17
**Disagree**	495	26.66	1501	80.83
**Somewhat disagree**	121	6.52	1622	87.35
**Neutral**	128	6.89	1750	94.24
**Somewhat agree**	50	2.69	1800	96.93
**Agree**	32	1.72	1832	98.65
**Strongly agree**	25	1.35	1857	100.00
**Frequency Missing = 1**				

Summary

Table 1 of attitude_a by event				
Controlling for group=Control				
attitude_a(I might not report child abuse/neglect for fear of backlash from the family or others.)	event(Time)			
Frequency<br>Percent<br>Row Pct<br>Col Pct	Pre-survey	Post-survey	Re-test	Total
**Strongly disagree**	160<br>14.45<br>26.76<br>43.13	251<br>22.67<br>41.97<br>68.77	187<br>16.89<br>31.27<br>50.40	598<br>54.02
**Disagree**	109<br>9.85<br>37.98<br>29.38	69<br>6.23<br>24.04<br>18.90	109<br>9.85<br>37.98<br>29.38	287<br>25.93
**Somewhat disagree**	37<br>3.34<br>48.05<br>9.97	14<br>1.26<br>18.18<br>3.84	26<br>2.35<br>33.77<br>7.01	77<br>6.96
**Neutral**	34<br>3.07<br>41.98<br>9.16	19<br>1.72<br>23.46<br>5.21	28<br>2.53<br>34.57<br>7.55	81<br>7.32
**Somewhat agree**	12<br>1.08<br>42.86<br>3.23	6<br>0.54<br>21.43<br>1.64	10<br>0.90<br>35.71<br>2.70	28<br>2.53
**Agree**	8<br>0.72<br>42.11<br>2.16	2<br>0.18<br>10.53<br>0.55	9<br>0.81<br>47.37<br>2.43	19<br>1.72
**Strongly agree**	11<br>0.99<br>64.71<br>2.96	4<br>0.36<br>23.53<br>1.10	2<br>0.18<br>11.76<br>0.54	17<br>1.54
**Total**	371<br>33.51	365<br>32.97	371<br>33.51	1107<br>100.00

Summary

Table 2 of attitude_a by event				
Controlling for group=Experimental				
attitude_a(I might not report child abuse/neglect for fear of backlash from the family or others.)	event(Time)			
Frequency<br>Percent<br>Row Pct<br>Col Pct	Pre-survey	Post-survey	Re-test	Total
**Strongly disagree**	165<br>22.00<br>40.44<br>43.77	243<br>32.40<br>59.56<br>65.15	0<br>0.00<br>0.00<br>.	408<br>54.40
**Disagree**	112<br>14.93<br>53.85<br>29.71	96<br>12.80<br>46.15<br>25.74	0<br>0.00<br>0.00<br>.	208<br>27.73
**Somewhat disagree**	31<br>4.13<br>70.45<br>8.22	13<br>1.73<br>29.55<br>3.49	0<br>0.00<br>0.00<br>.	44<br>5.87
**Neutral**	37<br>4.93<br>78.72<br>9.81	10<br>1.33<br>21.28<br>2.68	0<br>0.00<br>0.00<br>.	47<br>6.27
**Somewhat agree**	20<br>2.67<br>90.91<br>5.31	2<br>0.27<br>9.09<br>0.54	0<br>0.00<br>0.00<br>.	22<br>2.93
**Agree**	7<br>0.93<br>53.85<br>1.86	6<br>0.80<br>46.15<br>1.61	0<br>0.00<br>0.00<br>.	13<br>1.73
**Strongly agree**	5<br>0.67<br>62.50<br>1.33	3<br>0.40<br>37.50<br>0.80	0<br>0.00<br>0.00<br>.	8<br>1.07
**Total**	377<br>50.27	373<br>49.73	0<br>0.00	750<br>100.00
Frequency Missing = 1				

Summary

I might not report child abuse/neglect because it could result in parents doing something bad to their child.				
**attitude_b**	**Frequency**	**Percent**	**Cumulative Frequency**	**Cumulative Percent**
**Strongly disagree**	895	48.20	895	48.20
**Disagree**	509	27.41	1404	75.61
**Somewhat disagree**	169	9.10	1573	84.71
**Neutral**	170	9.15	1743	93.86
**Somewhat agree**	71	3.82	1814	97.68
**Agree**	23	1.24	1837	98.92
**Strongly agree**	20	1.08	1857	100.00
**Frequency Missing = 1**				

Summary

Table 1 of attitude_b by event				
Controlling for group=Control				
attitude_b(I might not report child abuse/neglect because it could result in parents doing something bad to their child.)	event(Time)			
Frequency<br>Percent<br>Row Pct<br>Col Pct	Pre-survey	Post-survey	Re-test	Total
**Strongly disagree**	132<br>11.92<br>25.14<br>35.58	237<br>21.41<br>45.14<br>64.93	156<br>14.09<br>29.71<br>42.05	525<br>47.43
**Disagree**	100<br>9.03<br>33.67<br>26.95	85<br>7.68<br>28.62<br>23.29	112<br>10.12<br>37.71<br>30.19	297<br>26.83
**Somewhat disagree**	58<br>5.24<br>51.79<br>15.63	16<br>1.45<br>14.29<br>4.38	38<br>3.43<br>33.93<br>10.24	112<br>10.12
**Neutral**	48<br>4.34<br>46.15<br>12.94	18<br>1.63<br>17.31<br>4.93	38<br>3.43<br>36.54<br>10.24	104<br>9.39
**Somewhat agree**	21<br>1.90<br>48.84<br>5.66	5<br>0.45<br>11.63<br>1.37	17<br>1.54<br>39.53<br>4.58	43<br>3.88
**Agree**	7<br>0.63<br>50.00<br>1.89	1<br>0.09<br>7.14<br>0.27	6<br>0.54<br>42.86<br>1.62	14<br>1.26
**Strongly agree**	5<br>0.45<br>41.67<br>1.35	3<br>0.27<br>25.00<br>0.82	4<br>0.36<br>33.33<br>1.08	12<br>1.08
**Total**	371<br>33.51	365<br>32.97	371<br>33.51	1107<br>100.00

Summary

Table 2 of attitude_b by event				
Controlling for group=Experimental				
attitude_b(I might not report child abuse/neglect because it could result in parents doing something bad to their child.)	event(Time)			
Frequency<br>Percent<br>Row Pct<br>Col Pct	Pre-survey	Post-survey	Re-test	Total
Strongly disagree	148<br>19.73<br>40.00<br>39.26	222<br>29.60<br>60.00<br>59.52	0<br>0.00<br>0.00<br>.	370<br>49.33
Disagree	110<br>14.67<br>51.89<br>29.18	102<br>13.60<br>48.11<br>27.35	0<br>0.00<br>0.00<br>.	212<br>28.27
Somewhat disagree	37<br>4.93<br>64.91<br>9.81	20<br>2.67<br>35.09<br>5.36	0<br>0.00<br>0.00<br>.	57<br>7.60
Neutral	51<br>6.80<br>77.27<br>13.53	15<br>2.00<br>22.73<br>4.02	0<br>0.00<br>0.00<br>.	66<br>8.80
Somewhat agree	19<br>2.53<br>67.86<br>5.04	9<br>1.20<br>32.14<br>2.41	0<br>0.00<br>0.00<br>.	28<br>3.73
Agree	8<br>1.07<br>88.89<br>2.12	1<br>0.13<br>11.11<br>0.27	0<br>0.00<br>0.00<br>.	9<br>1.20
Strongly agree	4<br>0.53<br>50.00<br>1.06	4<br>0.53<br>50.00<br>1.07	0<br>0.00<br>0.00<br>.	8<br>1.07
Total	377<br>50.27	373<br>49.73	0<br>0.00	750<br>100.00
Frequency Missing = 1				

Summary

I would like to fulfill my professional responsibility by reporting cases where I suspect child abuse/neglect.				
**attitude_c**	**Frequency**	**Percent**	**Cumulative Frequency**	**Cumulative Percent**
**Strongly disagree**	26	1.40	26	1.40
**Disagree**	20	1.08	46	2.48
**Somewhat disagree**	11	0.59	57	3.07
**Neutral**	49	2.64	106	5.71
**Somewhat agree**	123	6.62	229	12.33
**Agree**	442	23.80	671	36.13
**Strongly agree**	1186	63.87	1857	100.00
**Frequency Missing = 1**				

Summary

Table 1 of attitude_c by event				
Controlling for group=Control				
attitude_c(I would like to fulfill my professional responsibility by reporting cases where I suspect child abuse/neglect.)	event(Time)			
Frequency<br>Percent<br>Row Pct<br>Col Pct	Pre-survey	Post-survey	Re-test	Total
**Strongly disagree**	6<br>0.54<br>50.00<br>1.62	3<br>0.27<br>25.00<br>0.82	3<br>0.27<br>25.00<br>0.81	12<br>1.08
**Disagree**	3<br>0.27<br>33.33<br>0.81	2<br>0.18<br>22.22<br>0.55	4<br>0.36<br>44.44<br>1.08	9<br>0.81
**Somewhat disagree**	4<br>0.36<br>80.00<br>1.08	0<br>0.00<br>0.00<br>0.00	1<br>0.09<br>20.00<br>0.27	5<br>0.45
**Neutral**	15<br>1.36<br>45.45<br>4.04	5<br>0.45<br>15.15<br>1.37	13<br>1.17<br>39.39<br>3.50	33<br>2.98
**Somewhat agree**	28<br>2.53<br>36.84<br>7.55	13<br>1.17<br>17.11<br>3.56	35<br>3.16<br>46.05<br>9.43	76<br>6.87
**Agree**	97<br>8.76<br>36.74<br>26.15	69<br>6.23<br>26.14<br>18.90	98<br>8.85<br>37.12<br>26.42	264<br>23.85
**Strongly agree**	218<br>19.69<br>30.79<br>58.76	273<br>24.66<br>38.56<br>74.79	217<br>19.60<br>30.65<br>58.49	708<br>63.96
**Total**	371<br>33.51	365<br>32.97	371<br>33.51	1107<br>100.00

Summary

Table 2 of attitude_c by event				
Controlling for group=Experimental				
attitude_c(I would like to fulfill my professional responsibility by reporting cases where I suspect child abuse/neglect.)	event(Time)			
Frequency<br>Percent<br>Row Pct<br>Col Pct	Pre-survey	Post-survey	Re-test	Total
Strongly disagree	10<br>1.33<br>71.43<br>2.65	4<br>0.53<br>28.57<br>1.07	0<br>0.00<br>0.00<br>.	14<br>1.87
Disagree	8<br>1.07<br>72.73<br>2.12	3<br>0.40<br>27.27<br>0.80	0<br>0.00<br>0.00<br>.	11<br>1.47
Somewhat disagree	4<br>0.53<br>66.67<br>1.06	2<br>0.27<br>33.33<br>0.54	0<br>0.00<br>0.00<br>.	6<br>0.80
Neutral	14<br>1.87<br>87.50<br>3.71	2<br>0.27<br>12.50<br>0.54	0<br>0.00<br>0.00<br>.	16<br>2.13
Somewhat agree	33<br>4.40<br>70.21<br>8.75	14<br>1.87<br>29.79<br>3.75	0<br>0.00<br>0.00<br>.	47<br>6.27
Agree	100<br>13.33<br>56.18<br>26.53	78<br>10.40<br>43.82<br>20.91	0<br>0.00<br>0.00<br>.	178<br>23.73
Strongly agree	208<br>27.73<br>43.51<br>55.17	270<br>36.00<br>56.49<br>72.39	0<br>0.00<br>0.00<br>.	478<br>63.73
Total	377<br>50.27	373<br>49.73	0<br>0.00	750<br>100.00
Frequency Missing = 1				

Summary

Reporting cases of suspected child abuse/neglect improves children’s safety.				
**attitude_d**	**Frequency**	**Percent**	**Cumulative Frequency**	**Cumulative Percent**
**Strongly disagree**	22	1.18	22	1.18
**Disagree**	20	1.08	42	2.26
**Somewhat disagree**	9	0.48	51	2.74
**Neutral**	77	4.14	128	6.89
**Somewhat agree**	131	7.05	259	13.94
**Agree**	477	25.67	736	39.61
**Strongly agree**	1122	60.39	1858	100.00

Summary

Table 1 of attitude_d by event				
Controlling for group=Control				
attitude_d(Reporting cases of suspected child abuse/neglect improves children's safety.)	event(Time)			
Frequency<br>Percent<br>Row Pct<br>Col Pct	Pre-survey	Post-survey	Re-test	Total
Strongly disagree	10<br>0.90<br>58.82<br>2.70	3<br>0.27<br>17.65<br>0.82	4<br>0.36<br>23.53<br>1.08	17<br>1.54
Disagree	6<br>0.54<br>54.55<br>1.62	2<br>0.18<br>18.18<br>0.55	3<br>0.27<br>27.27<br>0.81	11<br>0.99
Somewhat disagree	0<br>0.00<br>0.00<br>0.00	0<br>0.00<br>0.00<br>0.00	2<br>0.18<br>100.00<br>0.54	2<br>0.18
Neutral	25<br>2.26<br>50.00<br>6.74	8<br>0.72<br>16.00<br>2.19	17<br>1.54<br>34.00<br>4.58	50<br>4.52
Somewhat agree	35<br>3.16<br>43.21<br>9.43	12<br>1.08<br>14.81<br>3.29	34<br>3.07<br>41.98<br>9.16	81<br>7.32
Agree	100<br>9.03<br>35.21<br>26.95	79<br>7.14<br>27.82<br>21.64	105<br>9.49<br>36.97<br>28.30	284<br>25.65
Strongly agree	195<br>17.62<br>29.46<br>52.56	261<br>23.58<br>39.43<br>71.51	206<br>18.61<br>31.12<br>55.53	662<br>59.80
Total	371<br>33.51	365<br>32.97	371<br>33.51	1107<br>100.00

Summary

Table 2 of attitude_d by event				
Controlling for group=Experimental				
attitude_d(Reporting cases of suspected child abuse/neglect improves children’s safety.)	event(Time)			
Frequency<br>Percent<br>Row Pct<br>Col Pct	Pre-survey	Post-survey	Re-test	Total
**Strongly disagree**	5<br>0.67<br>100.00<br>1.32	0<br>0.00<br>0.00<br>0.00	0<br>0.00<br>0.00<br>.	5<br>0.67
**Disagree**	7<br>0.93<br>77.78<br>1.85	2<br>0.27<br>22.22<br>0.54	0<br>0.00<br>0.00<br>.	9<br>1.20
**Somewhat disagree**	6<br>0.80<br>85.71<br>1.59	1<br>0.13<br>14.29<br>0.27	0<br>0.00<br>0.00<br>.	7<br>0.93
**Neutral**	22<br>2.93<br>81.48<br>5.82	5<br>0.67<br>18.52<br>1.34	0<br>0.00<br>0.00<br>.	27<br>3.60
**Somewhat agree**	42<br>5.59<br>84.00<br>11.11	8<br>1.07<br>16.00<br>2.14	0<br>0.00<br>0.00<br>.	50<br>6.66
**Agree**	107<br>14.25<br>55.44<br>28.31	86<br>11.45<br>44.56<br>23.06	0<br>0.00<br>0.00<br>.	193<br>25.70
**Strongly agree**	189<br>25.17<br>41.09<br>50.00	271<br>36.09<br>58.91<br>72.65	0<br>0.00<br>0.00<br>.	460<br>61.25
**Total**	378<br>50.33	373<br>49.67	0<br>0.00	751<br>100.00

Summary

I would not report child abuse/neglect if I thought the child would be removed from his/her family.				
attitude_e	Frequency	Percent	Cumulative Frequency	Cumulative Percent
Strongly disagree	1049	56.49	1049	56.49
Disagree	543	29.24	1592	85.73
Somewhat disagree	117	6.30	1709	92.03
Neutral	78	4.20	1787	96.23
Somewhat agree	25	1.35	1812	97.58
Agree	27	1.45	1839	99.03
Strongly agree	18	0.97	1857	100.00
Frequency Missing = 1				

Summary

Table 1 of attitude_e by event				
Controlling for group=Control				
attitude_e(I would not report child abuse/neglect if I thought the child would be removed from his/her family.)	event(Time)			
Frequency<br>Percent<br>Row Pct<br>Col Pct	Pre-survey	Post-survey	Re-test	Total
Strongly disagree	188<br>16.98<br>29.79<br>50.67	253<br>22.85<br>40.10<br>69.32	190<br>17.16<br>30.11<br>51.21	631<br>57.00
Disagree	116<br>10.48<br>38.28<br>31.27	78<br>7.05<br>25.74<br>21.37	109<br>9.85<br>35.97<br>29.38	303<br>27.37
Somewhat disagree	26<br>2.35<br>33.77<br>7.01	20<br>1.81<br>25.97<br>5.48	31<br>2.80<br>40.26<br>8.36	77<br>6.96
Neutral	27<br>2.44<br>46.55<br>7.28	7<br>0.63<br>12.07<br>1.92	24<br>2.17<br>41.38<br>6.47	58<br>5.24
Somewhat agree	7<br>0.63<br>46.67<br>1.89	3<br>0.27<br>20.00<br>0.82	5<br>0.45<br>33.33<br>1.35	15<br>1.36
Agree	4<br>0.36<br>30.77<br>1.08	2<br>0.18<br>15.38<br>0.55	7<br>0.63<br>53.85<br>1.89	13<br>1.17
Strongly agree	3<br>0.27<br>30.00<br>0.81	2<br>0.18<br>20.00<br>0.55	5<br>0.45<br>50.00<br>1.35	10<br>0.90
Total	371<br>33.51	365<br>32.97	371<br>33.51	1107<br>100.00

Summary

Table 2 of attitude_e by event				
Controlling for group=Experimental				
attitude_e(I would not report child abuse/neglect if I thought the child would be removed from his/her family.)	event(Time)			
Frequency<br>Percent<br>Row Pct<br>Col Pct	Pre-survey	Post-survey	Re-test	Total
Strongly disagree	176<br>23.47<br>42.11<br>46.68	242<br>32.27<br>57.89<br>64.88	0<br>0.00<br>0.00<br>.	418<br>55.73
Disagree	138<br>18.40<br>57.50<br>36.60	102<br>13.60<br>42.50<br>27.35	0<br>0.00<br>0.00<br>.	240<br>32.00
Somewhat disagree	32<br>4.27<br>80.00<br>8.49	8<br>1.07<br>20.00<br>2.14	0<br>0.00<br>0.00<br>.	40<br>5.33
Neutral	12<br>1.60<br>60.00<br>3.18	8<br>1.07<br>40.00<br>2.14	0<br>0.00<br>0.00<br>.	20<br>2.67
Somewhat agree	8<br>1.07<br>80.00<br>2.12	2<br>0.27<br>20.00<br>0.54	0<br>0.00<br>0.00<br>.	10<br>1.33
Agree	9<br>1.20<br>64.29<br>2.39	5<br>0.67<br>35.71<br>1.34	0<br>0.00<br>0.00<br>.	14<br>1.87
Strongly agree	2<br>0.27<br>25.00<br>0.53	6<br>0.80<br>75.00<br>1.61	0<br>0.00<br>0.00<br>.	8<br>1.07
Total	377<br>50.27	373<br>49.73	0<br>0.00	750<br>100.00
Frequency Missing = 1				

Summary

Reporting suspected child abuse/neglect can result in children and families receiving helpful services.				
**attitude_f**	**Frequency**	**Percent**	**Cumulative Frequency**	**Cumulative Percent**
**Strongly disagree**	19	1.02	19	1.02
**Disagree**	17	0.92	36	1.94
**Somewhat disagree**	16	0.86	52	2.80
**Neutral**	84	4.52	136	7.32
**Somewhat agree**	198	10.66	334	17.99
**Agree**	498	26.82	832	44.80
**Strongly agree**	1025	55.20	1857	100.00
**Frequency Missing = 1**				

Summary

Table 1 of attitude_f by event				
Controlling for group=Control				
attitude_f(Reporting suspected child abuse/neglect can result in children and families receiving helpful services.)	event(Time)			
Frequency<br>Percent<br>Row Pct<br>Col Pct	Pre-survey	Post-survey	Re-test	Total
**Strongly disagree**	4<br>0.36<br>40.00<br>1.08	2<br>0.18<br>20.00<br>0.55	4<br>0.36<br>40.00<br>1.08	10<br>0.90
**Disagree**	5<br>0.45<br>71.43<br>1.35	1<br>0.09<br>14.29<br>0.27	1<br>0.09<br>14.29<br>0.27	7<br>0.63
**Somewhat disagree**	5<br>0.45<br>45.45<br>1.35	1<br>0.09<br>9.09<br>0.27	5<br>0.45<br>45.45<br>1.35	11<br>0.99
**Neutral**	28<br>2.53<br>47.46<br>7.55	10<br>0.90<br>16.95<br>2.74	21<br>1.90<br>35.59<br>5.66	59<br>5.33
**Somewhat agree**	53<br>4.79<br>44.54<br>14.29	15<br>1.36<br>12.61<br>4.11	51<br>4.61<br>42.86<br>13.75	119<br>10.75
**Agree**	110<br>9.94<br>38.46<br>29.65	83<br>7.50<br>29.02<br>22.74	93<br>8.40<br>32.52<br>25.07	286<br>25.84
**Strongly agree**	166<br>15.00<br>26.99<br>44.74	253<br>22.85<br>41.14<br>69.32	196<br>17.71<br>31.87<br>52.83	615<br>55.56
**Total**	371<br>33.51	365<br>32.97	371<br>33.51	1107<br>100.00

Summary

Table 2 of attitude_f by event				
Controlling for group=Experimental				
attitude_f(Reporting suspected child abuse/neglect can result in children and families receiving helpful services.)	event(Time)			
Frequency<br>Percent<br>Row Pct<br>Col Pct	Pre-survey	Post-survey	Re-test	Total
**Strongly disagree**	5<br>0.67<br>55.56<br>1.33	4<br>0.53<br>44.44<br>1.07	0<br>0.00<br>0.00<br>.	9<br>1.20
**Disagree**	9<br>1.20<br>90.00<br>2.39	1<br>0.13<br>10.00<br>0.27	0<br>0.00<br>0.00<br>.	10<br>1.33
**Somewhat disagree**	2<br>0.27<br>40.00<br>0.53	3<br>0.40<br>60.00<br>0.80	0<br>0.00<br>0.00<br>.	5<br>0.67
**Neutral**	19<br>2.53<br>76.00<br>5.04	6<br>0.80<br>24.00<br>1.61	0<br>0.00<br>0.00<br>.	25<br>3.33
**Somewhat agree**	66<br>8.80<br>83.54<br>17.51	13<br>1.73<br>16.46<br>3.49	0<br>0.00<br>0.00<br>.	79<br>10.53
**Agree**	108<br>14.40<br>50.94<br>28.65	104<br>13.87<br>49.06<br>27.88	0<br>0.00<br>0.00<br>.	212<br>28.27
**Strongly agree**	168<br>22.40<br>40.98<br>44.56	242<br>32.27<br>59.02<br>64.88	0<br>0.00<br>0.00<br>.	410<br>54.67
**Total**	377<br>50.27	373<br>49.73	0<br>0.00	750<br>100.00
Frequency Missing = 1				

Summary

Early childhood practitioners should be required by law to report suspected child abuse/neglect.				
**attitude_g**	**Frequency**	**Percent**	**Cumulative Frequency**	**Cumulative Percent**
**Strongly disagree**	27	1.45	27	1.45
**Disagree**	13	0.70	40	2.15
**Somewhat disagree**	15	0.81	55	2.96
**Neutral**	54	2.91	109	5.87
**Somewhat agree**	85	4.58	194	10.45
**Agree**	412	22.19	606	32.63
**Strongly agree**	1251	67.37	1857	100.00
**Frequency Missing = 1**				

Summary

Table 1 of attitude_g by event				
Controlling for group=Control				
attitude_g(Early childhood practitioners should be required by law to report suspected child abuse/neglect.)	event(Time)			
Frequency<br>Percent<br>Row Pct<br>Col Pct	Pre-survey	Post-survey	Re-test	Total
Strongly disagree	8<br>0.72<br>53.33<br>2.16	3<br>0.27<br>20.00<br>0.82	4<br>0.36<br>26.67<br>1.08	15<br>1.36
Disagree	4<br>0.36<br>44.44<br>1.08	1<br>0.09<br>11.11<br>0.27	4<br>0.36<br>44.44<br>1.08	9<br>0.81
Somewhat disagree	3<br>0.27<br>75.00<br>0.81	0<br>0.00<br>0.00<br>0.00	1<br>0.09<br>25.00<br>0.27	4<br>0.36
Neutral	18<br>1.63<br>51.43<br>4.85	3<br>0.27<br>8.57<br>0.82	14<br>1.26<br>40.00<br>3.77	35<br>3.16
Somewhat agree	21<br>1.90<br>35.59<br>5.66	11<br>0.99<br>18.64<br>3.01	27<br>2.44<br>45.76<br>7.28	59<br>5.33
Agree	95<br>8.58<br>39.26<br>25.61	70<br>6.32<br>28.93<br>19.18	77<br>6.96<br>31.82<br>20.75	242<br>21.86
Strongly agree	222<br>20.05<br>29.88<br>59.84	277<br>25.02<br>37.28<br>75.89	244<br>22.04<br>32.84<br>65.77	743<br>67.12
Total	371<br>33.51	365<br>32.97	371<br>33.51	1107<br>100.00

Summary

Table 2 of attitude_g by event				
Controlling for group=Experimental				
attitude_g(Early childhood practitioners should be required by law to report suspected child abuse/neglect.)	event(Time)			
Frequency<br>Percent<br>Row Pct<br>Col Pct	Pre-survey	Post-survey	Re-test	Total
Strongly disagree	9<br>1.20<br>75.00<br>2.39	3<br>0.40<br>25.00<br>0.80	0<br>0.00<br>0.00<br>.	12<br>1.60
Disagree	3<br>0.40<br>75.00<br>0.80	1<br>0.13<br>25.00<br>0.27	0<br>0.00<br>0.00<br>.	4<br>0.53
Somewhat disagree	9<br>1.20<br>81.82<br>2.39	2<br>0.27<br>18.18<br>0.54	0<br>0.00<br>0.00<br>.	11<br>1.47
Neutral	13<br>1.73<br>68.42<br>3.45	6<br>0.80<br>31.58<br>1.61	0<br>0.00<br>0.00<br>.	19<br>2.53
Somewhat agree	15<br>2.00<br>57.69<br>3.98	11<br>1.47<br>42.31<br>2.95	0<br>0.00<br>0.00<br>.	26<br>3.47
Agree	93<br>12.40<br>54.71<br>24.67	77<br>10.27<br>45.29<br>20.64	0<br>0.00<br>0.00<br>.	170<br>22.67
Strongly agree	235<br>31.33<br>46.26<br>62.33	273<br>36.40<br>53.74<br>73.19	0<br>0.00<br>0.00<br>.	508<br>67.73
Total	377<br>50.27	373<br>49.73	0<br>0.00	750<br>100.00
Frequency Missing = 1				

Summary

Children's long-term interests are promoted by early childhood practitioners reporting suspected child abuse/neglect				
attitude_h	Frequency	Percent	Cumulative Frequency	Cumulative Percent
Strongly disagree	38	2.05	38	2.05
Disagree	31	1.67	69	3.72
Somewhat disagree	29	1.56	98	5.28
Neutral	186	10.02	284	15.29
Somewhat agree	210	11.31	494	26.60
Agree	470	25.31	964	51.91
Strongly agree	893	48.09	1857	100.00
Frequency Missing = 1				

Summary

Table 1 of attitude_h by event				
Controlling for group=Control				
attitude_h(Children's long-term interests are promoted by early childhood practitioners reporting suspected child abuse/neglect)	event(Time)			
Frequency<br>Percent<br>Row Pct<br>Col Pct	Pre-survey	Post-survey	Re-test	Total
Strongly disagree	11<br>0.99<br>45.83<br>2.96	5<br>0.45<br>20.83<br>1.37	8<br>0.72<br>33.33<br>2.16	24<br>2.17
Disagree	10<br>0.90<br>45.45<br>2.70	6<br>0.54<br>27.27<br>1.64	6<br>0.54<br>27.27<br>1.62	22<br>1.99
Somewhat disagree	8<br>0.72<br>61.54<br>2.16	1<br>0.09<br>7.69<br>0.27	4<br>0.36<br>30.77<br>1.08	13<br>1.17
Neutral	57<br>5.15<br>49.14<br>15.36	13<br>1.17<br>11.21<br>3.56	46<br>4.16<br>39.66<br>12.40	116<br>10.48
Somewhat agree	57<br>5.15<br>43.18<br>15.36	29<br>2.62<br>21.97<br>7.95	46<br>4.16<br>34.85<br>12.40	132<br>11.92
Agree	94<br>8.49<br>35.07<br>25.34	78<br>7.05<br>29.10<br>21.37	96<br>8.67<br>35.82<br>25.88	268<br>24.21
Strongly agree	134<br>12.10<br>25.19<br>36.12	233<br>21.05<br>43.80<br>63.84	165<br>14.91<br>31.02<br>44.47	532<br>48.06
Total	371<br>33.51	365<br>32.97	371<br>33.51	1107<br>100.00

Summary

Table 2 of attitude_h by event				
Controlling for group=Experimental				
attitude_h(Children's long-term interests are promoted by early childhood practitioners reporting suspected child abuse/neglect)	event(Time)			
Frequency<br>Percent<br>Row Pct<br>Col Pct	Pre-survey	Post-survey	Re-test	Total
**Strongly disagree**	8<br>1.07<br>57.14<br>2.12	6<br>0.80<br>42.86<br>1.61	0<br>0.00<br>0.00<br>.	14<br>1.87
**Disagree**	7<br>0.93<br>77.78<br>1.86	2<br>0.27<br>22.22<br>0.54	0<br>0.00<br>0.00<br>.	9<br>1.20
**Somewhat disagree**	12<br>1.60<br>75.00<br>3.18	4<br>0.53<br>25.00<br>1.07	0<br>0.00<br>0.00<br>.	16<br>2.13
**Neutral**	57<br>7.60<br>81.43<br>15.12	13<br>1.73<br>18.57<br>3.49	0<br>0.00<br>0.00<br>.	70<br>9.33
**Somewhat agree**	55<br>7.33<br>70.51<br>14.59	23<br>3.07<br>29.49<br>6.17	0<br>0.00<br>0.00<br>.	78<br>10.40
**Agree**	109<br>14.53<br>53.96<br>28.91	93<br>12.40<br>46.04<br>24.93	0<br>0.00<br>0.00<br>.	202<br>26.93
**Strongly agree**	129<br>17.20<br>35.73<br>34.22	232<br>30.93<br>64.27<br>62.20	0<br>0.00<br>0.00<br>.	361<br>48.13
**Total**	377<br>50.27	373<br>49.73	0<br>0.00	750<br>100.00
Frequency Missing = 1				

Summary

I am concerned that I could be held legally liable for reporting child abuse/neglect that turns out to be unfounded				
attitude_i	Frequency	Percent	Cumulative Frequency	Cumulative Percent
Strongly disagree	818	44.05	818	44.05
Disagree	373	20.09	1191	64.14
Somewhat disagree	132	7.11	1323	71.24
Neutral	254	13.68	1577	84.92
Somewhat agree	123	6.62	1700	91.55
Agree	91	4.90	1791	96.45
Strongly agree	66	3.55	1857	100.00
Frequency Missing = 1				

Summary

Table 1 of attitude_i by event				
Controlling for group=Control				
attitude_i(I am concerned that I could be held legally liable for reporting child abuse/neglect that turns out to be unfounded)	event(Time)			
Frequency<br>Percent<br>Row Pct<br>Col Pct	Pre-survey	Post-survey	Re-test	Total
Strongly disagree	120<br>10.84<br>24.49<br>32.35	231<br>20.87<br>47.14<br>63.29	139<br>12.56<br>28.37<br>37.47	490<br>44.26
Disagree	64<br>5.78<br>30.48<br>17.25	61<br>5.51<br>29.05<br>16.71	85<br>7.68<br>40.48<br>22.91	210<br>18.97
Somewhat disagree	39<br>3.52<br>53.42<br>10.51	14<br>1.26<br>19.18<br>3.84	20<br>1.81<br>27.40<br>5.39	73<br>6.59
Neutral	67<br>6.05<br>43.23<br>18.06	19<br>1.72<br>12.26<br>5.21	69<br>6.23<br>44.52<br>18.60	155<br>14.00
Somewhat agree	40<br>3.61<br>49.38<br>10.78	12<br>1.08<br>14.81<br>3.29	29<br>2.62<br>35.80<br>7.82	81<br>7.32
Agree	27<br>2.44<br>45.76<br>7.28	13<br>1.17<br>22.03<br>3.56	19<br>1.72<br>32.20<br>5.12	59<br>5.33
Strongly agree	14<br>1.26<br>35.90<br>3.77	15<br>1.36<br>38.46<br>4.11	10<br>0.90<br>25.64<br>2.70	39<br>3.52
Total	371<br>33.51	365<br>32.97	371<br>33.51	1107<br>100.00

Summary

Table 2 of attitude_i by event				
Controlling for group=Experimental				
attitude_i(I am concerned that I could be held legally liable for reporting child abuse/neglect that turns out to be unfounded)	event(Time)			
Frequency<br>Percent<br>Row Pct<br>Col Pct	Pre-survey	Post-survey	Re-test	Total
Strongly disagree	108<br>14.40<br>32.93<br>28.65	220<br>29.33<br>67.07<br>58.98	0<br>0.00<br>0.00<br>.	328<br>43.73
Disagree	77<br>10.27<br>47.24<br>20.42	86<br>11.47<br>52.76<br>23.06	0<br>0.00<br>0.00<br>.	163<br>21.73
Somewhat disagree	46<br>6.13<br>77.97<br>12.20	13<br>1.73<br>22.03<br>3.49	0<br>0.00<br>0.00<br>.	59<br>7.87
Neutral	74<br>9.87<br>74.75<br>19.63	25<br>3.33<br>25.25<br>6.70	0<br>0.00<br>0.00<br>.	99<br>13.20
Somewhat agree	32<br>4.27<br>76.19<br>8.49	10<br>1.33<br>23.81<br>2.68	0<br>0.00<br>0.00<br>.	42<br>5.60
Agree	23<br>3.07<br>71.88<br>6.10	9<br>1.20<br>28.13<br>2.41	0<br>0.00<br>0.00<br>.	32<br>4.27
Strongly agree	17<br>2.27<br>62.96<br>4.51	10<br>1.33<br>37.04<br>2.68	0<br>0.00<br>0.00<br>.	27<br>3.60
Total	377<br>50.27	373<br>49.73	0<br>0.00	750<br>100.00
Frequency Missing = 1				

Summary

It is a waste of time to report child abuse/neglect because often no one follows up on the report				
**attitude_j**	**Frequency**	**Percent**	**Cumulative Frequency**	**Cumulative Percent**
**Strongly disagree**	1071	57.67	1071	57.67
**Disagree**	513	27.63	1584	85.30
**Somewhat disagree**	103	5.55	1687	90.85
**Neutral**	95	5.12	1782	95.96
**Somewhat agree**	38	2.05	1820	98.01
**Agree**	27	1.45	1847	99.46
**Strongly agree**	10	0.54	1857	100.00
**Frequency Missing = 1**				

Summary

Table 1 of attitude_j by event				
Controlling for group=Control				
attitude_j(It is a waste of time to report child abuse/neglect because often no one follows up on the report)	event(Time)			
Frequency<br>Percent<br>Row Pct<br>Col Pct	Pre-survey	Post-survey	Re-test	Total
Strongly disagree	185<br>16.71<br>29.55<br>49.87	251<br>22.67<br>40.10<br>68.77	190<br>17.16<br>30.35<br>51.21	626<br>56.55
Disagree	109<br>9.85<br>35.28<br>29.38	86<br>7.77<br>27.83<br>23.56	114<br>10.30<br>36.89<br>30.73	309<br>27.91
Somewhat disagree	30<br>2.71<br>45.45<br>8.09	11<br>0.99<br>16.67<br>3.01	25<br>2.26<br>37.88<br>6.74	66<br>5.96
Neutral	30<br>2.71<br>48.39<br>8.09	7<br>0.63<br>11.29<br>1.92	25<br>2.26<br>40.32<br>6.74	62<br>5.60
Somewhat agree	11<br>0.99<br>40.74<br>2.96	7<br>0.63<br>25.93<br>1.92	9<br>0.81<br>33.33<br>2.43	27<br>2.44
Agree	4<br>0.36<br>33.33<br>1.08	2<br>0.18<br>16.67<br>0.55	6<br>0.54<br>50.00<br>1.62	12<br>1.08
Strongly agree	2<br>0.18<br>40.00<br>0.54	1<br>0.09<br>20.00<br>0.27	2<br>0.18<br>40.00<br>0.54	5<br>0.45
Total	371<br>33.51	365<br>32.97	371<br>33.51	1107<br>100.00

Summary

Table 2 of attitude_j by event				
Controlling for group=Experimental				
attitude_j(It is a waste of time to report child abuse/neglect because often no one follows up on the report)	event(Time)			
Frequency<br>Percent<br>Row Pct<br>Col Pct	Pre-survey	Post-survey	Re-test	Total
**Strongly disagree**	185<br>24.67<br>41.57<br>49.07	260<br>34.67<br>58.43<br>69.71	0<br>0.00<br>0.00<br>.	445<br>59.33
**Disagree**	119<br>15.87<br>58.33<br>31.56	85<br>11.33<br>41.67<br>22.79	0<br>0.00<br>0.00<br>.	204<br>27.20
**Somewhat disagree**	25<br>3.33<br>67.57<br>6.63	12<br>1.60<br>32.43<br>3.22	0<br>0.00<br>0.00<br>.	37<br>4.93
**Neutral**	29<br>3.87<br>87.88<br>7.69	4<br>0.53<br>12.12<br>1.07	0<br>0.00<br>0.00<br>.	33<br>4.40
**Somewhat agree**	8<br>1.07<br>72.73<br>2.12	3<br>0.40<br>27.27<br>0.80	0<br>0.00<br>0.00<br>.	11<br>1.47
**Agree**	8<br>1.07<br>53.33<br>2.12	7<br>0.93<br>46.67<br>1.88	0<br>0.00<br>0.00<br>.	15<br>2.00
**Strongly agree**	3<br>0.40<br>60.00<br>0.80	2<br>0.27<br>40.00<br>0.54	0<br>0.00<br>0.00<br>.	5<br>0.67
**Total**	377<br>50.27	373<br>49.73	0<br>0.00	750<br>100.00
Frequency Missing = 1				

Summary

I should still report suspected child abuse/neglect even if my work supervisor disagrees with me				
**attitude_k**	**Frequency**	**Percent**	**Cumulative Frequency**	**Cumulative Percent**
**Strongly disagree**	73	3.93	73	3.93
**Disagree**	51	2.75	124	6.68
**Somewhat disagree**	56	3.02	180	9.69
**Neutral**	225	12.12	405	21.81
**Somewhat agree**	219	11.79	624	33.60
**Agree**	421	22.67	1045	56.27
**Strongly agree**	812	43.73	1857	100.00
**Frequency Missing = 1**				

Summary

Table 1 of attitude_k by event				
Controlling for group=Control				
attitude_k(I should still report suspected child abuse/neglect even if my work supervisor disagrees with me)	event(Time)			
Frequency<br>Percent<br>Row Pct<br>Col Pct	Pre-survey	Post-survey	Re-test	Total
Strongly disagree	17<br>1.54<br>41.46<br>4.58	12<br>1.08<br>29.27<br>3.29	12<br>1.08<br>29.27<br>3.23	41<br>3.70
Disagree	11<br>0.99<br>31.43<br>2.96	5<br>0.45<br>14.29<br>1.37	19<br>1.72<br>54.29<br>5.12	35<br>3.16
Somewhat disagree	15<br>1.36<br>46.88<br>4.04	1<br>0.09<br>3.13<br>0.27	16<br>1.45<br>50.00<br>4.31	32<br>2.89
Neutral	68<br>6.14<br>49.64<br>18.33	7<br>0.63<br>5.11<br>1.92	62<br>5.60<br>45.26<br>16.71	137<br>12.38
Somewhat agree	70<br>6.32<br>47.62<br>18.87	21<br>1.90<br>14.29<br>5.75	56<br>5.06<br>38.10<br>15.09	147<br>13.28
Agree	74<br>6.68<br>31.49<br>19.95	83<br>7.50<br>35.32<br>22.74	78<br>7.05<br>33.19<br>21.02	235<br>21.23
Strongly agree	116<br>10.48<br>24.17<br>31.27	236<br>21.32<br>49.17<br>64.66	128<br>11.56<br>26.67<br>34.50	480<br>43.36
Total	371<br>33.51	365<br>32.97	371<br>33.51	1107<br>100.00

Summary

Table 2 of attitude_k by event				
Controlling for group=Experimental				
attitude_k(I should still report suspected child abuse/neglect even if my work supervisor disagrees with me)	event(Time)			
Frequency<br>Percent<br>Row Pct<br>Col Pct	Pre-survey	Post-survey	Re-test	Total
Strongly disagree	18<br>2.40<br>56.25<br>4.77	14<br>1.87<br>43.75<br>3.75	0<br>0.00<br>0.00<br>.	32<br>4.27
Disagree	15<br>2.00<br>93.75<br>3.98	1<br>0.13<br>6.25<br>0.27	0<br>0.00<br>0.00<br>.	16<br>2.13
Somewhat disagree	18<br>2.40<br>75.00<br>4.77	6<br>0.80<br>25.00<br>1.61	0<br>0.00<br>0.00<br>.	24<br>3.20
Neutral	70<br>9.33<br>79.55<br>18.57	18<br>2.40<br>20.45<br>4.83	0<br>0.00<br>0.00<br>.	88<br>11.73
Somewhat agree	52<br>6.93<br>72.22<br>13.79	20<br>2.67<br>27.78<br>5.36	0<br>0.00<br>0.00<br>.	72<br>9.60
Agree	94<br>12.53<br>50.54<br>24.93	92<br>12.27<br>49.46<br>24.66	0<br>0.00<br>0.00<br>.	186<br>24.80
Strongly agree	110<br>14.67<br>33.13<br>29.18	222<br>29.60<br>66.87<br>59.52	0<br>0.00<br>0.00<br>.	332<br>44.27
Total	377<br>50.27	373<br>49.73	0<br>0.00	750<br>100.00
Frequency Missing = 1				

Summary

I have little confidence that Children and Youth Services will respond effectively if I report suspected child abuse/neglect				
**attitude_l**	**Frequency**	**Percent**	**Cumulative Frequency**	**Cumulative Percent**
**Strongly disagree**	754	40.60	754	40.60
**Disagree**	498	26.82	1252	67.42
**Somewhat disagree**	184	9.91	1436	77.33
**Neutral**	200	10.77	1636	88.10
**Somewhat agree**	91	4.90	1727	93.00
**Agree**	83	4.47	1810	97.47
**Strongly agree**	47	2.53	1857	100.00
**Frequency Missing = 1**				

Summary

Table 1 of attitude_l by event				
Controlling for group=Control				
attitude_l(I have little confidence that Children and Youth Services will respond effectively if I report suspected child abuse/neglect)	event(Time)			
Frequency<br>Percent<br>Row Pct<br>Col Pct	Pre-survey	Post-survey	Re-test	Total
Strongly disagree	107<br>9.67<br>23.62<br>28.84	216<br>19.51<br>47.68<br>59.18	130<br>11.74<br>28.70<br>35.04	453<br>40.92
Disagree	94<br>8.49<br>32.75<br>25.34	84<br>7.59<br>29.27<br>23.01	109<br>9.85<br>37.98<br>29.38	287<br>25.93
Somewhat disagree	53<br>4.79<br>46.09<br>14.29	23<br>2.08<br>20.00<br>6.30	39<br>3.52<br>33.91<br>10.51	115<br>10.39
Neutral	59<br>5.33<br>48.36<br>15.90	16<br>1.45<br>13.11<br>4.38	47<br>4.25<br>38.52<br>12.67	122<br>11.02
Somewhat agree	26<br>2.35<br>50.98<br>7.01	9<br>0.81<br>17.65<br>2.47	16<br>1.45<br>31.37<br>4.31	51<br>4.61
Agree	18<br>1.63<br>37.50<br>4.85	9<br>0.81<br>18.75<br>2.47	21<br>1.90<br>43.75<br>5.66	48<br>4.34
Strongly agree	14<br>1.26<br>45.16<br>3.77	8<br>0.72<br>25.81<br>2.19	9<br>0.81<br>29.03<br>2.43	31<br>2.80
Total	371<br>33.51	365<br>32.97	371<br>33.51	1107<br>100.00

Summary

Table 2 of attitude_l by event				
Controlling for group=Experimental				
attitude_l(I have little confidence that Children and Youth Services will respond effectively if I report suspected child abuse/neglect)	event(Time)			
Frequency<br>Percent<br>Row Pct<br>Col Pct	Pre-survey	Post-survey	Re-test	Total
**Strongly disagree**	104<br>13.87<br>34.55<br>27.59	197<br>26.27<br>65.45<br>52.82	0<br>0.00<br>0.00<br>.	301<br>40.13
**Disagree**	103<br>13.73<br>48.82<br>27.32	108<br>14.40<br>51.18<br>28.95	0<br>0.00<br>0.00<br>.	211<br>28.13
**Somewhat disagree**	50<br>6.67<br>72.46<br>13.26	19<br>2.53<br>27.54<br>5.09	0<br>0.00<br>0.00<br>.	69<br>9.20
**Neutral**	64<br>8.53<br>82.05<br>16.98	14<br>1.87<br>17.95<br>3.75	0<br>0.00<br>0.00<br>.	78<br>10.40
**Somewhat agree**	27<br>3.60<br>67.50<br>7.16	13<br>1.73<br>32.50<br>3.49	0<br>0.00<br>0.00<br>.	40<br>5.33
**Agree**	20<br>2.67<br>57.14<br>5.31	15<br>2.00<br>42.86<br>4.02	0<br>0.00<br>0.00<br>.	35<br>4.67
**Strongly agree**	9<br>1.20<br>56.25<br>2.39	7<br>0.93<br>43.75<br>1.88	0<br>0.00<br>0.00<br>.	16<br>2.13
**Total**	377<br>50.27	373<br>49.73	0<br>0.00	750<br>100.00
Frequency Missing = 1				

Summary

I would not report suspected child abuse/neglect because it is too hard to be sure that abuse occurred				
**attitude_m**	**Frequency**	**Percent**	**Cumulative Frequency**	**Cumulative Percent**
**Strongly disagree**	901	48.52	901	48.52
**Disagree**	531	28.59	1432	77.11
**Somewhat disagree**	197	10.61	1629	87.72
**Neutral**	141	7.59	1770	95.32
**Somewhat agree**	38	2.05	1808	97.36
**Agree**	27	1.45	1835	98.82
**Strongly agree**	22	1.18	1857	100.00
**Frequency Missing = 1**				

Summary

Table 1 of attitude_m by event				
Controlling for group=Control				
attitude_m(I would not report suspected child abuse/neglect because it is too hard to be sure that abuse occurred)	event(Time)			
Frequency<br>Percent<br>Row Pct<br>Col Pct	Pre-survey	Post-survey	Re-test	Total
**Strongly disagree**	129<br>11.65<br>24.62<br>34.77	237<br>21.41<br>45.23<br>64.93	158<br>14.27<br>30.15<br>42.59	524<br>47.34
**Disagree**	110<br>9.94<br>35.71<br>29.65	92<br>8.31<br>29.87<br>25.21	106<br>9.58<br>34.42<br>28.57	308<br>27.82
**Somewhat disagree**	56<br>5.06<br>45.90<br>15.09	19<br>1.72<br>15.57<br>5.21	47<br>4.25<br>38.52<br>12.67	122<br>11.02
**Neutral**	53<br>4.79<br>53.00<br>14.29	9<br>0.81<br>9.00<br>2.47	38<br>3.43<br>38.00<br>10.24	100<br>9.03
**Somewhat agree**	12<br>1.08<br>57.14<br>3.23	2<br>0.18<br>9.52<br>0.55	7<br>0.63<br>33.33<br>1.89	21<br>1.90
**Agree**	5<br>0.45<br>27.78<br>1.35	3<br>0.27<br>16.67<br>0.82	10<br>0.90<br>55.56<br>2.70	18<br>1.63
**Strongly agree**	6<br>0.54<br>42.86<br>1.62	3<br>0.27<br>21.43<br>0.82	5<br>0.45<br>35.71<br>1.35	14<br>1.26
**Total**	371<br>33.51	365<br>32.97	371<br>33.51	1107<br>100.00

Summary

Table 2 of attitude_m by event				
Controlling for group=Experimental				
attitude_m(I would not report suspected child abuse/neglect because it is too hard to be sure that abuse occurred)	event(Time)			
Frequency<br>Percent<br>Row Pct<br>Col Pct	Pre-survey	Post-survey	Re-test	Total
Strongly disagree	152<br>20.27<br>40.32<br>40.32	225<br>30.00<br>59.68<br>60.32	0<br>0.00<br>0.00<br>.	377<br>50.27
Disagree	115<br>15.33<br>51.57<br>30.50	108<br>14.40<br>48.43<br>28.95	0<br>0.00<br>0.00<br>.	223<br>29.73
Somewhat disagree	57<br>7.60<br>76.00<br>15.12	18<br>2.40<br>24.00<br>4.83	0<br>0.00<br>0.00<br>.	75<br>10.00
Neutral	32<br>4.27<br>78.05<br>8.49	9<br>1.20<br>21.95<br>2.41	0<br>0.00<br>0.00<br>.	41<br>5.47
Somewhat agree	12<br>1.60<br>70.59<br>3.18	5<br>0.67<br>29.41<br>1.34	0<br>0.00<br>0.00<br>.	17<br>2.27
Agree	5<br>0.67<br>55.56<br>1.33	4<br>0.53<br>44.44<br>1.07	0<br>0.00<br>0.00<br>.	9<br>1.20
Strongly agree	4<br>0.53<br>50.00<br>1.06	4<br>0.53<br>50.00<br>1.07	0<br>0.00<br>0.00<br>.	8<br>1.07
Total	377<br>50.27	373<br>49.73	0<br>0.00	750<br>100.00
Frequency Missing = 1				
